# Association of Diverse Staphylococcus aureus Populations with Pseudomonas aeruginosa Coinfection and Inflammation in Cystic Fibrosis Airway Infection

**DOI:** 10.1128/mSphere.00358-21

**Published:** 2021-06-23

**Authors:** Marie K. Wieneke, Felix Dach, Claudia Neumann, Dennis Görlich, Lena Kaese, Theo Thißen, Angelika Dübbers, Christina Kessler, Jörg Große-Onnebrink, Peter Küster, Holger Schültingkemper, Bianca Schwartbeck, Johannes Roth, Jerzy-Roch Nofer, Janina Treffon, Julia Posdorfer, Josefine Marie Boecken, Mariele Strake, Miriam Abdo, Sophia Westhues, Barbara C. Kahl

**Affiliations:** a Institute of Medical Microbiology, University Hospital Münster, Münster, Germany; b Institute of Biostatistics and Clinical Research, University Hospital Münster, Münster, Germany; c Department of Pediatrics, University Hospital Münster, Münster, Germany; d Department of Pediatrics, Clemenshospital Münster, Münster, Germany; e Institute of Immunology, University Hospital Münster, Münster, Germany; f Central Laboratory Facility, University Hospital Münster, Münster, Germany; g Institute of Hygiene, University Hospital Münster, Münster, Germany; University of Nebraska Medical Center

**Keywords:** *Pseudomonas aeruginosa*, *Staphylococcus aureus*, cystic fibrosis, diversity, persistent infection

## Abstract

Staphylococcus aureus is one of the most common pathogens isolated from the airways of cystic fibrosis (CF) patients and often persists for extended periods. There is limited knowledge about the diversity of S. aureus in CF. We hypothesized that increased diversity of S. aureus would impact CF lung disease. Therefore, we conducted a 1-year observational prospective study with 14 patients with long-term S. aureus infection. From every sputum, 40 S. aureus isolates were chosen and characterized in terms of phenotypic appearance (size, hemolysis, mucoidy, and pigmentation), important virulence traits such as nuclease activity, biofilm formation, and molecular typing by *spa* sequence typing. Data about coinfection with Pseudomonas aeruginosa and clinical parameters such as lung function, exacerbation, and inflammatory markers in blood (C-reactive protein [CRP], interleukin 6 [IL-6], and S100A8/9 [calprotectin]) were collected. From 58 visits of 14 patients, 2,319 S. aureus isolates were distinguished into 32 phenotypes (PTs) and 50 *spa* types. The Simpson diversity index (SDI) was used to calculate the phenotypic and genotypic diversity, revealing a high diversity of PTs ranging from 0.19 to 0.87 among patients, while the diversity of *spa* types of isolates was less pronounced. The SDI of PTs was positively associated with P. aeruginosa coinfection and inflammatory parameters, with IL-6 being the most sensitive parameter. Also, coinfection with P. aeruginosa was associated with mucoid S. aureus and S. aureus with high nuclease activity. Our analyses showed that in CF patients with long-term S. aureus airway infection, a highly diverse and dynamic S. aureus population was present and associated with P. aeruginosa coinfection and inflammation.

**IMPORTANCE** Staphylococcus aureus can persist for extended periods in the airways of people with cystic fibrosis (CF) in spite of antibiotic therapy and high numbers of neutrophils, which fail to eradicate this pathogen. Therefore, S. aureus needs to adapt to this hostile niche. There is only limited knowledge about the diversity of S. aureus in respiratory specimens. We conducted a 1-year prospective study with 14 patients with long-term S. aureus infection and investigated 40 S. aureus isolates from every sputum in terms of phenotypic appearance, nuclease activity, biofilm formation, and molecular typing. Data about coinfection with Pseudomonas aeruginosa and clinical parameters such as lung function, exacerbation, and inflammatory markers in blood were collected. Thirty-two phenotypes (PTs) and 50 *spa* types were distinguished. Our analyses revealed that in CF patients with long-term S. aureus airway infection, a highly diverse and dynamic S. aureus population was associated with P. aeruginosa coinfection and inflammation.

## INTRODUCTION

Abnormal respiratory secretions in cystic fibrosis (CF), the most common genetic disease in the Caucasian population, are the basis for chronic and recurrent bacterial lung infections (1). Today, Pseudomonas aeruginosa and Staphylococcus aureus are the two most common pathogens within the airways of CF patients (2, 3). S. aureus is also one of the most common pathogens responsible for community- and hospital-acquired infections, including pneumonia (4). S. aureus is equipped with many virulence factors, which facilitate colonization leading to infection, bacteremia, and sepsis (4). For pneumonia, some virulence factors are of particular importance such as α-toxin (Hla), which plays a pivotal role in S. aureus pneumonia in mouse and rat models and which is able to lyse various eukaryotic cells, including erythrocytes and pneumocytes (5). In addition, β-toxin (Hlb), which functions as a sphingomyelinase, can induce severe lung injury (6). In most clinical S. aureus isolates, *hlb* is disrupted by a phage, which inserts into the *hlb* gene and carries a number of genes, which confer innate immune evasion (7).

In CF lung disease, the same S. aureus clone often persists for many years (8, 9). To resist the hostile environment of CF airways with high numbers of neutrophils (10), coinfecting pathogens like P. aeruginosa (11, 12) and S. aureus adapt to the airways by different strategies, such as the following: (i) the emergence of small colony variants (SCVs) (13, 14); (ii) the emergence of mucoid colonies, which synthesize constitutively large amounts of biofilm, a feature that has been reported so far for P. aeruginosa (15) but only rarely for S. aureus (16, 17); (iii) increased activity of the secreted nuclease (18), which enables S. aureus to escape from neutrophil extracellular traps (NETs) (19) present in CF lung disease (20); (iv) increased expression of superoxide dismutase M, which facilitates S. aureus survival during attack of neutrophils with oxygen radicals (21, 22); and (v) increased biofilm formation as a response to the changed immunometabolic status of the airways (23).

Although whole-genome sequencing is common today for the molecular analysis of S. aureus isolates in CF patients (24, 25), sequencing of the variable number of tandem repeats of protein A of S. aureus (*spa* sequencing) remains a valuable tool, which not only provides information about transmission of clones in a special setting (26) but also allows us to observe microevolutionary changes during persistence with deletions, point mutations, or insertions occurring within the repeat regions of related isolates (16, 18, 27).

Current knowledge related to the diversity of S. aureus phenotypes during persistent respiratory infection in CF is scarce. We hypothesized that diversity of S. aureus phenotypes would be associated with CF lung disease. Therefore, we conducted a prospective 1-year study to investigate the diversity of S. aureus populating the airways of CF patients by cultural in-depth analysis: From every sputum, 40 S. aureus isolates were collected from patients with long-term persistent S. aureus infection. Such isolates were phenotypically characterized in regard to size (normal/SCV), hemolysis (α- and β-toxin), mucoidy and pigmentation of colonies. Furthermore, all isolates were molecular typed by *spa* sequence typing and assessed for two important virulence traits comprising the activity of staphylococcal nuclease and the amount of biofilm formation. For all visits, information about P. aeruginosa coinfection and clinical data were collected, which included data about lung function, antibiotic treatment, and determination of inflammatory parameters in sera (S100A8/9 [also known as calprotectin], interleukin 6 [IL-6], and C-reactive protein [CRP]).

## RESULTS

Prior to our study, S. aureus was cultured for a mean persistence of 15.6 years (10 to 21 years) from the study patients (Table 1). Three patients were female. The median age was 27.9 years (range, 19 to 45 years). Eight patients carried the homozygous F508del cystic fibrosis transmembrane conductance regulator (CFTR) genotype; six were either heterozygous or carried other mutations. Six patients were chronically coinfected by P. aeruginosa and five were not coinfected, while in three patients, a new coinfection during the study period occurred (Table 2). Fifty-eight visits of 14 study patients were observed (mean, 4.1 visits) including 35 routine visits and 22 visits during exacerbation (information about the clinical condition of one patient from one visit is missing). At 38 out of 58 visits, patients were treated with antibiotics.

**TABLE 1:** CF patients’ demographics and clinical parameters

Patient	CF genotype^*a*^	Sex^*b*^	Age^*c*^ (yr)	Mean lung function FEV_1_%pred^*d*^	S. aureus persistence (yr)	No. of visits	No. of visits with exacerbations^*e*^	Mean S100 A8/A9 (ng/ml)^*f*^	Mean CRP (mg/dl)^*f*^	Mean IL-6 (pg/ml)^*f*^
1	F508del hetero	M	20	49.9	18	5	4	4,335.3	1.64	5.6
2	F508del homo	M	23	91.9	20	4	2	2,732.9	0.20	4.5
3	F508del homo	F	45	35.8	17	4	1	6,509.4	1.43	13.4
4	Other	M	19	78.0	19	4	1	1,066.4	0.28	1.6
5	Other	M	22	78.7	21	4	2	254.3	0.04	1.46
6	F508del homo	M	24	50.3	11	4	1	3,540.3	1.75	14.4
7	F508del hetero	F	40	75.4	18	4	1	1,622.2	0.54	5.0
8	F508del homo	M	18	66.9	14	3	0	2,240.4	0.09	3.8
9	F508del homo	M	29	61.1	10	5	2	8,469.9	1.54	14.0
10	F508del homo	F	16	56.3	15	4	1	3,782.0	0.52	5.6
11	F508del homo	M	23	89.9	15	5	1	3,145.0	0.22	3.9
12	other	M	37	29.6	14	5	4	4,828.1	2.51	20.8
13	F508del hetero	M	39	82.9	12	4	0	2,296.0	0.32	6.0
14	F508del homo	M	35	53.6	15	3	2	4,183.1	0.32	5.5

a CFTR genotype of patients. F508del hetero, F508del heterozygous; F508del homo, F508del homozygous; Other, other mutations.

b M, male; F, female.

c Age at the beginning of the study.

d Mean lung function was calculated as a mean of the lung function data available throughout the study period.

e Visits with exacerbation according to the Fuchs criteria for exacerbation (30).

f Mean values were calculated as a mean of all data available throughout the study period.

**TABLE 2:** S. aureus characteristics and coinfection with P. aeruginosa

Patient	No. of S. aureus isolates^*a*^	Dominant *spa* type^*b*^	*spa* type	Related *spa* type^*c*^	PT^*d*^	MDRA^*e*^				SDI^*f*^		Coinfection with P. aeruginosa^*g*^
						*spa* type	PT	Biofilm formation	Nuclease activity	*spa* type	PT	
1	200	t091	2	0	2.4	0.02	0.35	0.02	0.01	0.08	0.54	New (no PA 3y)
2	160	t1577	2	0	4.5	0.03	0.28	0.00	0.01	0.10	0.58	No PA (4y)
3	160	t008	4	4	4	0.07	0.15	0.00	0.02	0.22	0.73	Chronic (17y)
4	160	t5430	6	4	4	0.11	0.28	0.00	0.04	0.40	0.72	No PA (5y)
5	160	t021	1	0	2.75	0.00	0.21	0.00	0.00	0.00	0.46	Chronic (6y)
6	160	t166	1	0	1.75	0.00	0.07	0.00	0.10	0.00	0.29	No PA (3y)
7	160	t206	6	3	4	0.10	0.13	0.08	0.08	0.48	0.64	No PA (5y)
8	120	t034	1	0	1.6		0.05	0.06	0.05	0.00	0.19	No PA (4Y)
9	200	t002	4	3	3.8	0.02	0.53	0.17	0.12	0.05	0.74	Chronic (6y)
10	160	t617	4	4	5	0.02	0.35	0.03	0.03	0.10	0.87	New (no PA 4y)
11	200	t091	2	2	4.8	0.02	0.33	0.09	0.02	0.03	0.74	New (no PA 4y)
12	200	t011	5	4	4	0.19	0.50	0.22	0.29	0.49	0.69	Chronic (15y)
13	159	t003	13	13	8.25	0.11	0.24	0.07	0.11	0.39	0.87	Chronic (8y)
14	120	t067	5	2	2	0.05	0.04	0.02	0.00	0.27	0.30	Chronic (15y)

a Number of collected S. aureus isolates from the respective patient from sputum cultures during the study, which have been further analyzed.

b Dominant *spa* type, which is present at all visits in a high percentage of isolates.

c Number of *spa* types related to the dominant *spa* type, in which mutations were observed resulting in a different *spa* type with close relation to the dominant *spa* type.

d Mean number of phenotypes (PTs) observed in sputa.

e Mean daily rate of alteration as described by Roden et al. (68).

f Simpson’s diversity index.

g PA, P. aeruginosa; y, years.

During every visit, 40 S. aureus isolates were picked from Columbia blood or/and CAP agar to include all different phenotypes (PTs) (*n* = 2,319 isolates, one isolate had to be excluded due to incorrect species identification). A total of 556 isolates (24%) cultured from 12 patients were SCVs, 1,840 isolates (79%) from all patients were positive for hemolysis, 331 isolates (14%) from 7 patients were positive for β-toxin, while 501 isolates (22%) from 10 patients were mucoid.

**FIG 1:**
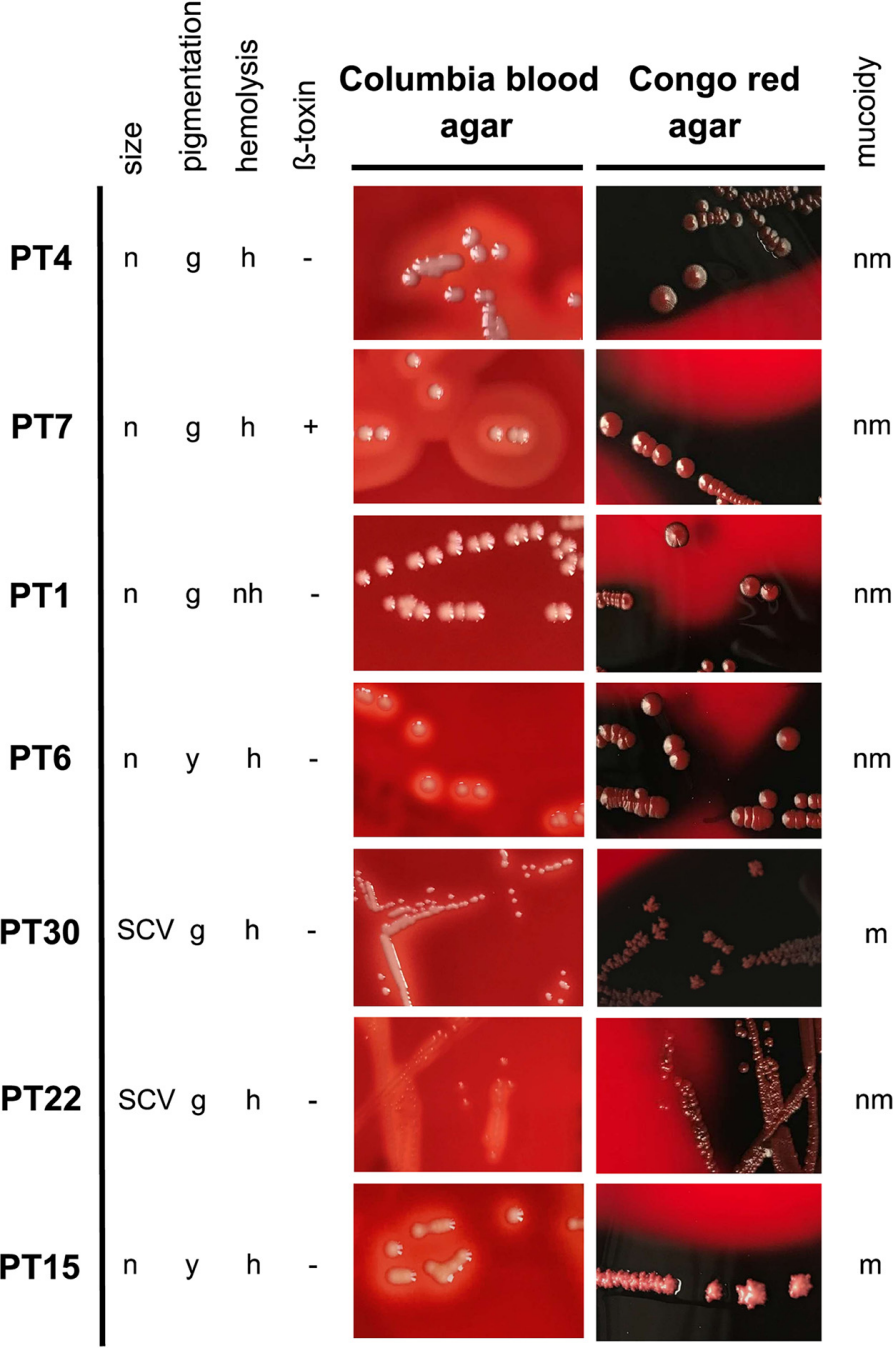
Pictures of the seven most abundant phenotypes (PTs). S. aureus strains were cultured on Columbia blood agar and Congo red agar (CRA) plates and are characterized by the following parameters: size, mucoidy, hemolysis, β-toxin, and pigmentation. CRA was used to facilitate the discrimination of mucoid isolates. Special characteristics for mucoid isolates on CRA are a pyramidal-shaped morphology with rough wrinkled edges and a dry crystalline consistency (PT30 and -15), while nonmucoid isolates appear as flat and smooth colonies with a more moist consistency (PT4, -7, -1, -6, and -22). PT4 is normal (n), nonmucoid (nm), hemolytic (h), β-toxin negative (-), and gray (g). PT7 is normal, nonmucoid, hemolytic, β-toxin positive (+), and gray. PT1 is normal, nonmucoid, nonhemolytic (nh), β-toxin negative, and gray. PT6 is normal, nonmucoid, hemolytic, β-toxin negative, and yellow (y). PT30 is an SCV, mucoid (m), hemolytic, β-toxin negative, and gray. PT22 is an SCV, nonmucoid, hemolytic, β-toxin negative, and gray. PT15 is normal, mucoid, hemolytic, β-toxin negative, and yellow.

PTs were assigned according to the different parameters, which resulted in 32 different PTs (see Table S1 in the supplemental material). Seven PTs, which included 77% of all isolates, were the most abundant with more than 100 isolates belonging to each PT (Fig. 1 and Table 3). Phenotype 4 (PT4) was the most abundant, which was observed in 868 isolates (37%) cultured from 12 patients. Isolates of PT4 were characterized by normal, nonmucoid, hemolytic, β-toxin-negative, and gray colonies (Fig. 1). However, SCVs (PT30 and PT22), mucoid (PT30 and PT15), nonhemolytic (PT1) and β-toxin-positive (PT7) PTs were also observed within the most abundant PTs (Fig. 1 and Table 3).

**TABLE 3:** The most prevalent seven S. aureus phenotypes

Phenotype	Size^*a*^	Mucoidy^*b*^	Hemolysis^*c*^	β-toxin^*d*^	Pigmentation^*e*^	No. of isolates^*f*^	%^*g*^	No. of patients	No. of *spa* types	Nuclease > 100% (% of isolates)	Biofilm > 10% (% of isolates)
4	1	1	2	1	1	868	37	12	27	12	6
7	1	1	2	2	1	231	10	5	7	48	14
1	1	1	1	1	1	227	10	11	14	7	1
6	1	1	2	1	3	136	6	8	11	3	0
30	2	2	2	1	1	116	5	7	11	70	64
22	2	1	2	1	1	114	5	9	15	25	25
15	1	2	2	1	3	105	5	4	6	1	97

a Size, according to the size of the colonies on Columbia blood agar: 1 = normal; 2 = SCVs.

b Mucoidy: mucoid growth at least on one of the different agars (Columbia blood agar, Schaedler agar, or Congo red agar): 1 = nonmucoid; 2 = mucoid.

c Hemolysis: 1 = no hemolysis; 2 = hemolysis (as observed on Columbia blood agar).

d β-toxin: 1 = no β-toxin; 2 = β-toxin (as observed on Columbia blood agar after heat/cold lysis; 24 h at 37°C, 24 h at 4°C).

e Pigmentation: 1 = gray, 2 = white, 3 = yellow (as observed on Columbia blood agar).

f All isolates with these special characteristics.

g %, percentage of isolates with these special characteristics.

10.1128/mSphere.00358-21.5 TABLE S1 S. aureus phenotypes during the prospective study. Download Table S1, DOCX file, 0.04 MB. Copyright © 2021 Wieneke et al. 2021 Wieneke et al. https://creativecommons.org/licenses/by/4.0/ This content is distributed under the terms of the Creative Commons Attribution 4.0 International license.

**FIG 2:**
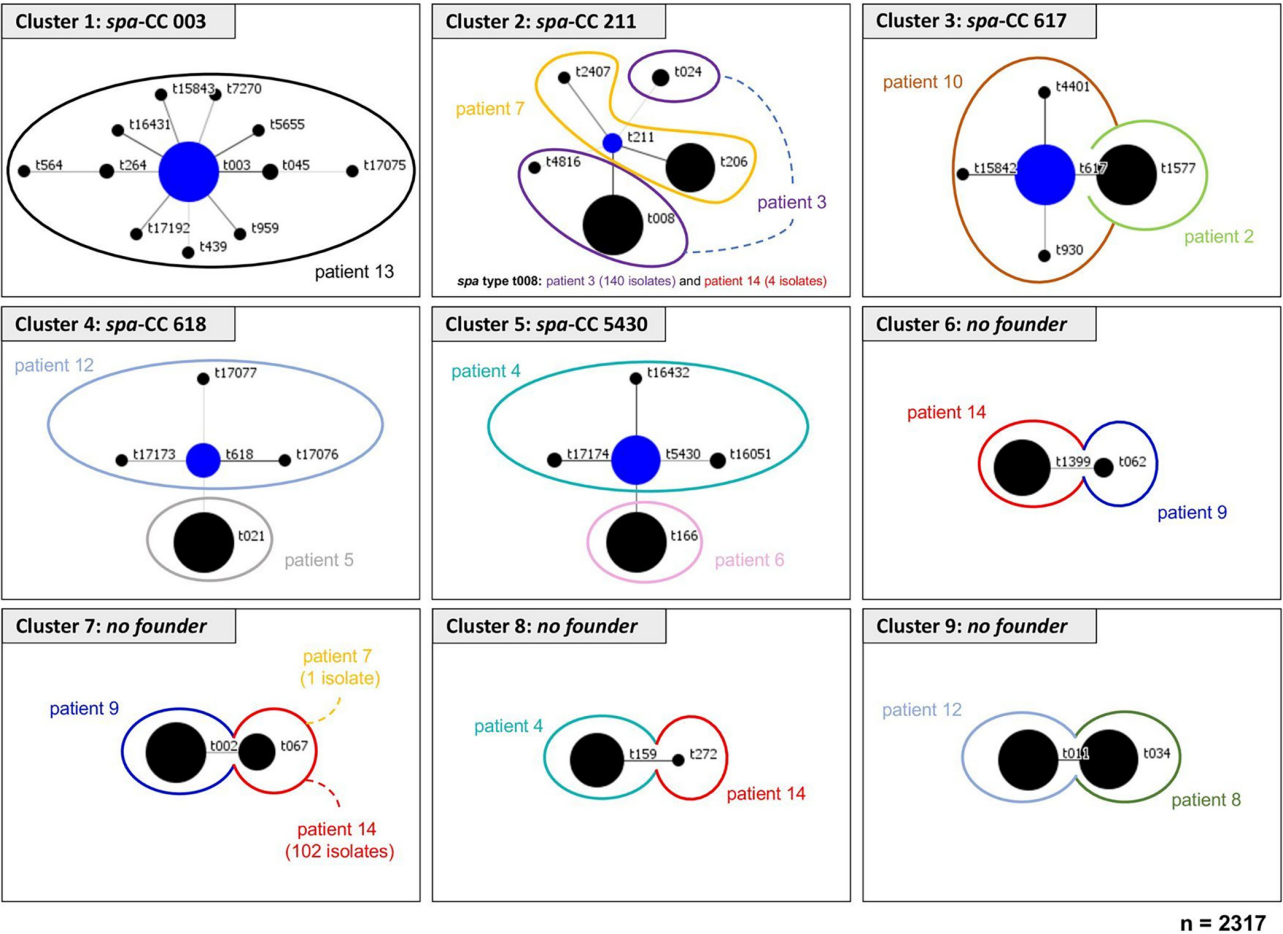
*spa* clonal complexes (*spa*-CCs) of S. aureus isolates in the prospective study. This figure demonstrates the clonal relatedness of all 50 *spa* types of the 2,319 S. aureus isolates from 14 CF patients during the study period (two CF centers). The analysis was performed using the BURP algorithm as implemented in the Ridom StaphType software (BURP, Ridom StaphType software; Ridom GmbH, Würzburg, Germany). The main founder *spa* type is shown in blue in the middle of each cluster. The size of each circle demonstrates the number of isolates with the respective *spa* type. Colored loops surrounding the circles indicate the patient with the respective *spa* type. For CC6 to CC9 isolates, the software could not determine a founder *spa* type, and therefore, the isolates with these respective *spa* types are presented as “no founder.” Eight *spa* types could not be related to other *spa* types and were therefore determined as “singletons.” t005, t084, t091, t267, t1204, t2202, t3127, and t13342 were singletons. *spa* types with less than five repeats were excluded, which resulted in the exclusion of one *spa* type (t463).

The molecular analysis of all S. aureus isolates performed by *spa* typing identified 50 different *spa* types (Table S2) (two isolates were nontypeable). From every patient, one dominant S. aureus *spa* type (clone) was cultured during all visits (Table 2). Only in patients 1 and 11, isolates with the same *spa* type were detected as the dominant clone (t091). For 9 patients, isolates with related *spa* types of the main clone were recovered (patients 3, 4, 7, and 9 to 14 [Tables 2 and 4]), while for the other patients (patients 1, 2, 5, and 6), two to five unrelated *spa* types were present during the study period. To visualize the relatedness of S. aureus isolates, *spa* types of the isolates were grouped into clonal complexes (CC) by BURP analysis, which compares the base sequence of the repeat region of the respective *spa* types, for every patient individually (Table 5) and for all patients (Fig. 2), which resulted in the differentiation of nine *spa* clonal clusters (*spa*-CCs) (Fig. 2), revealing that all patients carried their individual clones. Isolates of patient 13 were the most dynamic ones belonging to cluster 1, which represents 158 of 159 isolates of patient 13. These isolates belonged to *spa*-CC003 with 123 isolates of the founder *spa* type t003, from which isolates with 12 different *spa* types evolved (Tables 5 and 6 and Fig. 2). Most of these isolates were cultured from only one or two visits, indicating that they were not stable (Tables 5 and 6). For the most prevalent *spa* types, 2 to 16 different PTs were observed (Table 6), with a mean number of 8 PTs. In 12 patients, isolates belonging to the same *spa*-CCs, clusters (CC211, CC5430, CC617, cluster 7, and cluster 9) or *spa* type (t091) were observed. In four of the same *spa-*CCs, cluster, or *spa* type, the number of PTs for the individual patients were higher and lower than the mean PT of 8 (Table 6), while only isolates of CC617 and cluster 9 showed higher or lower numbers of PTs (8 and 11 PTs for patients 2 and 10; 2 and 5 PTs for patients 8 and 12; Table 6) indicating that mostly, diversity of S. aureus was related to the individual patient, while only for isolates of CC617 and cluster 9, the diversity of S. aureus might be due to the clonal background. The observation of PTs in *spa* types for all patients is shown in Fig. S1 in the supplemental material.

**TABLE 4:** Mutations within the VNTR region of related *spa* types

Patient	All clones^*a*^	Related clones^*b*^	Nonrelated^*c*^	% of related clones^*d*^	No. of isolates^*e*^	*spa* type^*f*^	VNTR region^*g*^	Mutation(s)^*h*^	Repeat^*i*^	Nucleotide sequence of the repeat region^*j*^
3	4	4	0	100	140	**t008**	11-**19**-12-21-17-**34**-24-34-22-25			
					1	t13342	11-19-12-21-17-34-24-34-**17-34**	several		
					1	t4816	11-19-12-21-17-24-34-22-25	del		
					18	t024	11-12-21-17-34-24-34-22-25	del		
4	6	4	2	67	123	**t5430**	04-**44**-33-31-12-16-34-16-34-16-12-25-22-22-34			
					14	t16051	04-33-31-12-16-34-16-34-16-12-25-22-22-34	del		
					10	t17174	04-44-33-31-12-16-34-16-34-16-12-**16-12**-25-22-22-34	dupl		
					1	t16432	04-44-33-**33**-31-12-16-34-16-34-16-12-25-22-22-34	dupl		
7	6	3	3	50	112	t206	11-**19**-12-12-**12**-21-17-34-24-34-22-25			
					26	**t211**	11-19-12-12-21-17-34-24-34-22-25	del		
					1	t2407	11-12-12-12-21-17-34-24-34-22-25	del		
9	4	3	1	75	195	t002	26-23-17-**34-17-20-17**-12-17-16			
					3	t062	26-23-17-12-17-16	del		
					1	t3127	26-**30**-17-34-17-20-17-**02**-17-**20-17-12-17-16**	several		
10	4	4	0	100	152	**t617**	15-**21**-16-02-24-**24**			
					3	t15842	15-21-16-02-24-24-**02-24-24**	dupl		
					4	t4401	15-21-16-02-24	del		
					1	t930	15-16-02-24-24	del		
11	2	2	0	100	197	t091	07-23-21-**17-34-12**-23-02-12-23			
					3	t1204	07-23-21-23-02-12-23	del		
12	5	4	1	80	74	**t618**	15-12-16-02-16-**02-25**-17-17-17-24			
					1	t17076	15-12-16-02-16-02-**24**-17-17-17-24	pm	r24	AAAGAAGATGGCAACAAGCCTGGT
					1	t17077	15-12-16-02-16-**17**-25-17-17-17-24	several		
					1	t17173	15-12-16-02-16-**17-17-17-24-17-24**	del and dupl		
13	13	13	0	100	123	**t003**	26-17-**20**-17-**12**-**17**-**17**-16			
					11	t045	26-17-20-17-12-17-16	del		
					1	t15843	26-17-20-**211**-12-17-16	pm	r211	AAAGAAGACGGCAACAAGCC**C**GGT
					9	t264	26-17-20-17-17-17-16	del		
					2	t439	26-17-20-17-17-16	del		
					1	t5655	26-17-20-17-12-17-17-**17**	pm	r17	AAAGAAGACGGCAACAA**G**CCTGGT
					2	t16431	26-17-20-**16**-12-17-17-16	pm	r16	AAAGAAGACGGCAACAA**A**CCTGGT
					2	t17075	26-17-12-17-12-12-17-17	several		
					1	t463	26-17-17-16	del		
					2	t564	26-17-17-17-16	del		
					2	t959	26-17-**02**-17-12-17-17-16	pm	r02	AAAGAAGACAACAA**A**AAACCTGGC
					1	t7270	26-17-**22**-17-12-17-17-16	pm	r22	AAAGAAGAC**GG**CAACAA**G**CCTGGC
					1	t17192	**750**-17-20-17-12-17-17-**17**	pm	r750	G**G**GGAAGACAACAAAAAACCTGGT
									r17	AAAGAAGACGGCAACAA**G**CCTGGT
14	5	2	3	40	102	t067	26-23-17-**34-17-20-17**-12-17			
					12	t1399	26-23-17-12-17	del		

a All clones, all different *spa* types isolated from the airways of this patient.

b Related clones, number of *spa* types, which evolved most likely due to mutational events in the variable number of tandem repeats (VNTR) of *spa* during persistence.

c Nonrelated clones, number of additional clones with *spa* types characterized by a nonrelated repeat region of *spa*.

d % of related clones, percentage of isolates with related *spa* types.

e Number of isolates with the respective *spa* type.

f *spa* type, the different *spa* types of patients with related *spa* types; ancestor strains are indicated in bold type.

g VNTR region, the sequence of the repeats within the VNTR region. The mutated repeats are indicated in bold type in the ancestor strain.

h Mutations, the mutational event that caused the changed repeat succession: del, deletion; pm, point mutation; dupl, duplication; several, several events (several different mutations occurred in the VNTR region, including pm, del, and dupl).

i Repeat, the number of repeat, which shows a point mutation, which leads to a different repeat number and to a different *spa* type.

j Nucleotide sequence, the changed nucleotide sequence of the repeat caused by one point mutation.

**TABLE 5:**
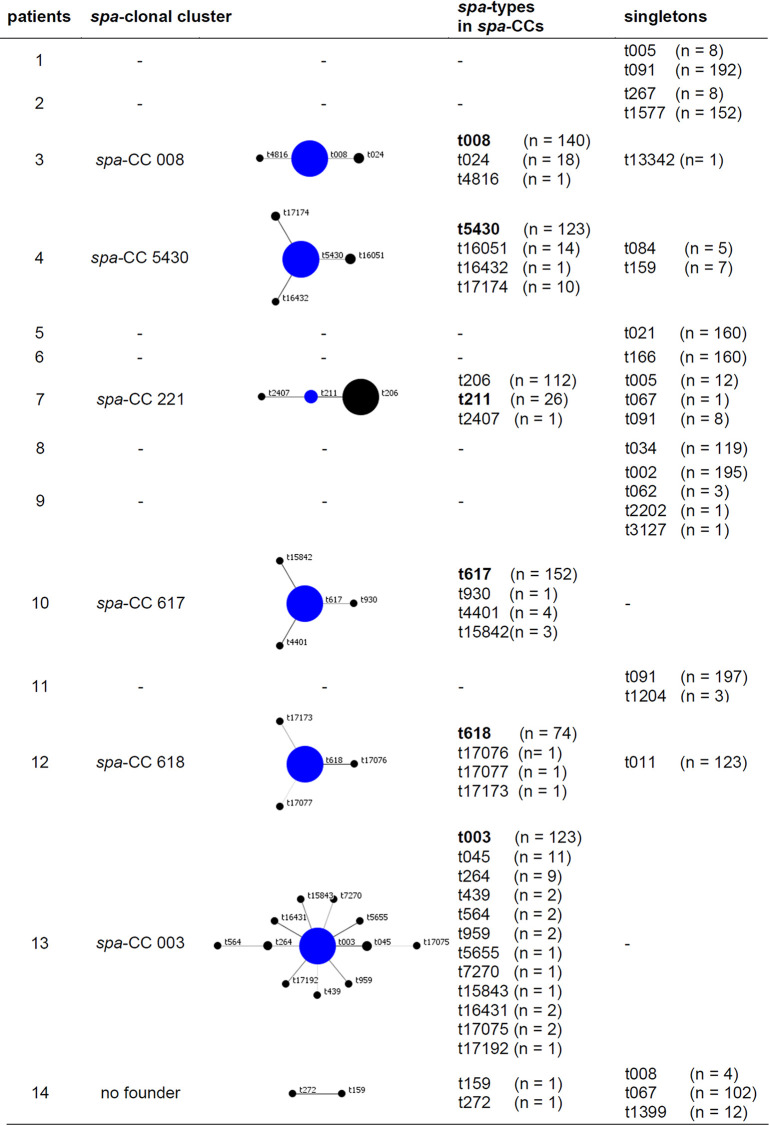
BURP analysis for S. aureus isolates of individual patients^*a*^

a This table demonstrates the relatedness of *spa* types within each CF patient during the study period. The analysis was performed using the BURP algorithm as implemented in the Ridom StaphType software (BURP, Ridom StaphType software; Ridom GmbH, Würzburg, Germany). The main founder *spa* type is shown in blue in the middle of each cluster and is shown in bold type in the table. The size of each circle demonstrates the number of isolates with the respective *spa* type. For patient 14, the software could not determine a founder *spa* type, and therefore, the isolates with these respective *spa* types are presented as “no founder.” *spa* types that could not be related to other *spa* types were determined as “singletons.” *spa* types with less than five repeats were excluded, which resulted in the exclusion of one *spa* type (t463 from patient 13).

**TABLE 6:** PTs of the most prevalent *spa* types

*spa*-CC^*a*^	*spa* type	No. of PTs^*b*^	No. of isolates^*c*^	Patient
CC003	t003	16	123	13
CC211	t008	10	140	3
CC211	t206	5	112	7
CC5430	t5430	9	123	4
CC5430	t166	3	160	6
CC617	t1577	8	152	2
CC617	t617	11	152	10
CC618	t021	6	160	5
No founder – cluster 7	t002	11	195	9
No founder – cluster 7	t067	3	102	14
No founder – cluster 9	t034	2	119	8
No founder – cluster 9	t011	5	123	12
Singleton	t091	5	192	1
Singleton	t091	9	197	11

a BURP analysis clustered *spa* types into clonal clusters (*spa*-CCs) depending on the repeat succession within the repeat region of protein A. “No founder” indicates that the repeat succession of *spa* types is very similar, but a founder could not be determined. However, the *spa* types belong to specific clusters. “Singleton” indicates that in the studied S. aureus isolate collection, no isolates with related sequences within the repeat region were found.

b PTs, the number of different PTs within the *spa* type of the individual patients.

c Number of isolates of the respective *spa* type for the individual patient.

10.1128/mSphere.00358-21.1 FIG S1 PTs for *spa* types for all patients. For every patient, the different *spa* types and the determined PTs are indicated. A summarizing table beneath the graph reveals the number of isolates for every *spa* type and PT. Download FIG S1, PDF file, 0.5 MB. Copyright © 2021 Wieneke et al. 2021 Wieneke et al. https://creativecommons.org/licenses/by/4.0/ This content is distributed under the terms of the Creative Commons Attribution 4.0 International license.

10.1128/mSphere.00358-21.2 FIG S2 Heatmaps of all patients (patients 2 to 8 and 10 to 14). The heatmaps illustrate all data for 40 S. aureus isolates collected during every visit in terms of *spa* type (dominant *spa* type/related to the dominant *spa* type/not related), PT (mucoidy, hemolysis, β-toxin, SCV phenotype [all positive/negative], pigment [gray/white/yellow] as well as biofilm formation [low < 10%/high > 10% of the positive control] and nuclease activity [low < 100%/high > 100% of the positive control]). Below the heatmap, data of all visits are reported for months from first visit, exacerbation, P. aeruginosa coinfection, density of S. aureus and P. aeruginosa in CFU/ml. On the right, data for S100A8/9, CRP, and IL-6 and lung function are given for every visit in a table and illustrated as a graph with visits of exacerbation marked with a red arrow. Download FIG S2, PDF file, 0.7 MB. Copyright © 2021 Wieneke et al. 2021 Wieneke et al. https://creativecommons.org/licenses/by/4.0/ This content is distributed under the terms of the Creative Commons Attribution 4.0 International license.

**FIG 3:**
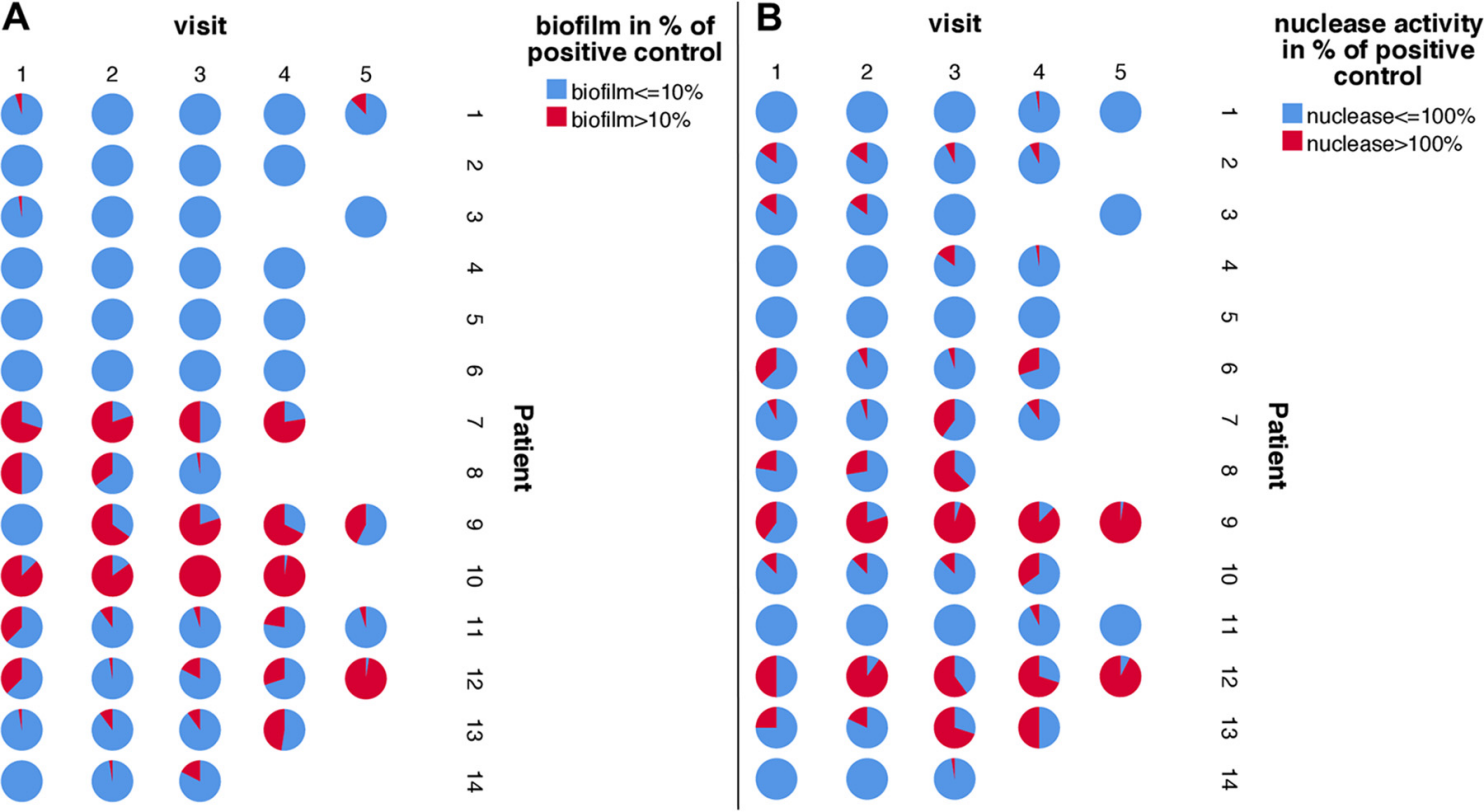
Biofilm formation and nuclease activity for all isolates and for all patients. This figure shows the amount of biofilm formation (A) and nuclease activity (B) for all S. aureus isolates from all patients at all visits. (A) We arbitrarily determined the categories low and high biofilm formation if the isolates formed less or more than 10% of the biofilm of the positive control (RP62A, a coagulase-negative Staphylococcus that produces large amounts of biofilm compared to S. aureus). In patients 2 to 6, no high biofilm-forming isolates have been observed, while an increasing biofilm formation of isolates was visible for patients 1, 12, 13, and 14. In patients 8 and 11, a decrease of biofilm formation was determined, while from patients 7, 9, and 10, a large percentage of high biofilm-forming isolates over all visits were detected. (B) For the nuclease activity, we distinguished groups with low and high nuclease activity if less or more than 100% of the nuclease activity of the positive control (AH1263) were measured. Half of the patients (patients 6 to 10, 12, and 13) carried more than 92% of isolates with high nuclease activity, while in the other patients, only small numbers of isolates with high nuclease activity were detected. In patient 5 only, no isolates with high nuclease activity were recovered.

Since biofilm formation represents an important virulence factor for S. aureus not only to escape host response and antibiotic therapy but also to confer protection against coinfecting pathogens (28), we assessed the quantity of biofilm formation for all S. aureus isolates using a static biofilm assay. Most isolates (*n* = 1,773, 76.5%) were only minor biofilm-forming strains, while 546 isolates (23.5%) produced large amounts of biofilm (Fig. 3A). In summary, for five patients, no biofilm-positive isolates were observed (patients 2 to 6 [Fig. 3A]). For four patients, an increase of biofilm-forming isolates (patients 1 and 12 to 14) was observed. For two patients, a decrease in biofilm-forming isolates (patients 8 and 11) was seen, and for three patients, a large percentage of high biofilm-forming isolates (patients 7, 9, and 10) was seen, respectively.

Nuclease represents another important virulence factor of S. aureus with different functions. While high nuclease activity facilitates escape from NETs (18, 19), low nuclease activity allows S. aureus to remain in biofilm growth (29). Using a fluorescence resonance energy transfer (FRET) assay, we measured nuclease activity for all isolates, revealing 1,176 isolates (76.6%) expressing nuclease activity up to 100% of the positive control, whereas 543 isolates (23.4%) expressed nuclease activity higher than 100% of the positive control (Fig. 3B). For half of the patients, only small numbers of isolates with high nuclease activity were detected (patients 1 to 5, 11, and 14), while for the other patients, a changing percentage of high nuclease active isolates were observed (Fig. 3B).

**FIG 4:**
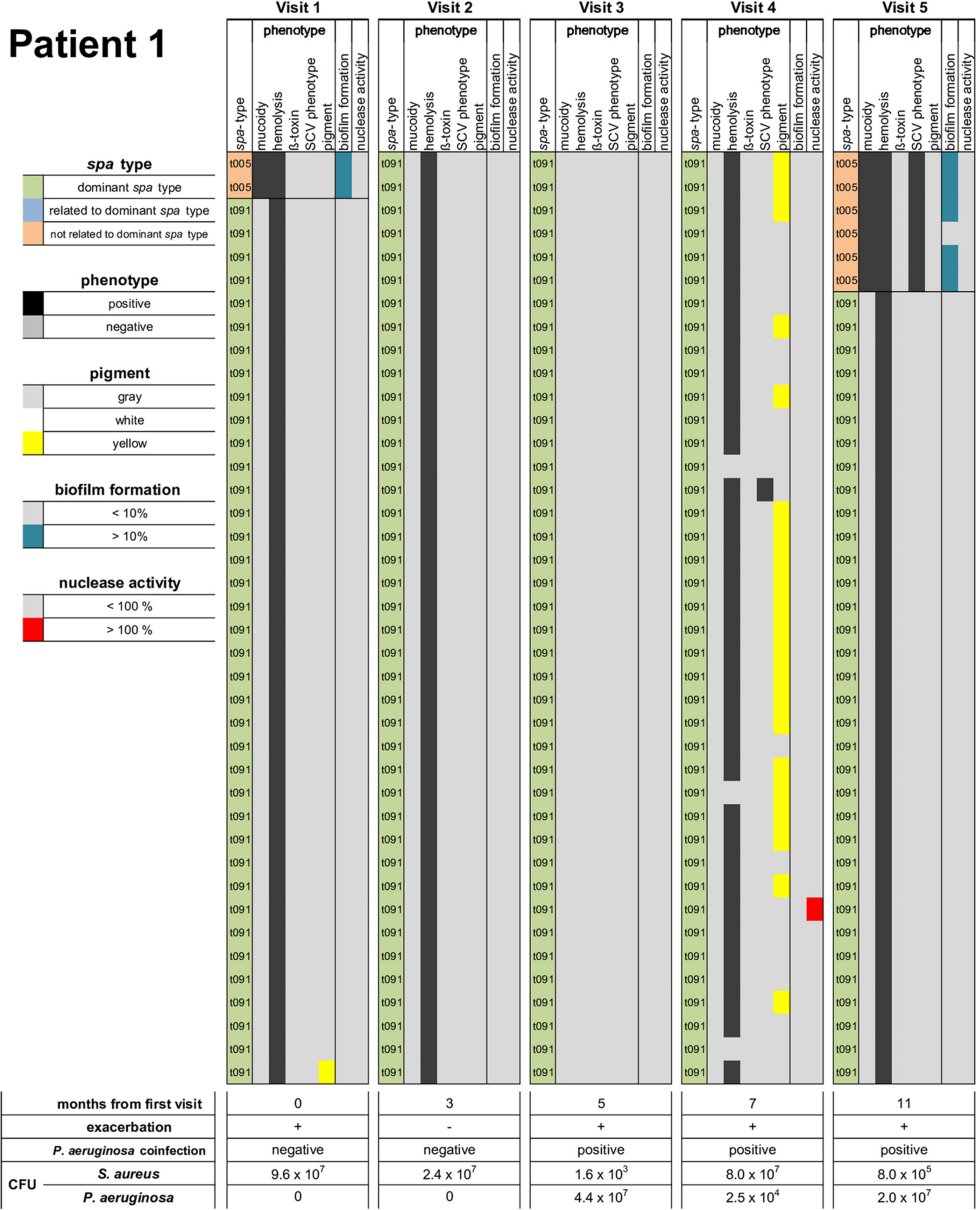

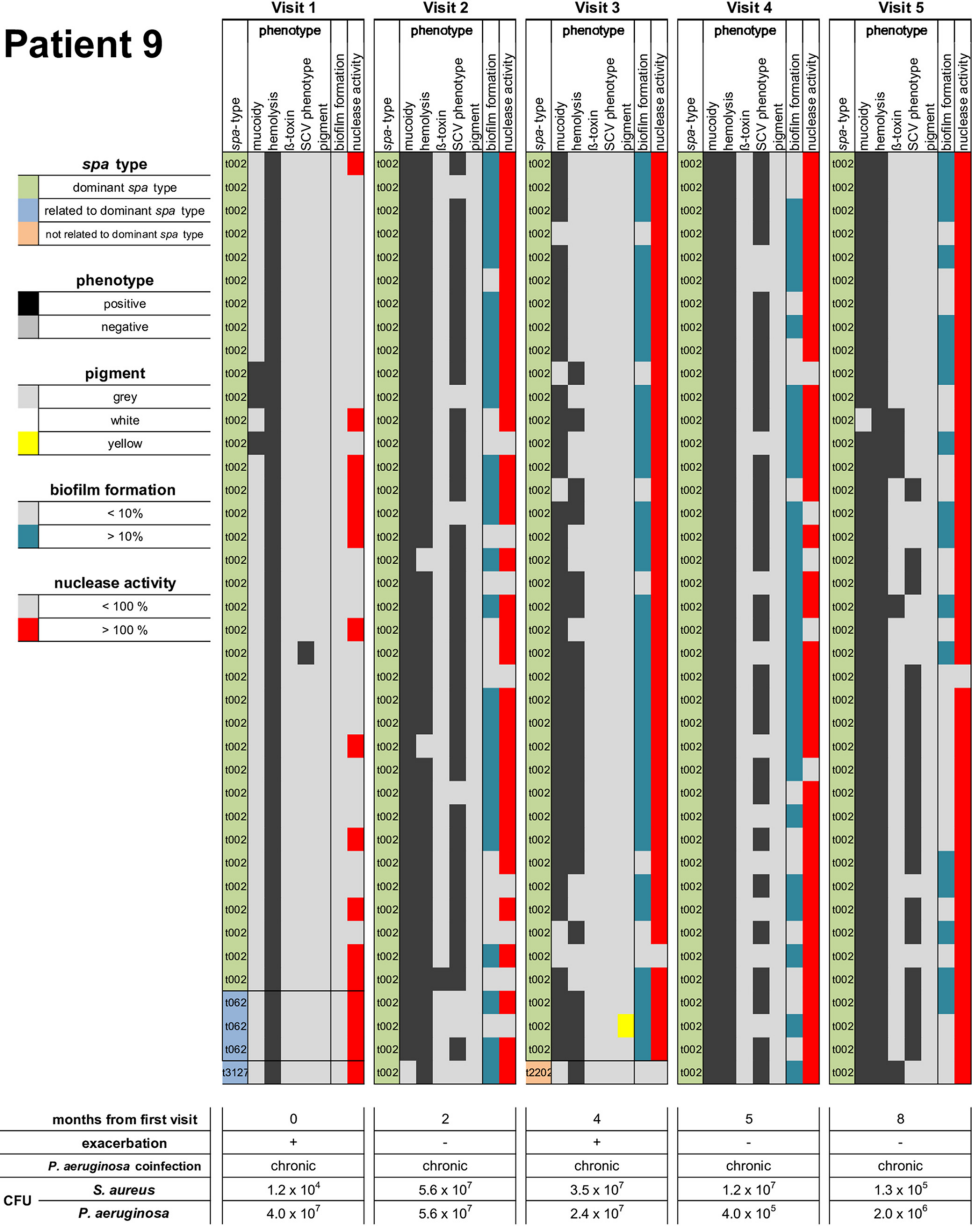

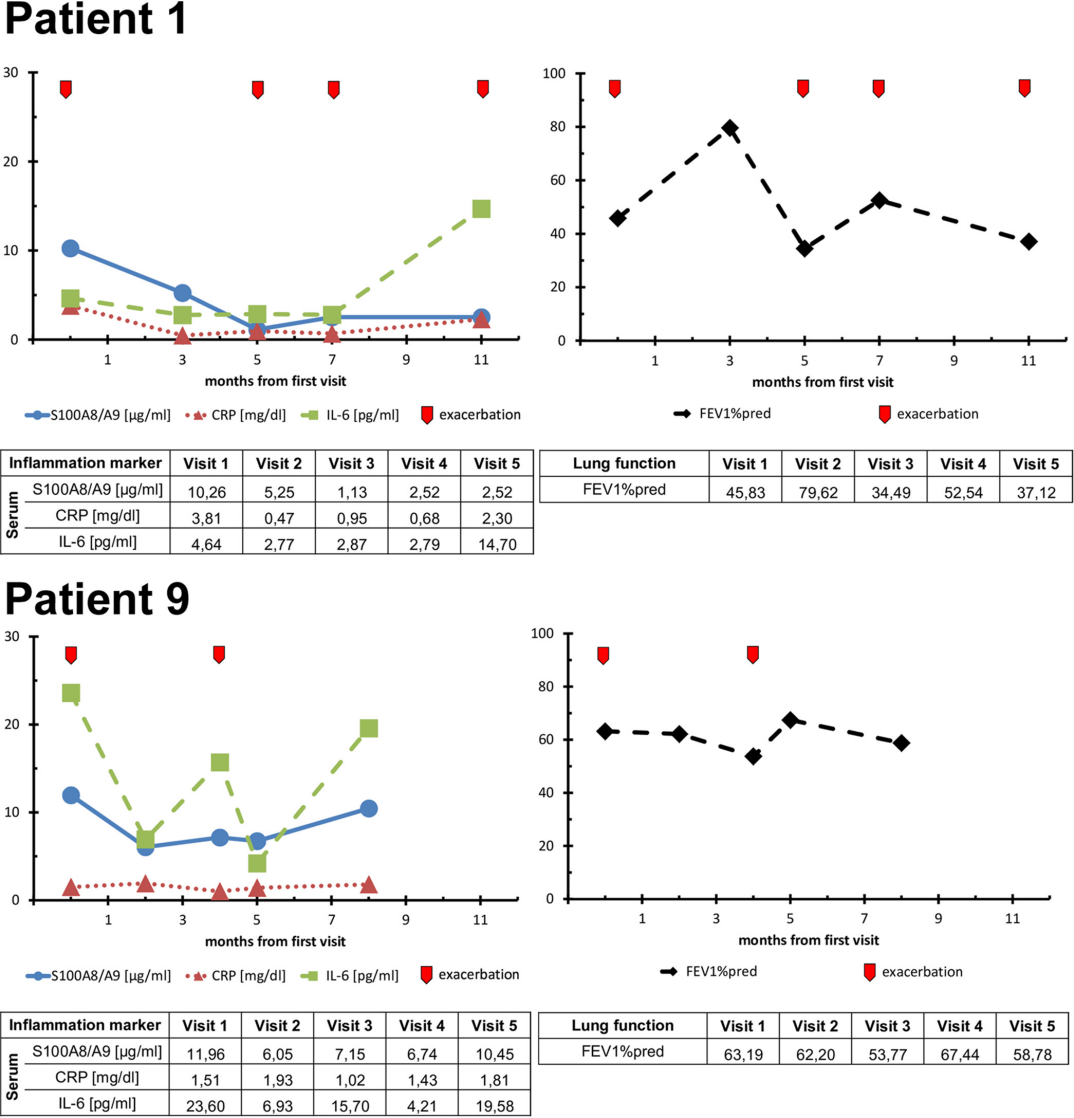
Heatmaps of S. aureus isolates of patients 1 and 9 with inflammatory and lung function data of every visit. The heatmaps illustrate all data for 40 S. aureus isolates collected during every visit in terms of *spa* type (dominant *spa* type/related to the dominant *spa* type/not related), phenotype (PT) (mucoidy, hemolysis, β-toxin, SCV phenotype [all positive/negative], pigment [gray/white/yellow] as well as biofilm formation [low < 10% / high > 10% of the positive control] and nuclease activity [low < 100% / high > 100% of the positive control]). Below the heatmap, data of all visits are reported for months from first visit, exacerbation, P. aeruginosa coinfection, SDI for *spa* type and PTs. Data for S100A8/9, CRP, IL-6, and FEV_1_%pred are given for every visit in a table and illustrated as a graph with visits of exacerbation marked with a red arrow.

**FIG 5:**
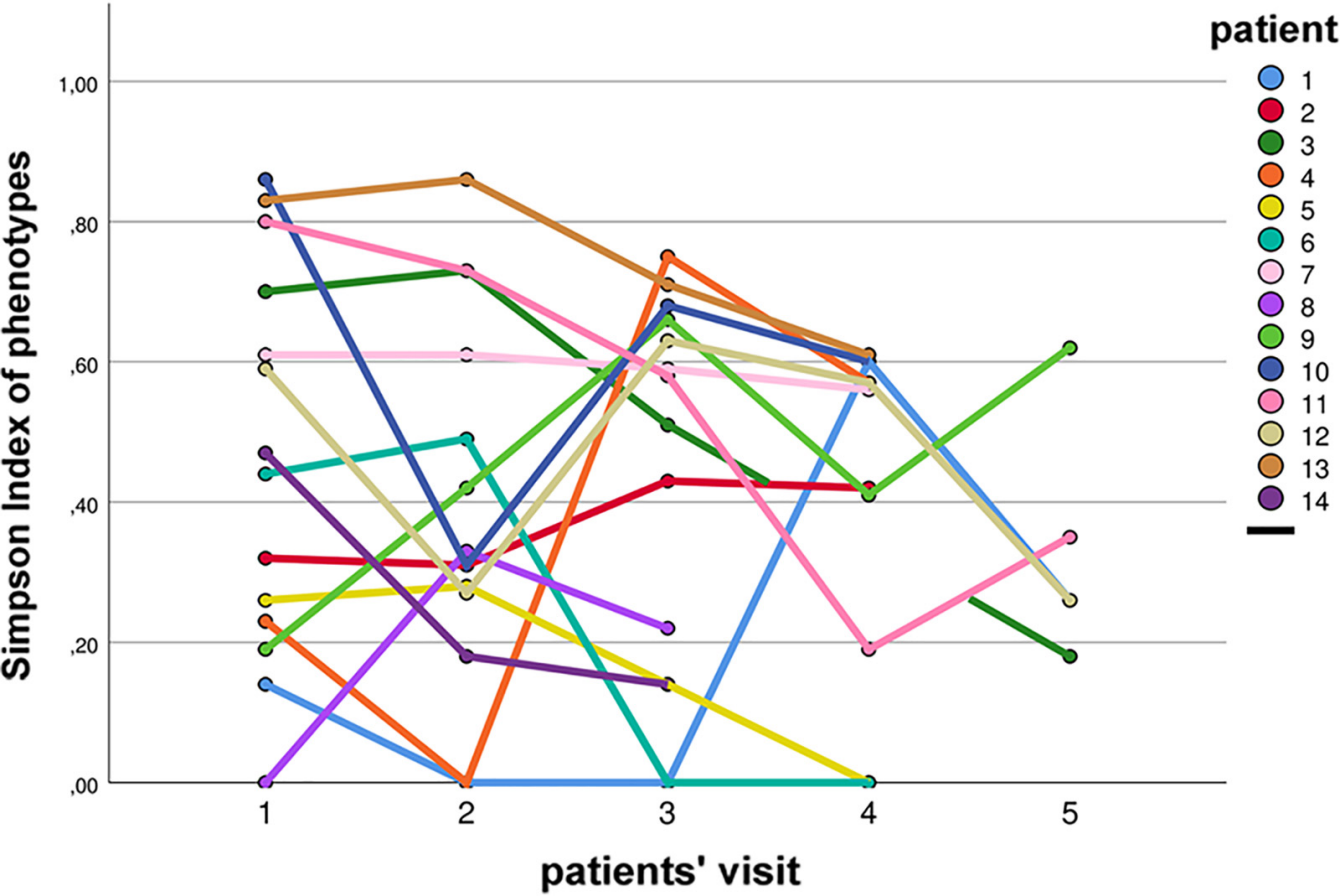
Simpson diversity index (SDI) of phenotypes (PTs). The SDIs of PTs at every patients’ visit are shown to present the dynamics of diversity.

The diversity of isolates within single sputum specimens and in sputa from different visits is presented as heatmaps as exemplified for patients 1 and 9 in Fig. 4 and for all patients in Fig. S2. Also, the dynamics of PTs during the study are illustrated in Fig. S3. To determine the diversity of S. aureus, the Simpson’s diversity indices (SDIs) for each sputum specimen and for all sputa of individual patients in regard to PTs and *spa* types were calculated (Table 2). The SDIs of *spa* types from the patients ranged from 0.00 (patients 5 and 6) to 0.49 (patient 12), and for PTs, the SDIs ranged from 0.19 (patient 8) to 0.87 (patient 13). SDI levels were mostly fluctuant for patients from one visit to the other (Fig. 5). There were only two patients with rather stable SDI levels (patients 2 and 7).

10.1128/mSphere.00358-21.3 FIG S3 Phenotype (PT) diversity and dynamics for all patients. PTs are shown with individual colors for every PT. For every patient, PTs at every visit are presented to illustrate the diversity and dynamics of PTs during the study period. Download FIG S3, PDF file, 0.2 MB. Copyright © 2021 Wieneke et al. 2021 Wieneke et al. https://creativecommons.org/licenses/by/4.0/ This content is distributed under the terms of the Creative Commons Attribution 4.0 International license.

For further analysis, we associated the observed S. aureus characteristics with each other and with clinical parameters using the general estimating equations (GEE) model. Of all investigated S. aureus traits, only biofilm formation showed an association with clinical parameters. Unexpectedly, if isolates were high biofilm formers, fewer exacerbations in patients were observed (*P* = 0.002). Conversely, exacerbations had a negative impact on biofilm formation (*P* < 0.0001). There was no significant impact of antibiotic treatment on PTs possibly due to the use of different antibiotics. However, for any given antibiotic therapy, an impact on phenotypical characteristics was shown for hemolysis and SCVs. In visits where patients received antibiotics effective on S. aureus, the number of hemolytic isolates decreased (*P* = 0.004) and the number of SCVs increased (*P* = 0.010). Also, there was no significant impact of antibiotic therapy on the patients’ clinical status (lung function, inflammatory parameters, and exacerbations).

Even though SCVs are usually metabolically less active compared to normal S. aureus (30, 31), in this study, the SCV phenotype was associated with high nuclease activity (*P* = 0.045). In line with earlier data (16, 17), mucoidy was associated with high biofilm formation (*P* < 0.001), while biofilm formation was also associated with the mucoid PTs (*P* = 0.003). However, not all mucoid PTs formed biofilm, and not all biofilm formers were mucoid.

Concerning associations of SDI and PT characteristics, the SDI was positively associated with mucoid (*P* = 0.006) and SCV (*P* = 0.047) PTs, indicating that in sputa with high diversity, mucoid and SCVs contributed to this effect.

**FIG 6:**
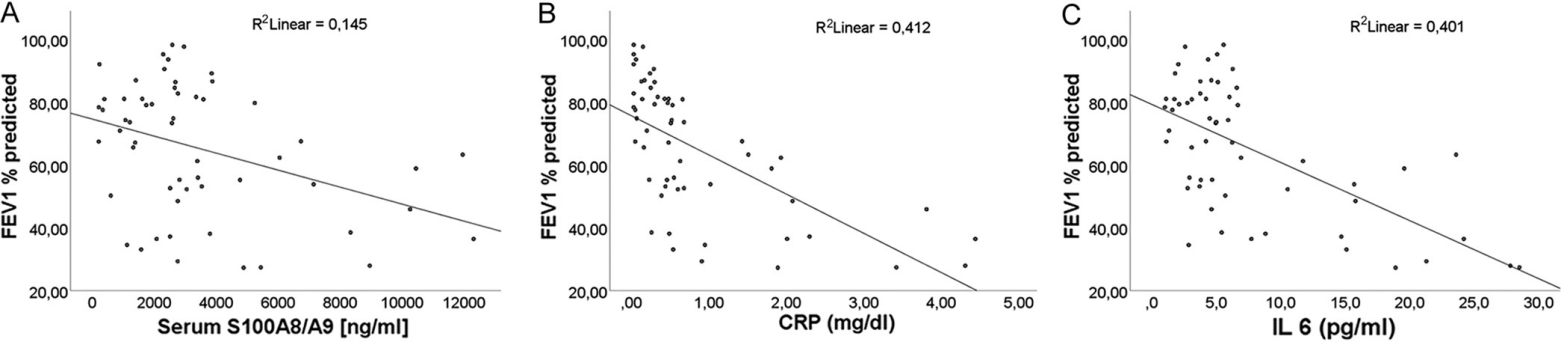
Lung function of patients correlated with inflammatory markers S100 A8/A9, CRP, and IL-6. Data of the patients’ lung function (FEV_1_% predicted) were collected at every visit and correlated with the following inflammatory markers from patients’ serum S100A8/A9 (A), CRP (B), and IL-6 (C). Applying GEE, the linear fit line shows a negative correlation for all inflammatory parameters with the FEV_1_%pred revealing that decreased lung function is associated with higher inflammatory parameters (*P* < 0.0001).

During our study, we also analyzed inflammatory parameters in sera from each patient at every visit as a potential marker for disease severity (32) as shown in Fig. 4 for patients 1 and 9 for each visit and as a mean of data from all visits for all patients in Table 1 (all data for visits and patients in Fig. S2). Consistent with other studies, the level of IL-6 was inversely associated with lung function parameters such as forced expiratory volume in 1 s (FEV_1_%pred) (*P* = 0.001) (32, 33). Also, FEV_1_%pred was inversely associated with all investigated inflammatory parameters (S100A8/9, IL-6, and CRP; for all, *P* < 0.0001) (Fig. 6). In addition, all inflammatory parameters were positively associated with exacerbations (for S100A8/9, *P* = 0.007; for IL-6 and CRP, *P* < 0.001), and exacerbations were positively associated with S100A8/9 (*P* = 0.001) and IL-6 and CRP (*P* < 0.05 for both).

**FIG 7:**
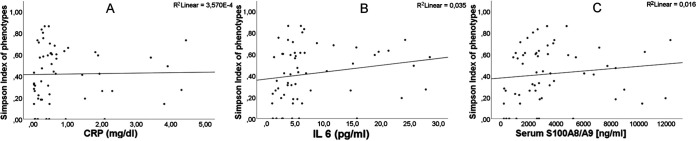
Association of inflammatory markers S100 A8/A9, CRP, and IL-6 and SDI of phenotypes (PTs). Inflammatory markers from patients’ sera for CRP (A), IL-6 (B), and S100A8/A9 (C), which were determined at every visit, were correlated with the SDI of PTs by GEE. The most sensitive parameter was IL-6, for which a positive association with the SDI of PTs (*P* = 0.001) was observed, while there was no clear trend for S100A8/9 and CRP.

Of the tested inflammatory parameters, IL-6 was the most sensitive with a positive association with the SDIs of PTs (*P* = 0.001) (Fig. 7), indicating that within inflamed airways, a high diversity of S. aureus isolates occurred. Since the SDIs of PTs were also positively associated with IL-6 (*P* = 0.024), it can be speculated that diverse S. aureus isolates populating the airways might also trigger inflammation within this niche.

IL-6 and CRP were positively associated with high nuclease activity of isolates (*P* < 0.001 and *P* = 0.049, respectively), while S100A8/9 showed a trend (*P* = 0.051) indicating that in inflamed airways, S. aureus isolates with high nuclease are selected, thereby presumably facilitating escape from NETs (18, 19).

**FIG 8:**
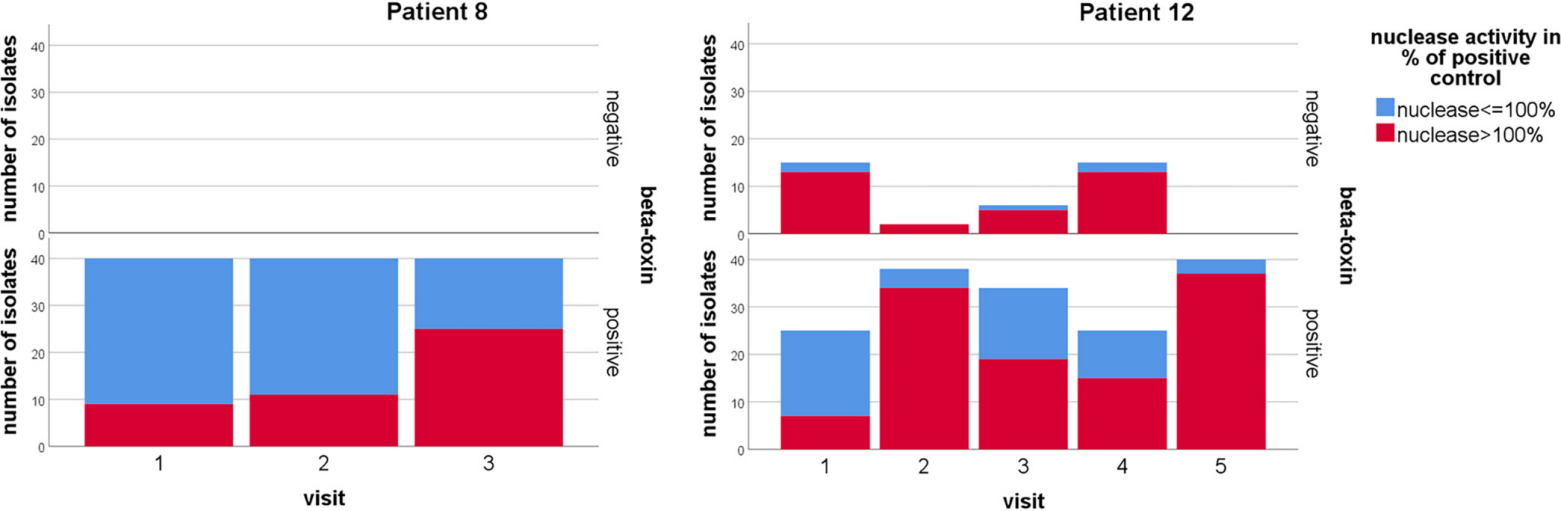
Increasing nuclease activity in β-toxin-positive S. aureus isolates. Nuclease activity was measured for all S. aureus isolates and correlated with β-toxin status of isolates as exemplified here for isolates of patients 8 and 12. The graph distinguishes β-toxin-negative and -positive isolates, showing an increase of nuclease activity in β-toxin-positive isolates over time (*P* < 0.001), as analyzed by GEE.

Our longitudinal study allowed the analysis of bacterial traits by estimation with the GEE model in a timely fashion showing that S. aureus isolates positive for β-toxin had higher nuclease activity at the end of the study period than at the beginning as exemplified for patients 8 and 12 in Fig. 8 (*P* < 0.001) and for all patients in Fig. S4. Also, if isolates were mucoid or nonhemolytic, the number of SCVs increased longitudinally (*P* = 0.006 or *P* = 0.012, respectively), revealing that there is an ongoing adaptation of mucoid and nonhemolytic S. aureus isolates toward SCVs.

10.1128/mSphere.00358-21.4 FIG S4 Nuclease activity in β-toxin-positive S. aureus isolates. The percentage of isolates with low/high nuclease activity at every visit is shown and separated by β-toxin-positive and -negative S. aureus isolates. Half of the patients (patients 1, 2, 5 to 7, 10, and 12) carried only β-toxin-negative isolates in their specimens, while in the other patients, β-toxin-positive isolates were identified at least in one visit. Download FIG S4, PDF file, 0.1 MB. Copyright © 2021 Wieneke et al. 2021 Wieneke et al. https://creativecommons.org/licenses/by/4.0/ This content is distributed under the terms of the Creative Commons Attribution 4.0 International license.

**FIG 9:**
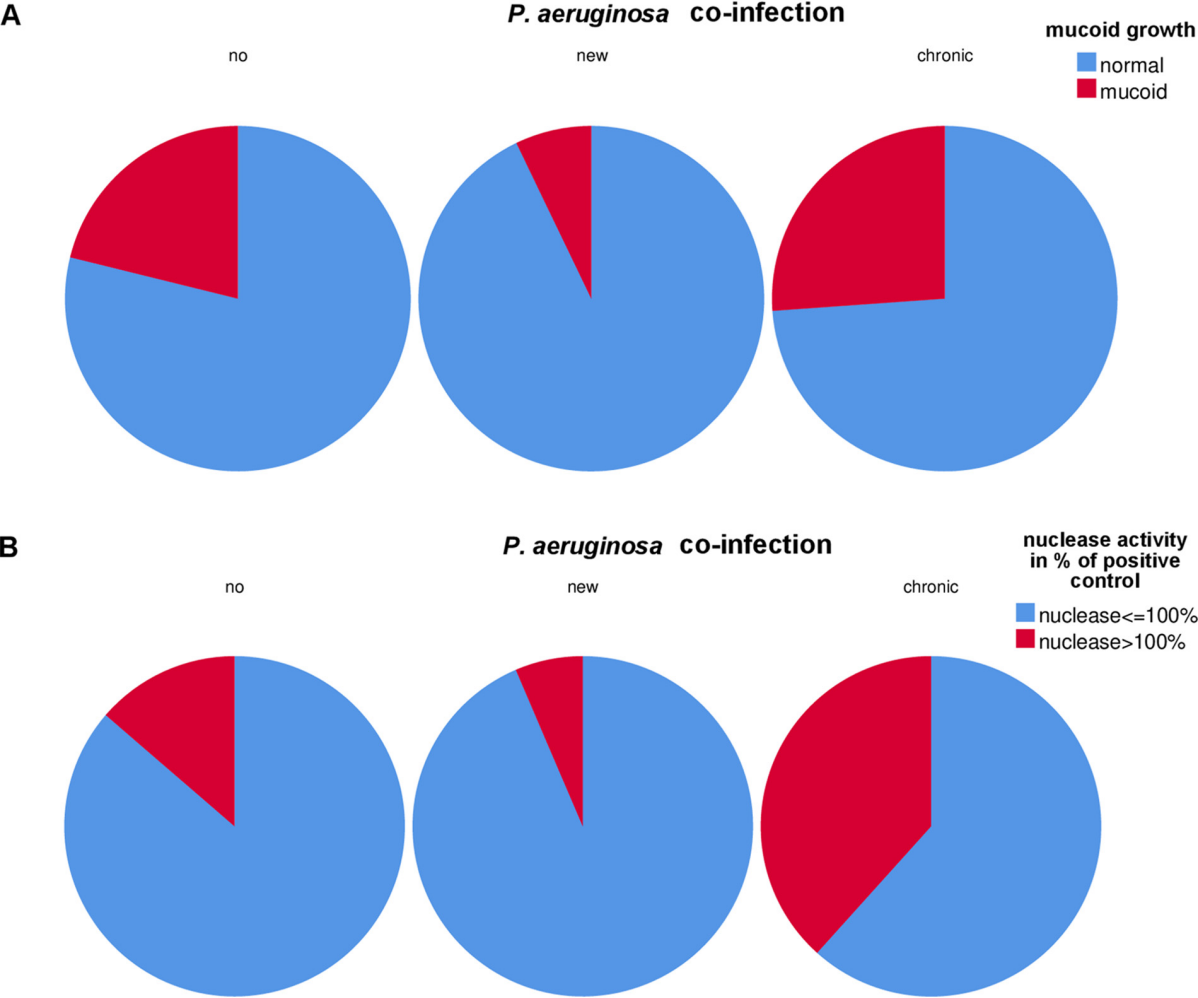
P. aeruginosa coinfection associated with mucoid growth and nuclease activity of S. aureus isolates. (A) The pie charts show that a new coinfection with P. aeruginosa was associated with a lower number of mucoid S. aureus isolates compared to patients with no coinfection, while in patients with chronic P. aeruginosa coinfection, the highest number of mucoid S. aureus isolates were recovered. (B) Also, the number of S. aureus isolates with high nuclease activity was highest in patients with chronic P. aeruginosa coinfection.

While there was a clear effect of P. aeruginosa on mucoidy of S. aureus (*P* = 0.024) (Fig. 9A), the global statistical test with patients grouped into three groups depending on the presence of P. aeruginosa (yes/new/no) showed only a trend for the association of isolates with higher nuclease activity (*P* = 0.07) (Fig. 9B). However, the comparison of the group of patients with chronic P. aeruginosa infection to no P. aeruginosa infection showed a significant association of isolates with higher nuclease activity (*P* = 0.04) (Fig. 9B) in the group of patients with chronic P. aeruginosa coinfection. In addition, P. aeruginosa turned out to affect the mean daily rate of adaptation (MDRA) of PTs (*P* = 0.018), which represents the mean daily rate of phenotypical changes of S. aureus (Table 2). Such a positive association of P. aeruginosa with S. aureus PTs suggests that there is continuous adaptation of S. aureus during competition with P. aeruginosa for optimized survival of S. aureus during coinfection.

## DISCUSSION

Airway infection with S. aureus in CF patients is increasing (2, 3), and in countries where CF patients are not given continuous antistaphylococcal treatment, the numbers of patients with persistent S. aureus infection are high (2, 3). In contrast to studies with P. aeruginosa (34, 35), which was the most isolated pathogen until recently, there are only a few studies investigating the diversity of S. aureus (24, 36) during persistent infection and its impact on CF lung disease.

By using deep cultural analysis, we revealed several important findings. First, 7 phenotypes (PTs) including normal and hemolytic isolates, SCVs as well as mucoid and β-toxin-positive isolates were most abundant within 32 identified PTs and represented more than 75% of isolates. Although almost all patients were infected by a different S. aureus clone as determined by *spa* typing, our data revealed that there were some typical S. aureus PTs present in the airways of chronically infected patients, which points to convergent evolution of S. aureus phenotypes, a phenomenon that has been described for P. aeruginosa but not for S. aureus in CF so far (37).

Our data showed that there was a high diversity of S. aureus PTs, which differed within and between patients, showing a dynamic turnover of PTs (Fig. 4 and 5 and Table 3; see also Fig. S3 and S4 in the supplemental material). Such diversity and dynamics have been shown in the study by Mowat et al. for P. aeruginosa (34). In this study, the authors also used deep phenotypical characterization (34), except that in their study, only patients who carried the same epidemic P. aeruginosa clone (LES) were included. However, our data of phenotypical dynamics in different S. aureus clones point to a common bacterial adaptational trait occurring in CF airways.

In our study, we determined a high prevalence of mucoid isolates in patients, which was higher than in our earlier studies (16, 17). This could be explained not only by the selection of patients for our study, who were adults and infected by S. aureus for more than 10 years, but also by our deep culturing conditions, which most likely allowed the identification of more mucoid isolates, most of them producing large amounts of biofilm consisting of the polysaccharide termed PIA (polysaccharide intercellular adhesin) (16, 17, 38, 39). Bernardy et al. recently reported that 69% of 64 S. aureus isolates from CF patients that they tested would produce polysaccharides, which is an extremely high number of isolates which are suggested to produce PIA (24). However, the authors tested the growth of their strains only on Congo red agar without performing biofilm assays. Also, the colony morphology as presented by the authors does not really reflect the phenotype of mucoid isolates with the 5-bp deletion in the intergenic region of the *ica* operon as described by Jefferson et al. (40) and us, as exemplified in Fig. 1.

While the Simpson diversity index (SDI) is often used in ecological or microbiome studies (41), we used this algorithm to calculate the pheno- and genotypic diversity of S. aureus, which revealed that SDIs of *spa* types were more homogeneous compared to SDIs of phenotypes. Changes in *spa* types were mostly due to mutations in the variable number of tandem repeat (VNTR) region and not due to new incoming clones (Tables 2 and 4). Also, the diversity of PTs of *spa* types seemed to be rather related to the individual patient as to the clonal background (Table 6). However, the number of investigated patients is too low to draw definite conclusions about such a relation.

Furthermore, the diversity of PTs as calculated by the SDI was positively associated with IL-6, which turned out to be the most sensitive inflammatory parameter of lung disease in our study, indicating that the presence of divergent S. aureus PTs has a more severe impact on the inflammatory status within airways compared to a homogeneous S. aureus population. On the other hand, the inflammatory status within CF airways could also drive S. aureus diversity, as IL-6 was also associated with SDIs.

In addition, the activity of nuclease was associated with IL-6 and CRP, suggesting that isolates with high nuclease activity were selected in inflamed airways most likely due to their ability to facilitate escape from NETs (18, 19), which are abundant in CF airways (20) and which are especially increased in CF due to delayed apoptosis of CF neutrophils (42). Whether S. aureus, which highly expresses nuclease, has any effect on the viscosity of airway secretions in CF, which are especially caused by extracellular DNA of neutrophils (43) remains to be established.

During the last years, a new focus of CF research, which investigates the pathogenesis of coinfection of important CF pathogens, has been established not only in clinical studies (11, 12, 44) but also in basic research (24, 45, 46) and summarized recently in the paper by Camus et al. (47). Our observational clinical study adds some novel information to this topic. In patients coinfected with P. aeruginosa, the number of mucoid S. aureus isolates increased. High biofilm formation most likely protects S. aureus against P. aeruginosa, which is known to express small molecules such as 4-hydroxy-2-heptylquinoline-*N*-oxide (HQNO) that harm S. aureus (48). Interestingly, Fugère et al., who studied coinfection with clinical P. aeruginosa and S. aureus isolates showed that the supernatants of some but not all P. aeruginosa isolates activated biofilm-stimulatory activities in a control S. aureus strain, but to a lower extent in clinical S. aureus isolates, which was positively correlated with the levels of quinolones (48). Thus, coinfection with P. aeruginosa does not only select for SCVs (49) but also for mucoid phenotypes as another survival mechanism for S. aureus. Also, P. aeruginosa coinfection increased the rate of phenotypically divergent isolates as assessed by the mean daily rate of adaptation (MDRA), which implies that in light of the insurance hypothesis (50), the survival of S. aureus is increased during coinfection if a higher number of diverse PTs is present.

Our findings revealed some new findings of the diversity of S. aureus during long-term chronic infection especially during coinfection with P. aeruginosa as well as for cultural diagnostics in the routine microbiology laboratory. Usually, in routine diagnostics, only one or two colonies of a species are used for species identification and susceptibility testing. Therefore, the extent of diversity of isolates in specimens is usually not reported and difficult-to-identify PTs such as SCVs or mucoid isolates are rarely identified. In this time of matrix-assisted laser desorption ionization−time of flight mass spectrometry (MALDI-TOF MS) analysis, costs for species identification are low, which should allow us to discriminate more phenotypically diverse colonies from primary specimens (51).

One limitation of our study is that we analyzed a quite small number of adult patients with long-term S. aureus positivity for more than 10 years from only two CF centers, which might not reflect the overall situation in other CF centers and patients with less chronic S. aureus infection, especially in younger patients. However, since patients with persistent S. aureus infection are also observed in many other centers in the world (2, 3) and the fact that most patients carried different S. aureus *spa-*CCs point out that our data might also apply to many other CF patients who are chronically infected by S. aureus. Another aspect that we did not address during our phenotypical characterization is the presence of hypermutable strains (52), which were first described for P. aeruginosa and which could influence the diversity of PTs and *spa* types. The presence of mutator strains in CF has been shown to occur also in S. aureus isolates and here especially in thymidine-dependent SCVs (53).

In conclusion, our deep cultural and genotypic analyses of S. aureus diversity revealed that in CF patients with long-term airway infection, a high diversity of S. aureus phenotypes is present, which is highly dynamic and associated with inflammation of airways as assessed by inflammatory parameters such as IL-6, S100A8/9, and CRP in sera and P. aeruginosa coinfection.

## MATERIALS AND METHODS

### Study design.

Sputa of 14 patients who attended the CF outpatient clinics of the University Hospital Münster or the Clemenshospital Münster, Germany, were analyzed during a 1-year period. These patients were selected, because they were persistently positive for S. aureus for more than 10 years and regularly provided sputum within the last years. Permission was granted from the Ethical Committee of the Medical Association of Westfalia and the Westfalian-Wilhelms University Münster (2010-155-f-S). Written informed consent was obtained from all patients.

Sputa were directly sent to the laboratory. The further workup of sputa followed standard procedures for the culture of CF specimens (54, 55) except that at first sputa were homogenized using a Retsch mill (Retsch GmbH, Haan, Germany) and frozen in aliquots at −80°C except for the portion that was used for this study and the routine microbiological diagnostic. The sputum was diluted and used to inoculate Columbia blood agar (Columbia blood agar base [catalog no. CM0331; Oxoid]) and CAP agar (Oxoid GmbH, Wesel, Germany) to suppress Gram-negative rods and facilitate growth of Gram-positive cocci and MacConkey agar (Oxoid GmbH, Wesel, Germany) to enumerate bacteria. Sputum was streaked on additional agar plates without dilution: chocolate agar (Becton Dickinson GmbH, Heidelberg, Germany), CAP agar, Columbia blood agar, MacConkey agar, brain heart infusion (BHI) (Merck, Darmstadt, Germany), Burkholderia agar (BCA) (Mast Group Ltd., Bootle, UK), and Sabouraud agar (Thermo Fisher Scientific GmbH, Dreieich, Germany) according to standard procedures for CF specimens (55).

Forty S. aureus isolates from the diluted sputum cultures grown on Columbia blood and CAP agar were chosen to ensure that all different phenotypes were represented as suggested for P. aeruginosa by Mowat et al. (34). Isolates were confirmed by MALDI-TOF analysis (MALDI-TOF MS, Bruker, Billerica, MA, US) and characterized for size (normal/SCV) and mucoidy (nonmucoid/mucoid) (16), β-toxin (positive/negative), hemolysis (positive/negative), and pigmentation on blood (gray, yellow, and white), Schaedler and Congo red agar (CRA) plates to confirm the mucoid phenotype of S. aureus isolates (16). The phenotypes of the isolates were characterized according to size, mucoidy, hemolysis, β-toxin, which is visible as a double hemolytic zone around the colonies, and pigmentation by two technicians as follows: for size, 1 for normal size and 2 for SCV, mixture or SCV and normal or fried egg growth (56); for mucoid growth, 1 for nonmucoid and 2 for mucoid (16); for hemolysis, 1 for nonhemolytic and 2 for hemolytic (57, 58); for β-toxin, 1 for negative and 2 for positive (57, 58); and for pigmentation on Columbia blood agar, 1 for gray, 2 for white, and 3 for yellow.

### *spa* sequence typing.

*spa* typing was performed by amplification of the variable region of protein A by PCR with ensuing sequencing according to Harmsen et al. (26).

### Patient’s clinical data.

At each visit, the patients’ clinical condition as well as information about recent antibiotic therapy were documented in case report forms. Treatment with the following antibiotics were suggested to have an effect on S. aureus: β-lactam antibiotics except aztreonam, macrolides, clindamycin, fluorquinolones, trimethoprim-sulfamethoxazole, fosfomycin, and tobramycin inhalation.

Inflammatory parameters S100A8/9 (59), CRP (32), and interleukin 6 (IL-6) (33) were analyzed in sera. Lung function was calculated as FEV_1_ % predicted as described by Quanjer et al. (60). Exacerbation was defined by the criteria established by Fuchs et al. (61): at least 4 out of 12 evaluated clinical symptoms: change in sputum and sinus discharge, new or increased dyspnea, cough, hemoptysis, malaise, weight loss, sinus tenderness, temperature over 38°C, radiographic changes indicative of lung infection, and decrease in pulmonary function by 10%.

### S100A8/9, IL-6, and CRP.

The levels of S100A8/A9 were determined by a sandwich enzyme-linked immunosorbent assay (ELISA) as described by Frosch et al. (59). IL-6 levels in sera were determined by ELISA (R&D, Wiesbaden, Germany) according to the manufacturer’s instructions. The assay was characterized by intra-assay precisions of 4.2%, 1.6%, and 2.0% at low, medium, and high analyte levels, respectively. The minimum detectable dose of IL-6 was less than 0.7 pg/ml. The cutoff value for IL-6 (12 pg/ml) corresponds to the highest IL-6 level observed in a control population of 40 apparently healthy individuals and is very close to the cutoff value of 15 pg/ml, which has been suggested in previously published studies (62, 63). CRP concentrations were determined in the hospital laboratory by turbidimetry (Roche, Mannheim, Germany) on a Cobas c502 automated analyzer. The intra-assay and interassay variabilities were <2.8 % and <6.1%, respectively.

### Biofilm assay.

A static 96-well polystyrene biofilm microtiter plate assay was performed to evaluate biofilm formation (16). For thymidine-dependent SCVs (TD-SCVs), thymidine (100 μg/ml; Fluka Chemie, Buchs, Switzerland) was added to the medium (13, 64). Staphylococcus carnosus TM300 was used as a negative control (65), and Staphylococcus epidermidis RP62A was used as a positive control (66). At 655 nm, the absorbance of the crystal violet staining was measured photometrically (BioTek Synergy HTX Gen5 microplate reader and Imager software). The final value of biofilm formation was given as a percentage of the value of the positive control. As S. aureus usually does not form biofilm in high degrees, we arbitrarily defined the levels of low and high biofilm formation as follows: isolates with low biofilm formation had less than 10% biofilm mass of the positive control, and isolates with high biofilm formation had more than 10% biofilm mass compared to the positive control.

### Nuclease activity assay.

Nuclease activity analysis of S. aureus isolates was accomplished by a FRET assay (18). Briefly, bacteria were grown in 200 μl BHI in a 96-well plate, and the optical density at 578 nm (OD_578_) for 16 to 18 h was measured. Twenty microliters of this overnight culture was incubated in a 1:10 dilution for 4 h in a 96-well plate. Twenty microliters from this culture was diluted 1:200 in reaction buffer (50 mM Tris-HCl, 5 mM CaCl, 100 μg/ml bovine serum albumin [BSA] [pH 7.9] [30% NaOH]). Twenty microliters was added to a 180-μl solution of the molecular beacon (Eurofins Genomics; 5′→3′; 6-carboxyfluorescein [FAM]-CGAATTCC-TTTTT-GGAATTCG-black hole quencher 1 [BHQ1]; 10 μM, diluted 1:100 in reaction buffer, final concentration, 0.1 μM) in a black 96-well plate (Corning Incorporated; 96-well black flat-bottom polystyrene plate). The increase of fluorescence (*V*_max_) was measured (BioTek Synergy HTX Gen5 microplate reader and Imager software). S. aureus strain AH1263 was used as a positive control, and the Δ*nuc* mutant AH1680 was used as a negative control (67). Growth-fitted nuclease activity was achieved by relative nuclease activity multiplied against a factor resulting from OD_578_ values of the analyzed CF isolate, obtained after 4 h of incubation in fresh BHI against the OD_578_ value of AH1263 (100% control). For S. aureus isolates, we defined low and high nuclease activity depending on the comparison to the positive control with less than 100% being low nuclease activity and higher than 100% being high nuclease activity. If isolates were mucoid, they were grown in BHI with dispersin B (5 μg/ml; Kane Biotech Inc., MB, Canada) to allow steady growth by avoiding clustering because of increased biofilm production (16). For TD-SCVs, thymidine was added to the medium (100 μg/ml).

### Simpson’s diversity index.

The Simpson’s diversity index (SDI) was calculated to compare the phenotypic diversity of isolates within single sputum specimens and between sputa within and between individuals during our study according to *spa* types and phenotypes. The SDI was calculated using the following formula: $D=1-\sum \left(\frac{n(n-1)}{N(N-1)}\right)$ with *n* being the number of isolates with a specific characteristic and *N* being the number of all isolates from one sputum specimen (*n* = 40). Therefore, if *D* = 1, there is a high diversity of isolates, and if *D* = 0, there is no diversity of isolates with all isolates exhibiting the same characteristic (homogeneous population).

### Mean daily rate of alteration.

For each patient, the mean daily rate of alteration (MDRA) for PTs and *spa* types was calculated as follows as described by Roden et al. (68):

$$\text{MDRA}(i)=\frac{1}{n_{i}}\sum_{\kappa \varepsilon \{1,2\ldots n_{i}\}}\frac{\Delta {\text{phenotype}}_{\kappa }^{i}}{\Delta t_{\kappa }^{i}}$$

with *n_i_* as the number of visits of patient *i*, , and $\Delta {\text{phenotype}}_{\kappa }^{i}$ as the number of changed species (loss or gain) at visit κ for patient *i* compared to the previous visit of patient *i*, and $\Delta t_{\kappa }^{i}$ as time in days between visits κ and previous visit.

### Statistical analysis.

Statistical analysis was performed using IBM SPSS Statistics v.26 software. To evaluate the statistical significance, we used a general estimating equations (GEE) model and set the significance level at α < 0.05. The following parameters were analyzed as binary categories: mucoid/nonmucoid, hemolytic/nonhemolytic, β-toxin positive/negative, SCV/normal size, biofilm production up to 10% and higher than 10% compared to the positive control, nuclease activity up to 100% and higher than 100% compared to the positive control, and exacerbation yes/no. Lung function (FEV_1_% predicted), inflammatory parameters (S100A8/9, CRP, and IL-6), and the Simpson’s diversity indices of phenotypes, biofilm, and nuclease were analyzed as scaled parameters. To investigate changes in time, “months after inclusion to the study” was used as an ordinal variable.

We applied generalized estimation equations to model the inherent correlation structure of the study data. In particular, within our study, clustering occurs on patient level due to repeated measurements over time (visits) and the assessment of up to distinguishable 40 S. aureus isolates per visit. To model correlation within the data structure, a working correlation structure needs to be assumed. We chose a first-order autoregressive (AR(1)) structure. The AR(1) structure implies that correlation between measurements decreases with the mutual distance between two assessment time points. We considered this a reasonable scenario for our repeated measured data. In general, GEEs allow a consistent estimation of model parameters, even under slight misspecification of the structure of the working correlation matrix. GEEs directly estimate marginal models compared to generalized linear mixed model, where the marginal estimation of a random effect model needs to be made in a second step. Marginal modeling, especially, should be applied when inference over population-averaged parameters are the major analysis aim.

For the GEE model’s working correlation matrix, we specified a first-order autoregressive relationship (AR(1)) structure. We defined the patients as the subject variable, while their visits and the 40 S. aureus isolates per visit were defined as within-subject variables. We used a linear type of model for scaled response variables, while we used a binary logistic type of model for the binary response variables. To specify the model effects, all predictors (factors and covariates) were used as main effects unless for developments in time, model effects were defined as interaction of predictor and “months after inclusion to the study.” Type III tests were used to assess model effects for significance.

10.1128/mSphere.00358-21.6 TABLE S2 S. aureus *spa* types during the prospective study. Download Table S2, DOCX file, 0.04 MB. Copyright © 2021 Wieneke et al. 2021 Wieneke et al. https://creativecommons.org/licenses/by/4.0/ This content is distributed under the terms of the Creative Commons Attribution 4.0 International license.
