# Supplementary material for: Association of Diverse Staphylococcus aureus Populations with Pseudomonas aeruginosa Coinfection and Inflammation in Cystic Fibrosis Airway Infection
Source: mSphere. 2021 Jun 23;6(3):e00358-21. doi: 10.1128/mSphere.00358-21 (PMC8265651; doi:10.1128/mSphere.00358-21)
Supplement: TABLE S1 [file msphere.00358-21-st001.docx]

**Table S1.** ***S. aureus* phenotypes during the prospective study**

| # | **phenotype** | **size^*^** | **mucoid**^†^ | **hemolysis**^‡^ | **ß-toxin**^§^ | **pigment** ^ll^ | **# of**  **isolates** | **percent** | **# of**  **patients** | **# of *spa* types** |
| --- | --- | --- | --- | --- | --- | --- | --- | --- | --- | --- |
| 1 | 4 | 1 | 1 | 2 | 1 | 1 | 868 | 37 | 12 | 27 |
| 2 | 7 | 1 | 1 | 2 | 2 | 1 | 231 | 10 | 5 | 7 |
| 3 | 1 | 1 | 1 | 1 | 1 | 1 | 227 | 10 | 11 | 14 |
| 4 | 6 | 1 | 1 | 2 | 1 | 3 | 136 | 6 | 8 | 11 |
| 5 | 30 | 2 | 2 | 2 | 1 | 1 | 116 | 5 | 7 | 11 |
| 6 | 22 | 2 | 1 | 2 | 1 | 1 | 114 | 5 | 9 | 15 |
| 7 | 15 | 1 | 2 | 2 | 1 | 3 | 105 | 5 | 4 | 6 |
| 8 | 13 | 1 | 2 | 2 | 1 | 1 | 93 | 4 | 9 | 10 |
| 9 | 17 | 2 | 1 | 1 | 1 | 1 | 73 | 3 | 6 | 17 |
| 10 | 24 | 2 | 1 | 2 | 1 | 3 | 73 | 3 | 3 | 4 |
| 11 | 27 | 2 | 2 | 1 | 1 | 1 | 62 | 3 | 5 | 7 |
| 12 | 10 | 1 | 2 | 1 | 1 | 1 | 38 | 2 | 4 | 4 |
| 13 | 29 | 2 | 2 | 1 | 2 | 1 | 35 | 2 | 2 | 2 |
| 14 | 25 | 2 | 1 | 2 | 2 | 1 | 31 | 1 | 4 | 5 |
| 15 | 5 | 1 | 1 | 2 | 1 | 2 | 17 | 1 | 2 | 2 |
| 16 | 8 | 1 | 1 | 2 | 2 | 2 | 15 | 1 | 2 | 3 |
| 17 | 18 | 2 | 1 | 1 | 1 | 2 | 14 | 1 | 2 | 6 |
| 18 | 28 | 2 | 2 | 1 | 1 | 2 | 13 | 1 | 3 | 4 |
| 19 | 14 | 1 | 2 | 2 | 1 | 2 | 11 | 0 | 2 | 3 |
| 20 | 16 | 1 | 2 | 2 | 2 | 1 | 10 | 0 | 2 | 2 |
| 21 | 23 | 2 | 1 | 2 | 1 | 2 | 8 | 0 | 3 | 4 |
| 22 | 31 | 2 | 2 | 2 | 1 | 3 | 8 | 0 | 1 | 1 |
| 23 | 11 | 1 | 2 | 1 | 1 | 2 | 7 | 0 | 2 | 2 |
| 24 | 21 | 2 | 1 | 1 | 2 | 2 | 3 | 0 | 1 | 3 |
| 25 | 12 | 1 | 2 | 1 | 1 | 3 | 2 | 0 | 1 | 1 |
| 26 | 19 | 2 | 1 | 1 | 1 | 3 | 2 | 0 | 1 | 1 |
| 27 | 26 | 2 | 1 | 2 | 2 | 2 | 2 | 0 | 1 | 1 |
| 28 | 2 | 1 | 1 | 1 | 1 | 3 | 1 | 0 | 1 | 1 |
| 29 | 3 | 1 | 1 | 1 | 2 | 1 | 1 | 0 | 1 | 1 |
| 30 | 9 | 1 | 1 | 2 | 2 | 3 | 1 | 0 | 1 | 1 |
| 31 | 20 | 2 | 1 | 1 | 2 | 1 | 1 | 0 | 1 | 1 |
| 32 | 32 | 2 | 2 | 2 | 2 | 1 | 1 | 0 | 1 | 1 |

^*^ size: according to the size of the colonies on Columbia blood agar: 1 = normal; 2 = SCVs

^†^ mucoid: mucoid growth at least on one of the different agars (Columbia blood agar, Schaedler agar or Kongored agar) – 1 = non-mucoid; 2 = mucoid

^‡^ hemolysis: 1 = no hemolysis; = hemolysis (on Columbia blood agar)

^§^ ß-toxin: 1 = no ß-toxin; 2 = ß-toxin

^ll^ pigment: 1 = grey, 2 = white, 3 = yellow
