# Supplementary material for: Association of Diverse Staphylococcus aureus Populations with Pseudomonas aeruginosa Coinfection and Inflammation in Cystic Fibrosis Airway Infection
Source: mSphere. 2021 Jun 23;6(3):e00358-21. doi: 10.1128/mSphere.00358-21 (PMC8265651; doi:10.1128/mSphere.00358-21)
Supplement: FIG S1 [file msphere.00358-21-sf001.pdf]

Patient 1

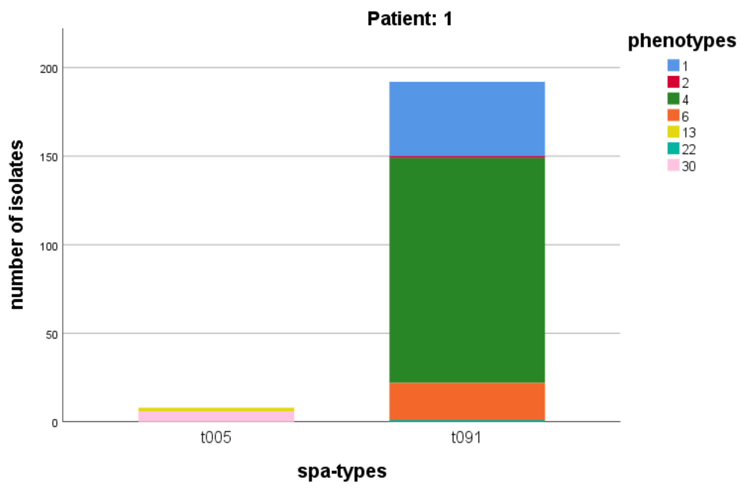

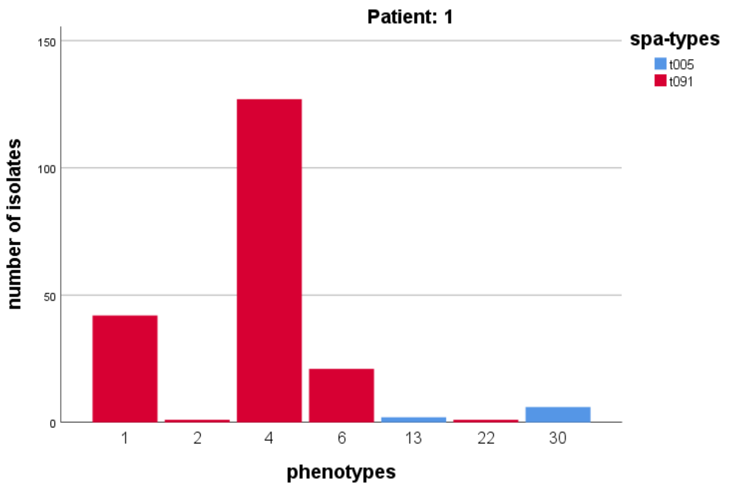

number of isolates		phenotypes 1	2	4	6	13	22	30	total
spa-types	t005	0	0	0	0	2	0	6	8
	t091	42	1	127	21	0	1	0	192
total		42	1	127	21	2	1	6	200

Patient 2

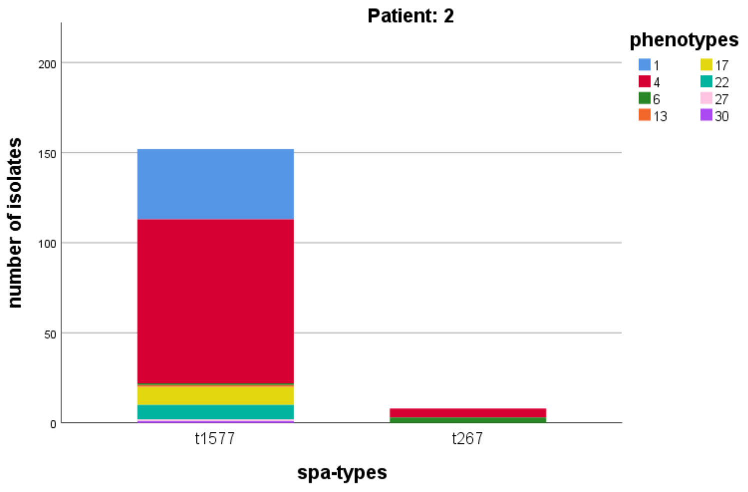

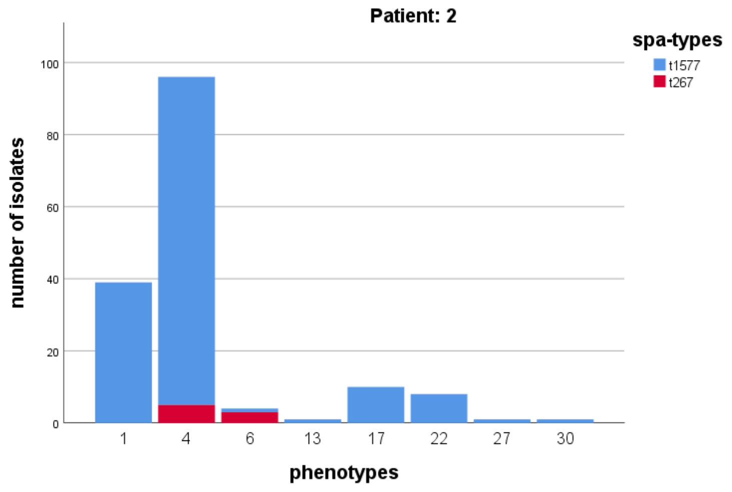

number of isolates		phenotypes 1	4	6	13	17	22	27	30	total
spa-types	t1577	39	91	1	1	10	8	1	1	152
	t267	0	5	3	0	0	0	0	0	8
total		39	96	4	1	10	8	1	1	160

Patient 3

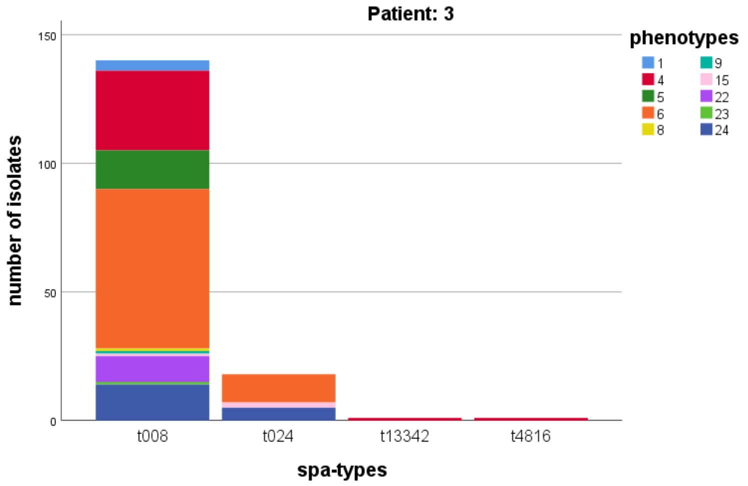

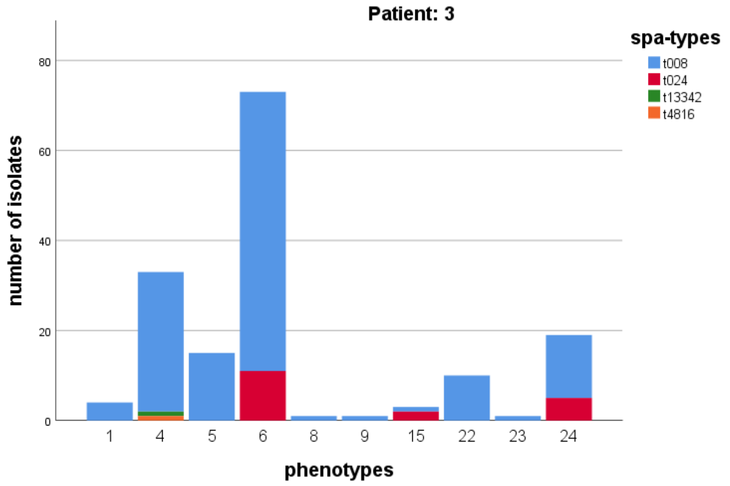

number of isolates		phenotypes 1	4	5	6	8	9	15	22	23	24	total
spa-types	t008	4	31	15	62	1	1	1	10	1	14	140
	t024	0	0	0	11	0	0	2	0	0	5	18
	t13342	0	1	0	0	0	0	0	0	0	0	1
	t4816	0	1	0	0	0	0	0	0	0	0	1
total		4	33	15	73	1	1	3	10	1	19	160

Patient 4

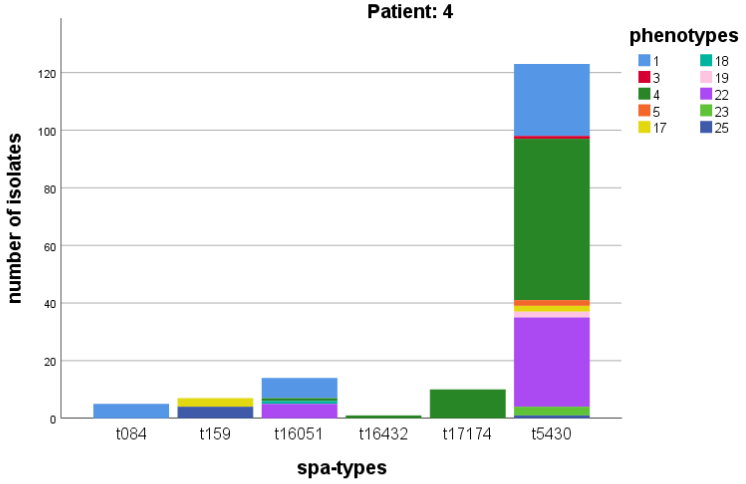

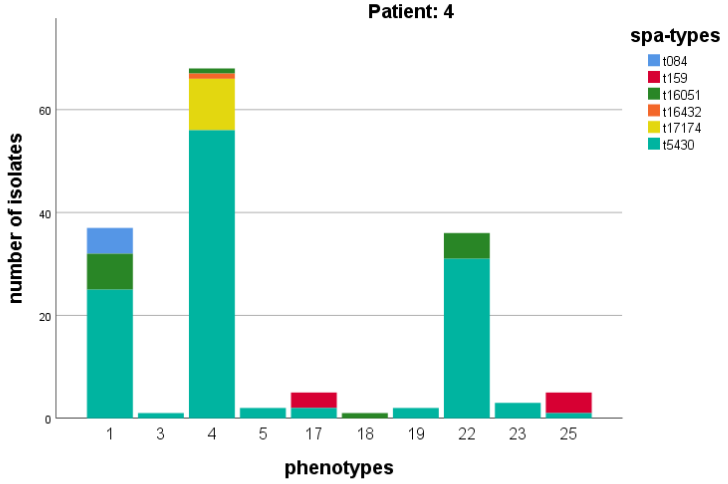

number of isolates		phenotypes										total
		1	3	4	5	17	18	19	22	23	25	
spa-types	t084	5	0	0	0	0	0	0	0	0	0	5
	t159	0	0	0	0	3	0	0	0	0	4	7
	t16051	7	0	1	0	0	1	0	5	0	0	14
	t16432	0	0	1	0	0	0	0	0	0	0	1
	t17174	0	0	10	0	0	0	0	0	0	0	10
	t5430	25	1	56	2	2	0	2	31	3	1	123
total		37	1	68	2	5	1	2	36	3	5	160

Patient 5

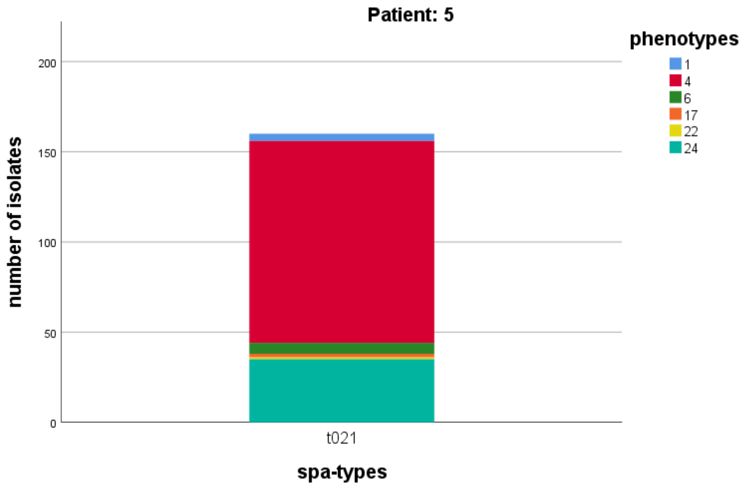

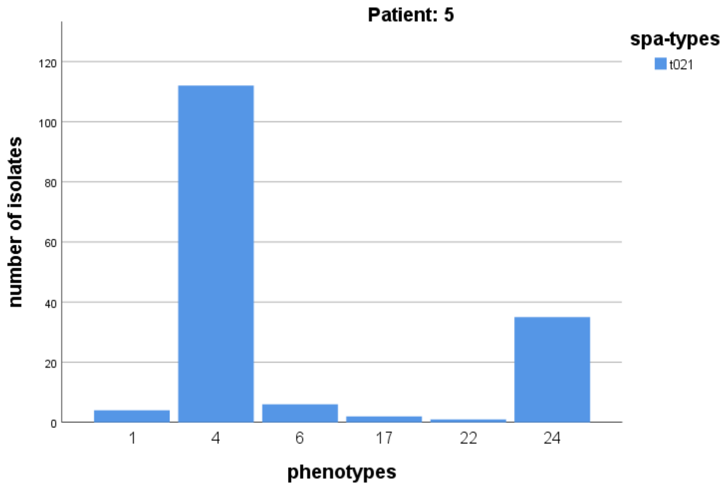

number of isolates		phenotypes 1	4	6	17	22	24	total
spa-types	t021	4	112	6	2	1	35	160
total		4	112	6	2	1	35	160

Patient 6

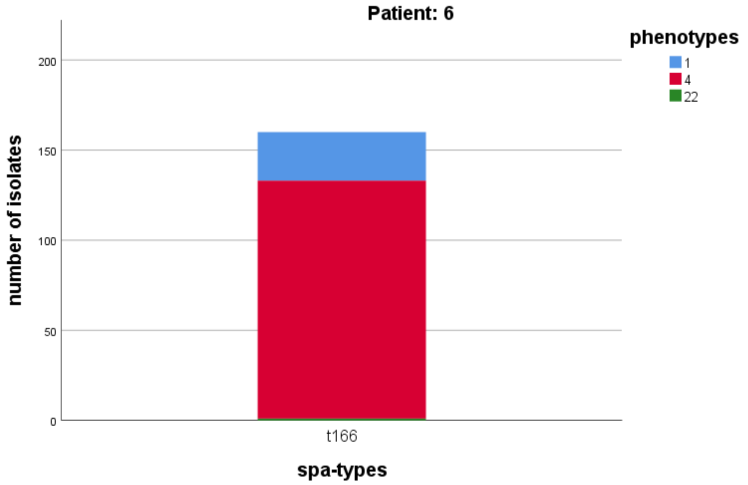

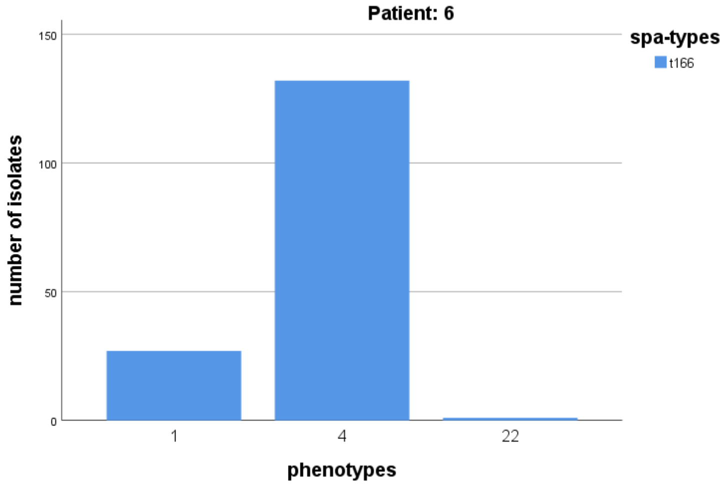

number of isolates		phenotypes 1	4	22	total
spa-types	t166	27	132	1	160
total		27	132	1	160

Patient 7

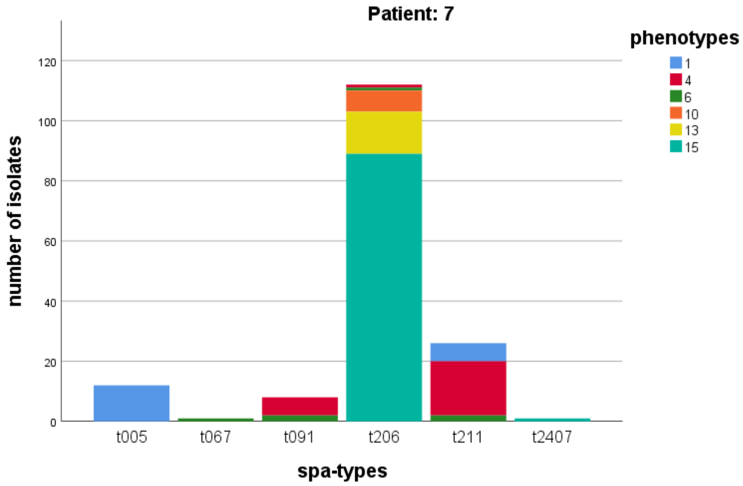

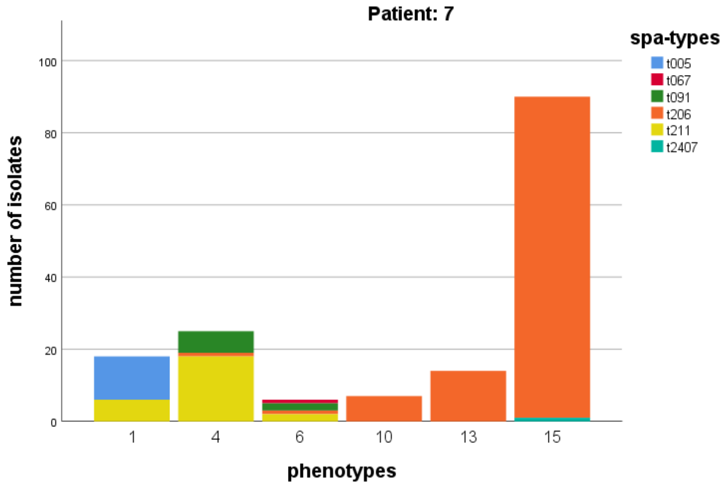

number of isolates		phenotypes						
		1	4	6	10	13	15	total
spa-types	t005	12	0	0	0	0	0	12
	t067	0	0	1	0	0	0	1
	t091	0	6	2	0	0	0	8
	t206	0	1	1	7	14	89	112
	t211	6	18	2	0	0	0	26
	t2407	0	0	0	0	0	1	1
total		18	25	6	7	14	90	160

Patient 8

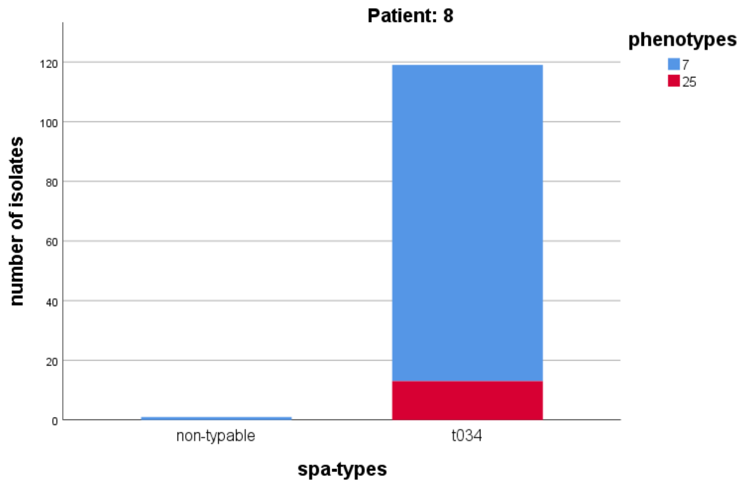

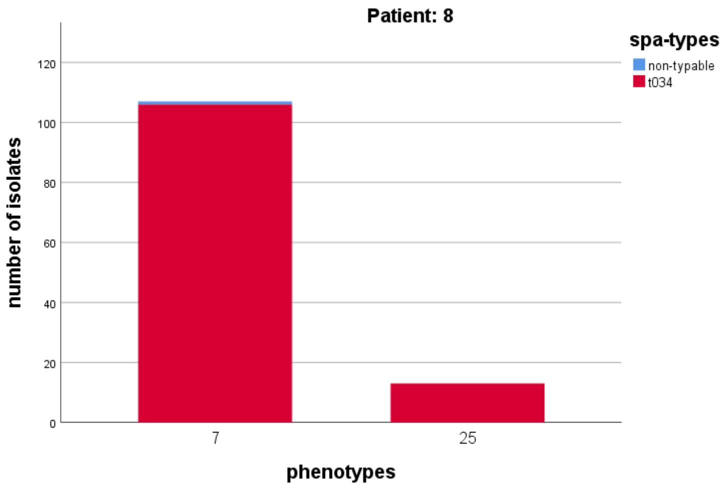

		phenotypes		
		7	25	total
spa-types	non-typable	1	0	1
	t034	106	13	119
total		107	13	120

Patient 9

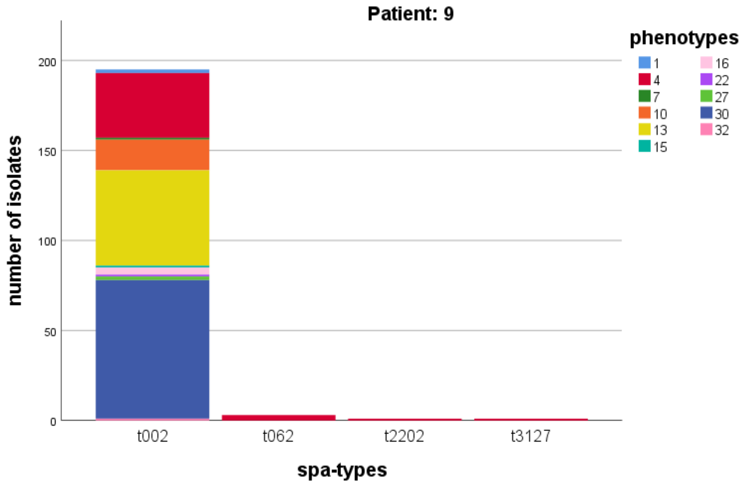

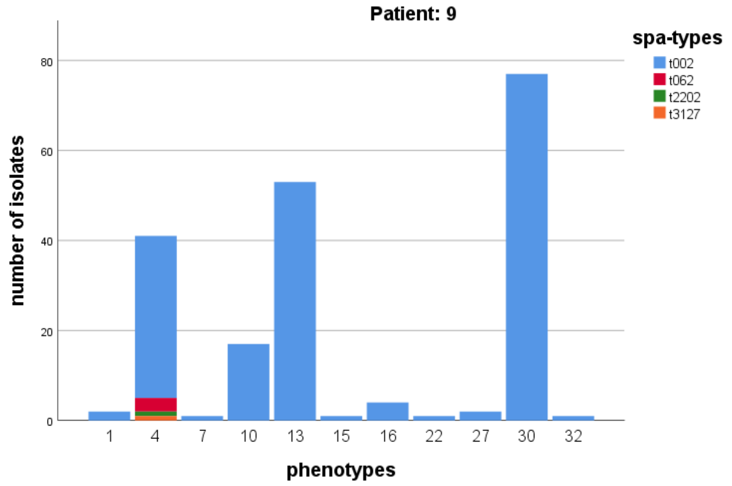

number of isolates		phenotypes											
		1	4	7	10	13	15	16	22	27	30	32	total
spa-types	t002	2	36	1	17	53	1	4	1	2	77	1	195
	t062	0	3	0	0	0	0	0	0	0	0	0	3
	t2202	0	1	0	0	0	0	0	0	0	0	0	1
	t3127	0	1	0	0	0	0	0	0	0	0	0	1
total		2	41	1	17	53	1	4	1	2	77	1	200

Patient 10

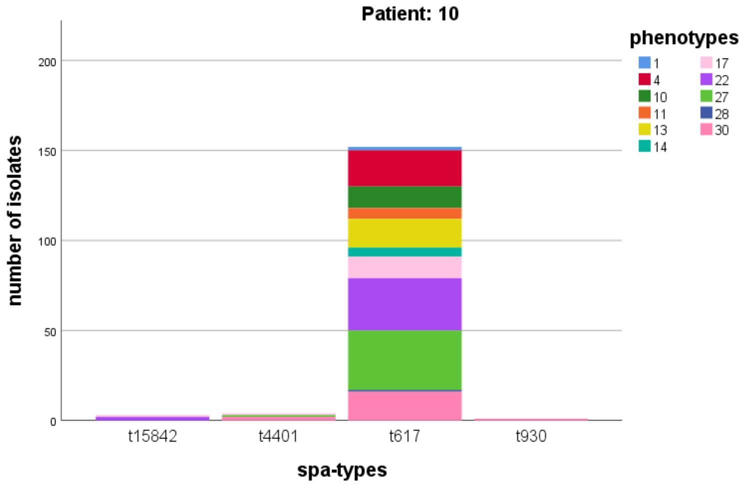

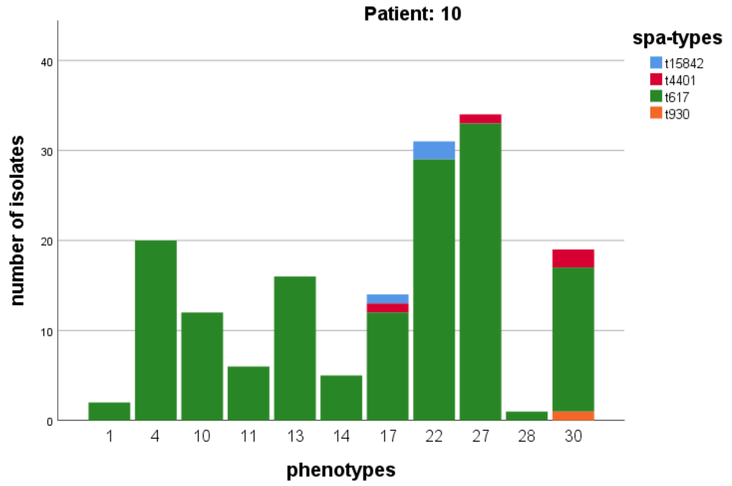

number of isolates		phenotypes											
		1	4	10	11	13	14	17	22	27	28	30	total
spa-types	t15842	0	0	0	0	0	0	1	2	0	0	0	3
	t4401	0	0	0	0	0	0	1	0	1	0	2	4
	t617	2	20	12	6	16	5	12	29	33	1	16	152
	t930	0	0	0	0	0	0	0	0	0	0	1	1
total		2	20	12	6	16	5	14	31	34	1	19	160

Patient 11

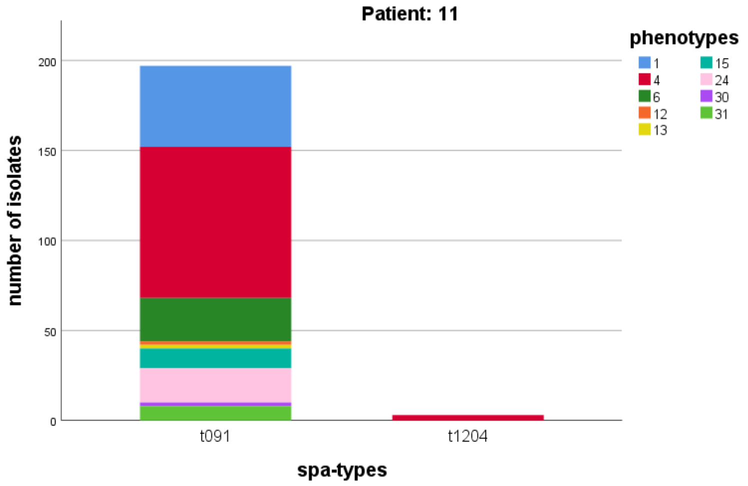

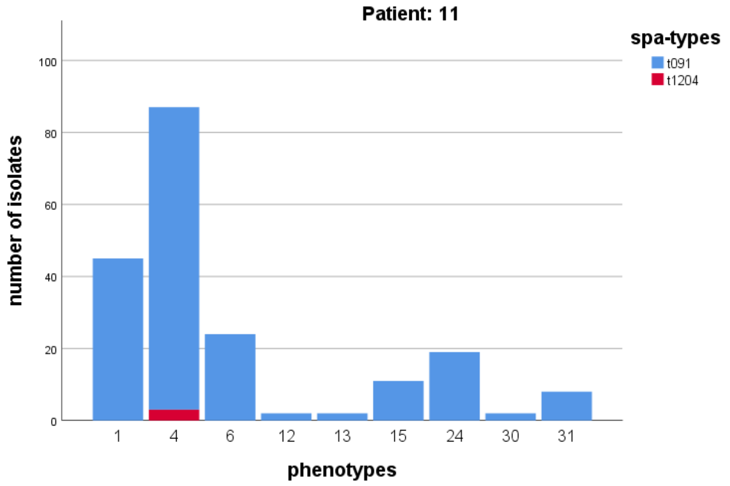

number of isolates		phenotypes 1	4	6	12	13	15	24	30	31	total
spa-types	t091	45	84	24	2	2	11	19	2	8	197
	t1204	0	3	0	0	0	0	0	0	0	3
total		45	87	24	2	2	11	19	2	8	200

Patient 12

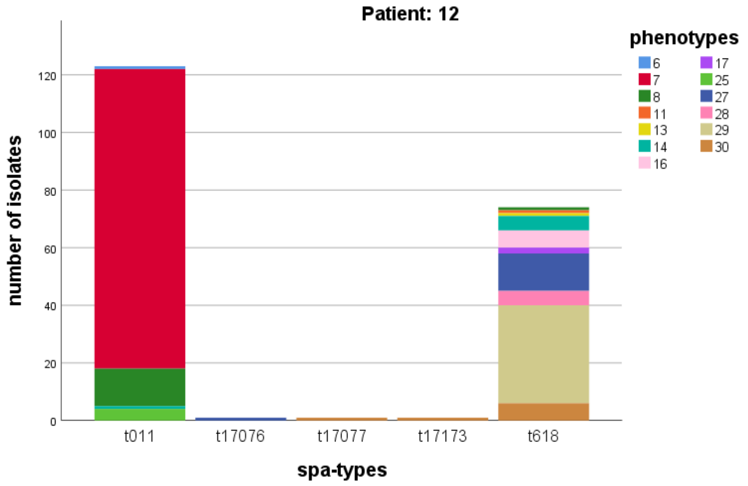

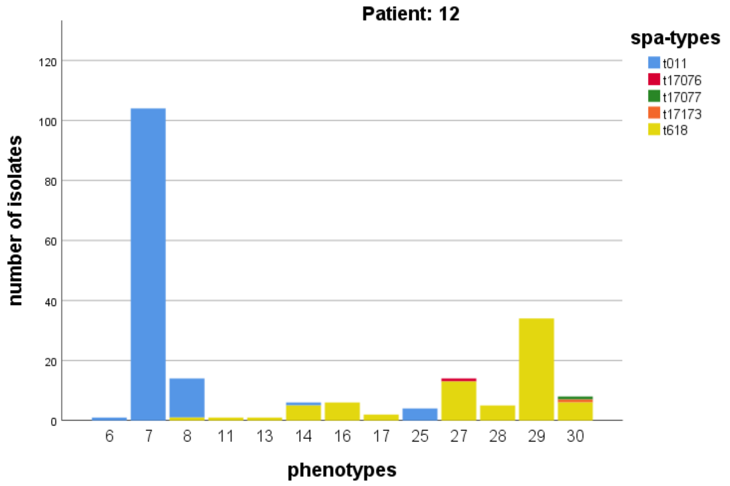

number of isolates		phenotypes													
		6	7	8	11	13	14	16	17	25	27	28	29	30	total
spa-types	t011	1	104	13	0	0	1	0	0	4	0	0	0	0	123
	t17076	0	0	0	0	0	0	0	0	0	1	0	0	0	1
	t17077	0	0	0	0	0	0	0	0	0	0	0	0	1	1
	t17173	0	0	0	0	0	0	0	0	0	0	0	0	1	1
	t618	0	0	1	1	1	5	6	2	0	13	5	34	6	74
total		1	104	14	1	1	6	6	2	4	14	5	34	8	200

Patient 13

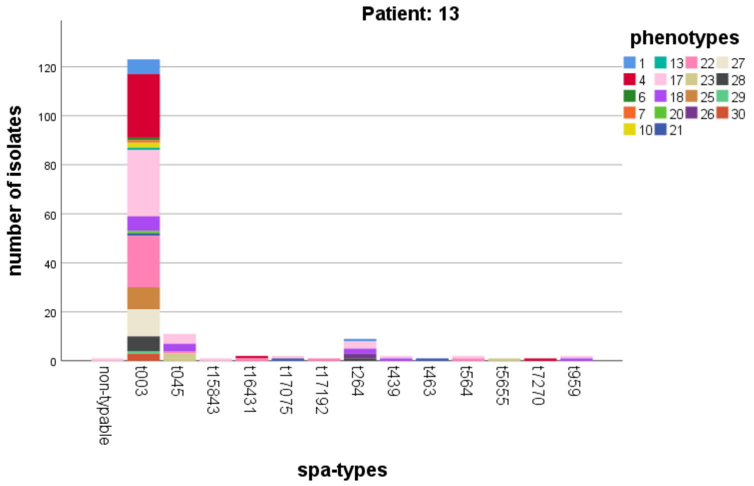

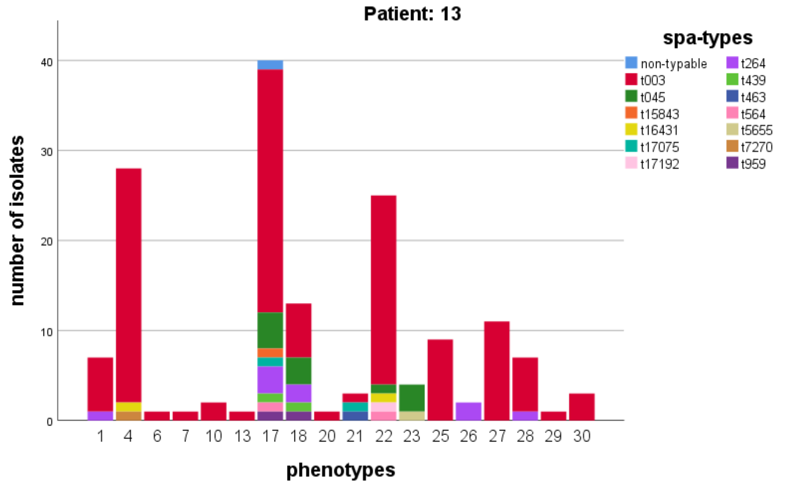

number of isolates		phenotypes																	
		1	4	6	7	10	13	17	18	20	21	22	23	25	26	27	28	29	30	Gesamt
spa-types	non-typable	0	0	0	0	0	0	1	0	0	0	0	0	0	0	0	0	0	0	1
	t003	6	26	1	1	2	1	27	6	1	1	21	0	9	0	11	6	1	3	123
	t045	0	0	0	0	0	0	4	3	0	0	1	3	0	0	0	0	0	0	11
	t15843	0	0	0	0	0	0	1	0	0	0	0	0	0	0	0	0	0	0	1
	t16431	0	1	0	0	0	0	0	0	0	0	1	0	0	0	0	0	0	0	2
	t17075	0	0	0	0	0	0	1	0	0	1	0	0	0	0	0	0	0	0	2
	t17192	0	0	0	0	0	0	0	0	0	0	1	0	0	0	0	0	0	0	1
	t264	1	0	0	0	0	0	3	2	0	0	0	0	0	2	0	1	0	0	9
	t439	0	0	0	0	0	0	1	1	0	0	0	0	0	0	0	0	0	0	2
	t463	0	0	0	0	0	0	0	0	0	1	0	0	0	0	0	0	0	0	1
	t564	0	0	0	0	0	0	1	0	0	0	1	0	0	0	0	0	0	0	2
	t5655	0	0	0	0	0	0	0	0	0	0	0	1	0	0	0	0	0	0	1
	t7270	0	1	0	0	0	0	0	0	0	0	0	0	0	0	0	0	0	0	1
	t959	0	0	0	0	0	0	1	1	0	0	0	0	0	0	0	0	0	0	2
total		7	28	1	1	2	1	40	13	1	3	25	4	9	2	11	7	1	3	159

Patient 14

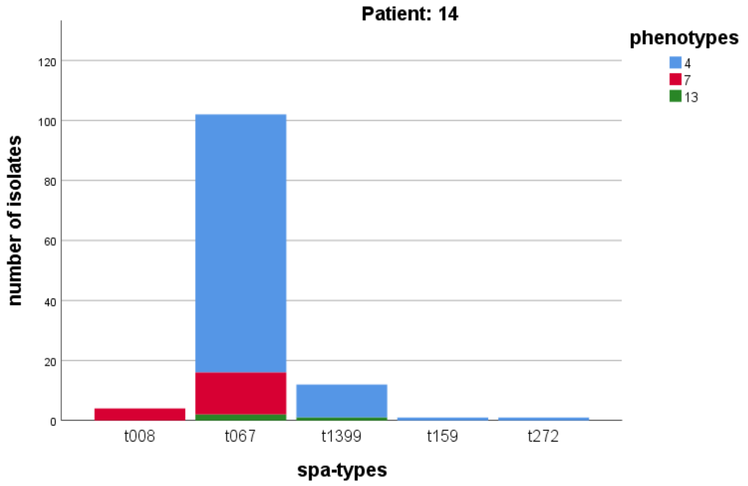

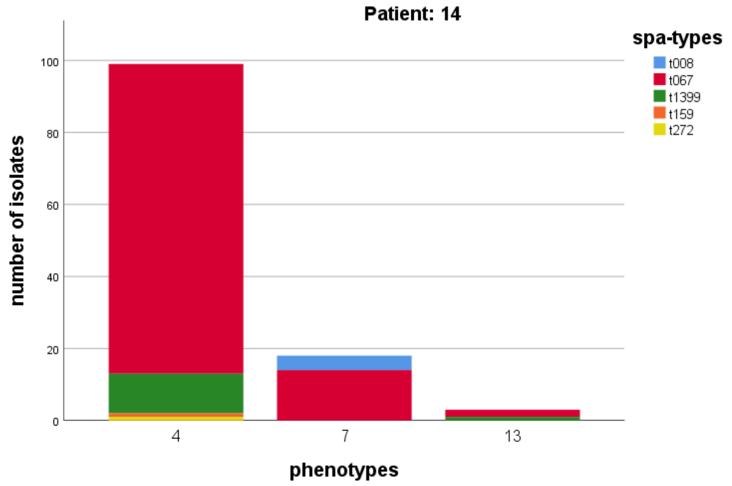

number of isolates		phenotypes 4	7	13	total
spa-types	t008	0	4	0	4
	t067	86	14	2	102
	t1399	11	0	1	12
	t159	1	0	0	1
	t272	1	0	0	1
total		99	18	3	120
