# Supplementary material for: Association of Diverse Staphylococcus aureus Populations with Pseudomonas aeruginosa Coinfection and Inflammation in Cystic Fibrosis Airway Infection
Source: mSphere. 2021 Jun 23;6(3):e00358-21. doi: 10.1128/mSphere.00358-21 (PMC8265651; doi:10.1128/mSphere.00358-21)
Supplement: FIG S2 [file msphere.00358-21-sf002.pdf]

Patient 2

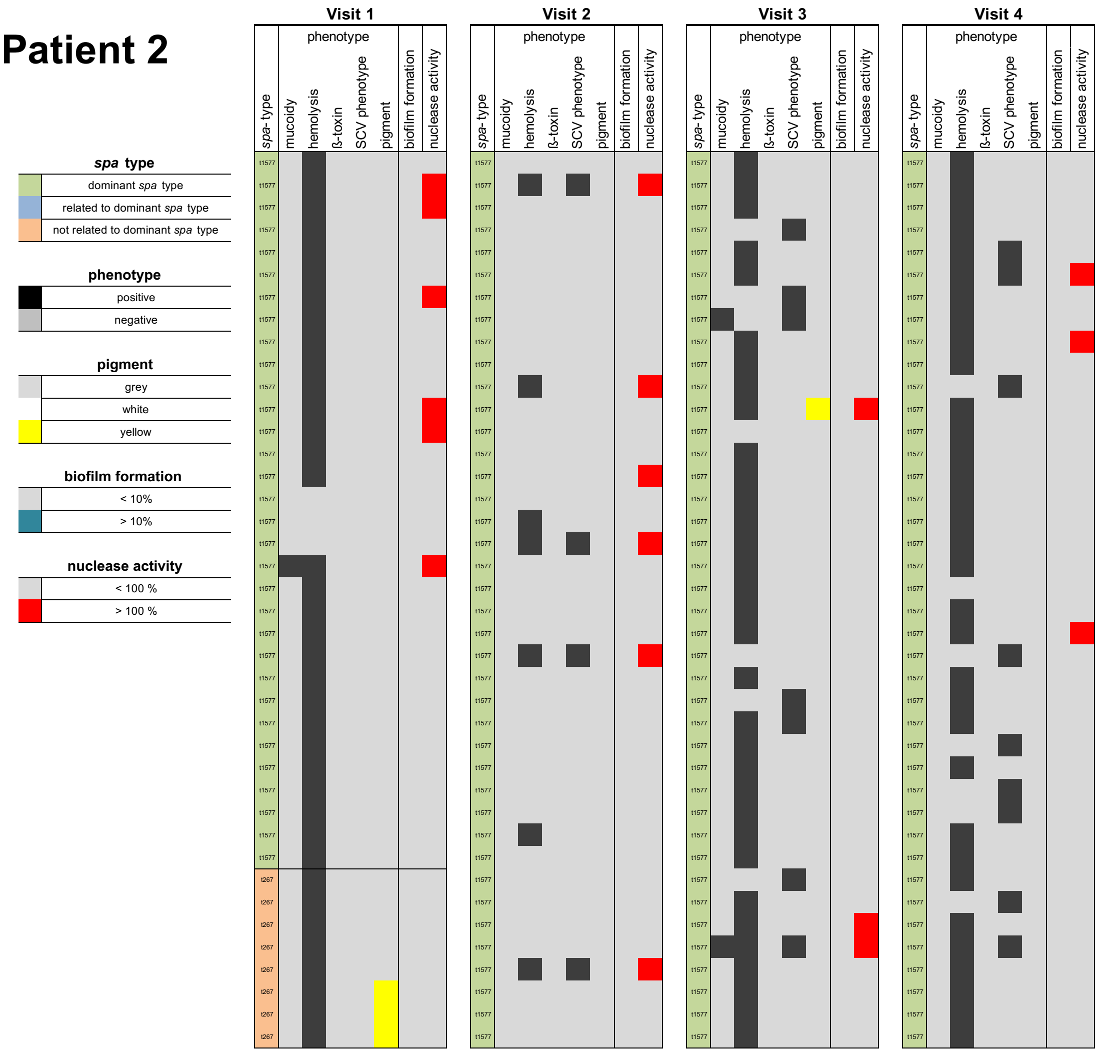

	Visit 1	Visit 2	Visit 3	Visit 4
**months from first visit**	0	2	5	8
**exacerbation**	-	-	+	+
***P. aeruginosa* coinfection**	negative	negative	negative	negative
**CFU *S. aureus***	9.6×10^7	7.2×10^7	1.5×10^6	1.6×10^7
**CFU *P. aeruginosa***	0	0	0	0

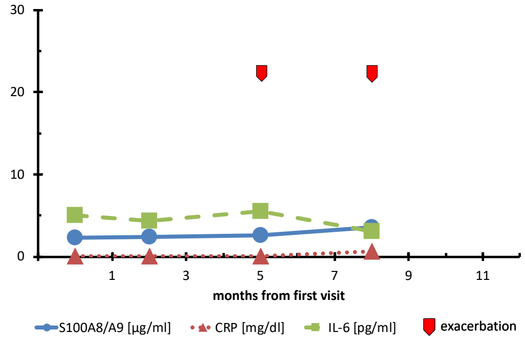

	Inflammation marker	Visit 1	Visit 2	Visit 3	Visit 4
**Serum**	S100A8/A9 [µg/ml]	2.29	2.46	2.59	3.59
	CRP [mg/dl]	0.03	0.06	0.03	0.66
	IL-6 [pg/ml]	5.08	4.38	5.57	3.11

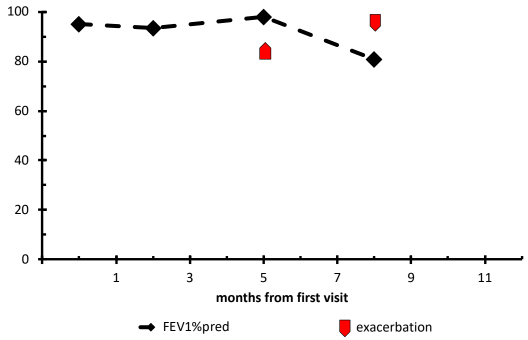

Lung function	Visit 1	Visit 2	Visit 3	Visit 4
FEV1%pred	95.08	93.46	98.08	80.77

Patient 3

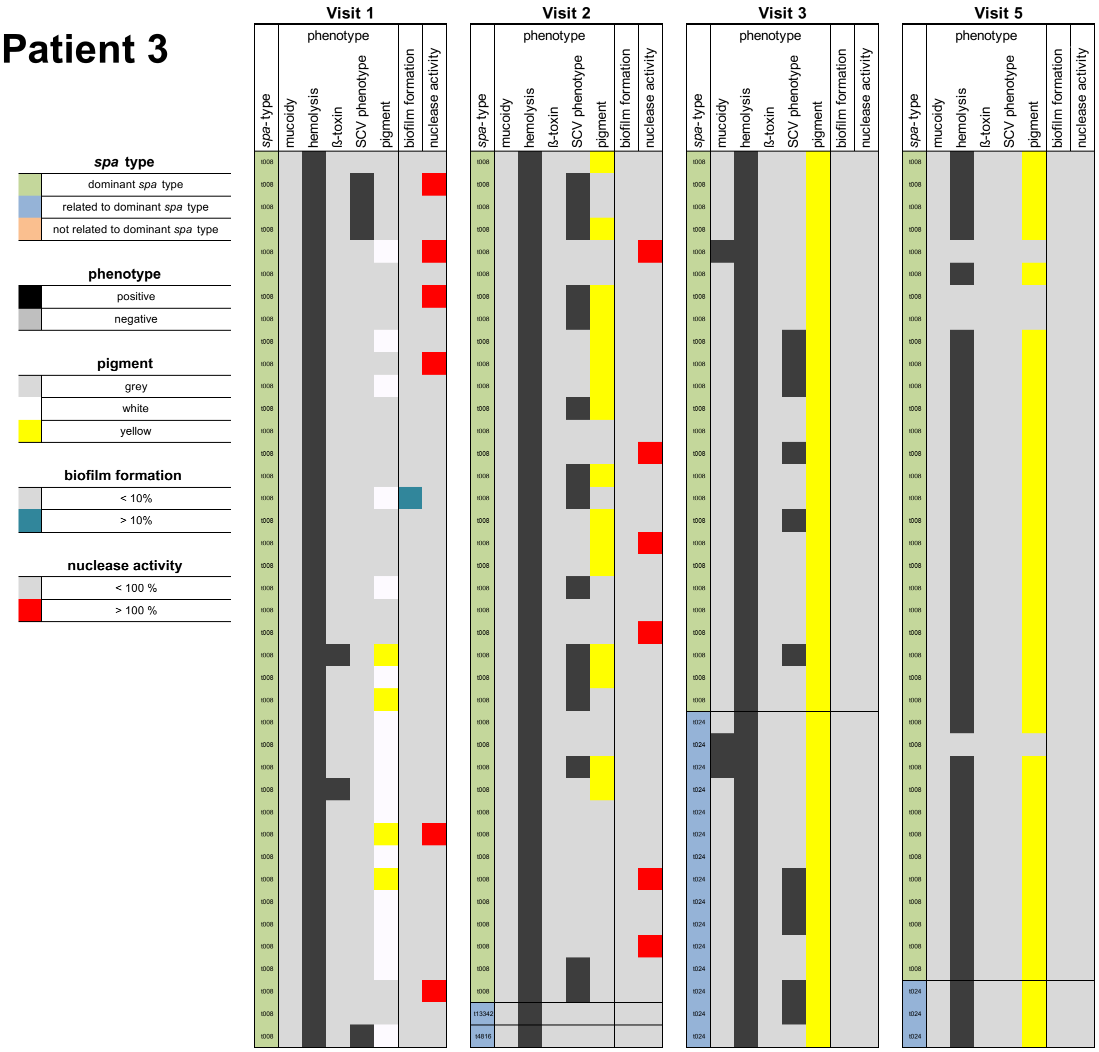

months from first visit	0	3	7	13
exacerbation	-	+	-	-
P. aeruginosa coinfection	chronic	chronic	chronic	chronic
CFU *S. aureus*	2.4×10^7	3.2×10^5	4.8×10^3	1.0×10^3
CFU *P. aeruginosa*	1.2×10^7	1.2×10^4	4.0×10^5	4.0×10^5

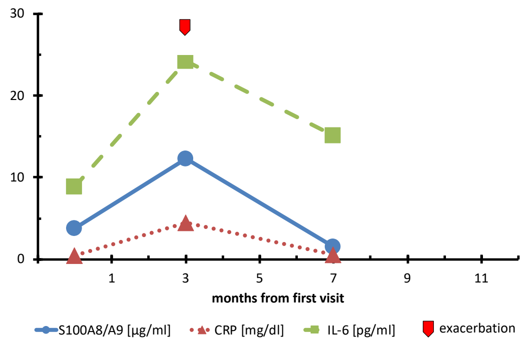

	Inflammation marker	Visit 1	Visit 2	Visit 3	Visit 5
Serum	S100A8/A9 [µg/ml]	3.80	12.31	1.58	missing
Serum	CRP [mg/dl]	0.49	4.44	0.54	missing
Serum	IL-6 [pg/ml]	8.82	24.20	15.10	missing

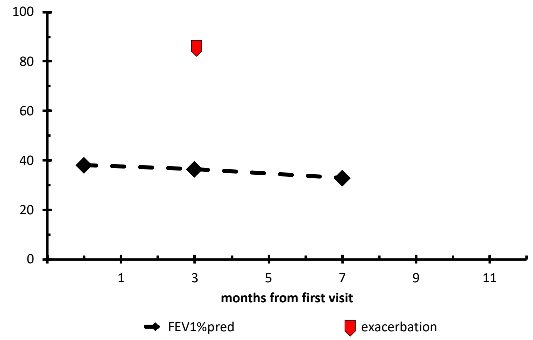

Lung function	Visit 1	Visit 2	Visit 3	Visit 5
FEV1%pred	38.03	36.36	33.03	missing

Patient 4

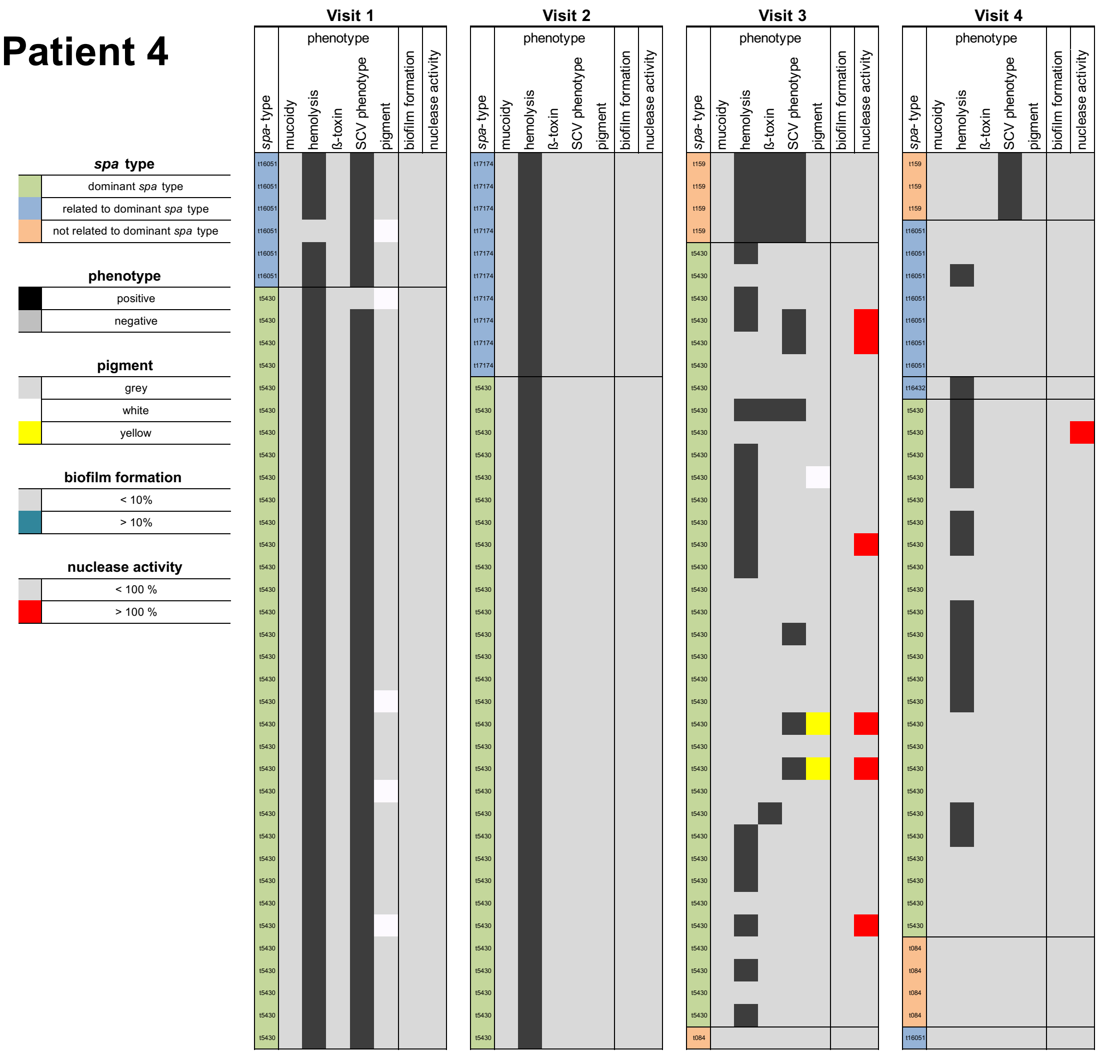

months from first visit	0	2	5	9
exacerbation	-	-	+	-
***P. aeruginosa* coinfection**	negative	negative	negative	negative
CFU ***S. aureus***	1.4×10^7	1.2×10^7	4.0×10^6	2.2×10^7
CFU ***P. aeruginosa***	0	0	0	0

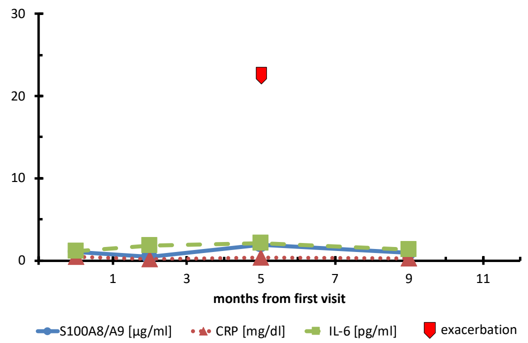

	Inflammation marker	Visit 1	Visit 2	Visit 3	Visit 4
Serum	S100A8/A9 [µg/ml]	1.04	0.40	1.93	0.90
	CRP [mg/dl]	0.48	0.14	0.30	0.20
	IL-6 [pg/ml]	1.12	1.77	2.13	1.35

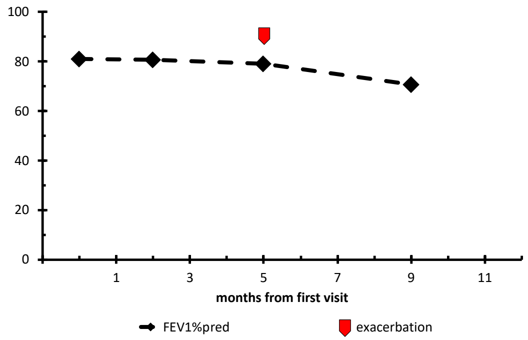

Lung function	Visit 1	Visit 2	Visit 3	Visit 4
FEV1%pred	80.93	80.85	79.23	70.80

Patient 5

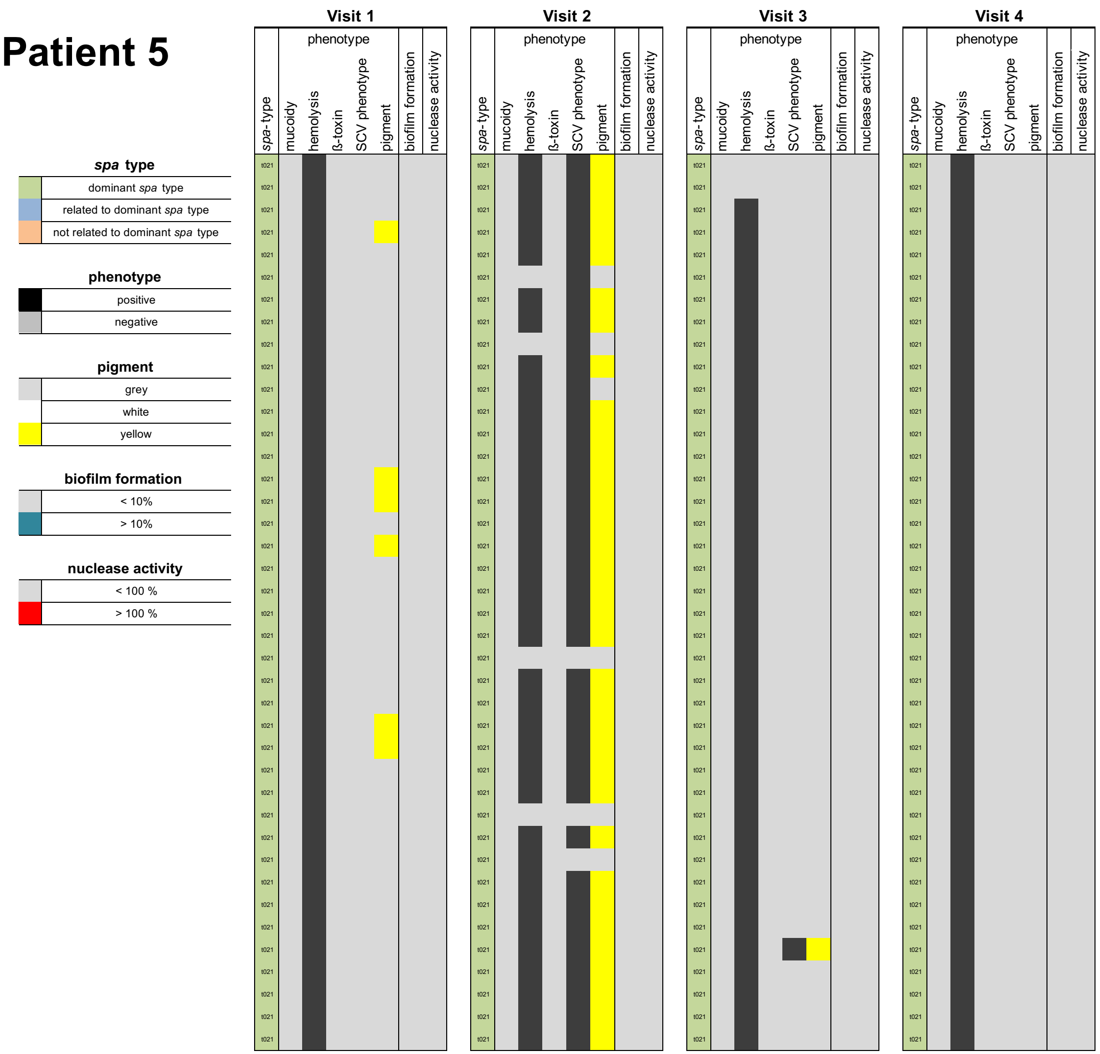

**months from first visit**	0	6	9	12
**exacerbation**	-	+	+	-
***P. aeruginosa* coinfection**	chronic	chronic	chronic	chronic
**CFU *S. aureus***	2.2×10^4	2.0×10^4	2.4×10^6	2.4×10^6
**CFU *P. aeruginosa***	4.0×10^5	8.0×10^5	1.2×10^5	1.2×10^6

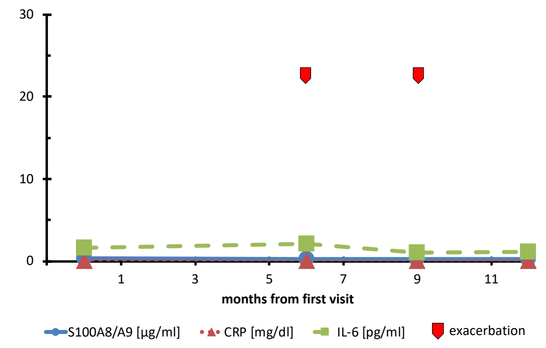

	Inflammation marker	Visit 1	Visit 2	Visit 3	Visit 4
Serum	S100A8/A9 [µg/ml]	0.35	0.24	0.22	0.22
	CRP [mg/dl]	0.05	0.03	0.03	0.05
	IL-6 [pg/ml]	1.61	2.07	1.03	1.14

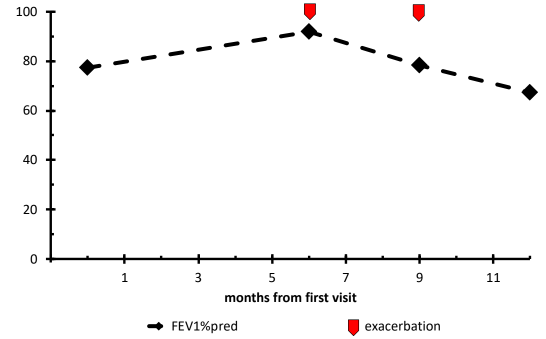

Lung function	Visit 1	Visit 2	Visit 3	Visit 4
FEV1%pred	77.41	91.93	78.23	67.35

Patient 6

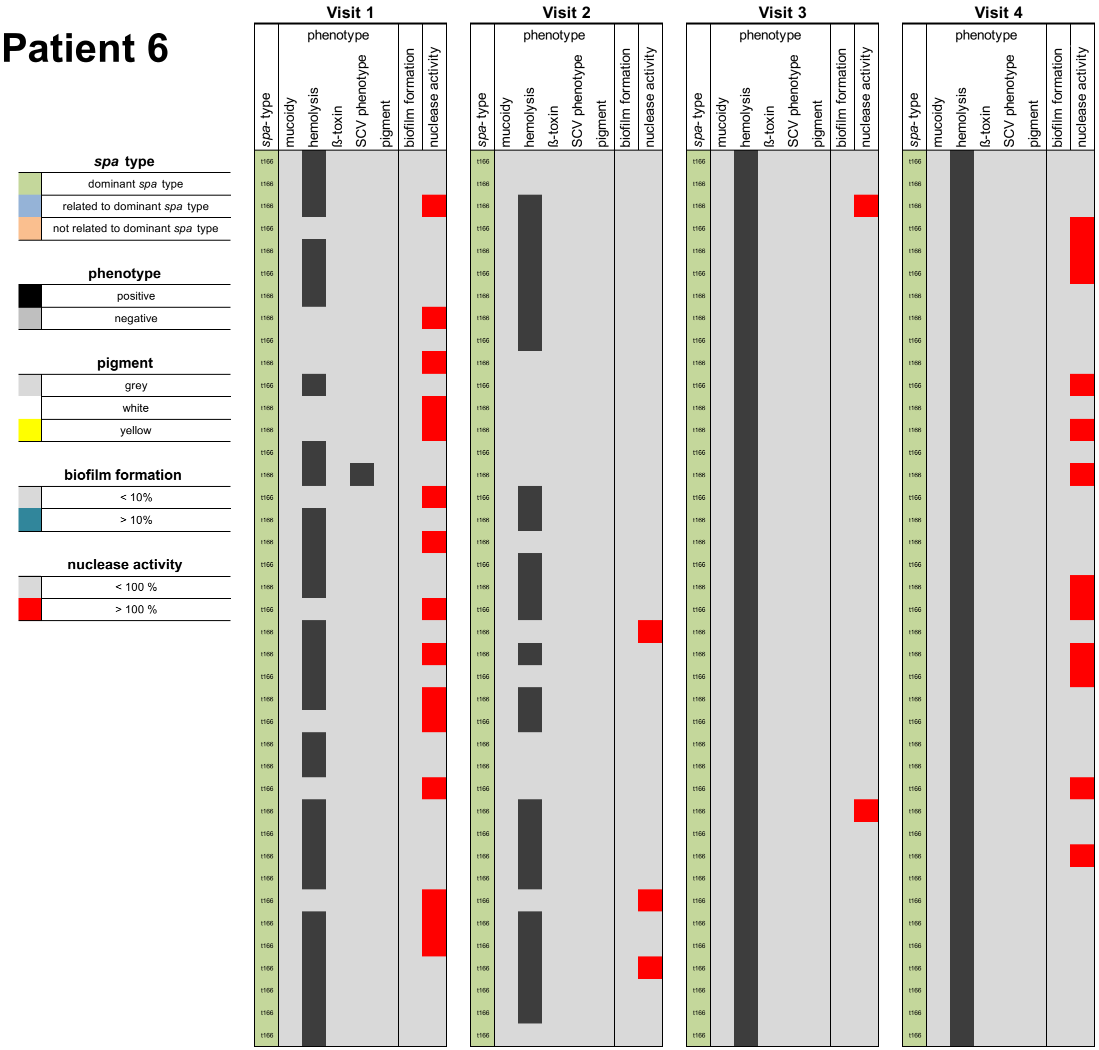

	Visit 1	Visit 2	Visit 3	Visit 4
months from first visit	0	2	6	8
exacerbation	-	+	-	-
P. aeruginosa coinfection	negative	negative	negative	negative
CFU *S. aureus*	3.6×10^6	2.0×10^5	1.6×10^7	1.6×10^7
CFU *P. aeruginosa*	0	0	0	0

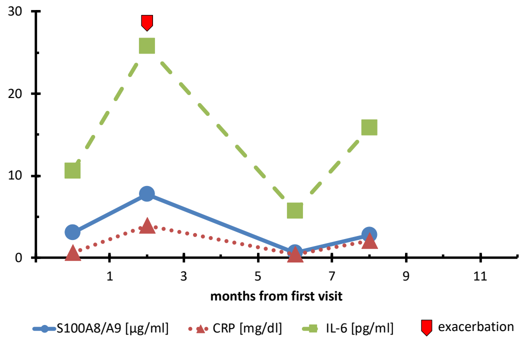

	Inflammation marker	Visit 1	Visit 2	Visit 3	Visit 4
Serum	S100A8/A9 [µg/ml]	3.05	7.74	0.61	2.77
	CRP [mg/dl]	0.60	3.91	0.39	2.08
	IL-6 [pg/ml]	10.54	25.70	5.71	15.80

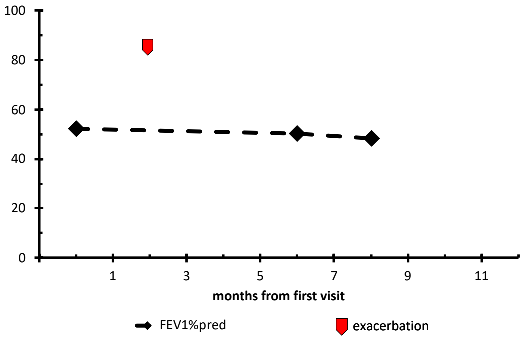

Lung function	Visit 1	Visit 2	Visit 3	Visit 4
FEV1%pred	52.18	missing	50.15	48.44

Patient 7

***spa* type**
- dominant *spa* type
- related to dominant *spa* type
- not related to dominant *spa* type

phenotype
- positive
- negative

pigment
- grey
- white
- yellow

biofilm formation
- < 10%
- > 10%

nuclease activity
- < 100 %
- > 100 %

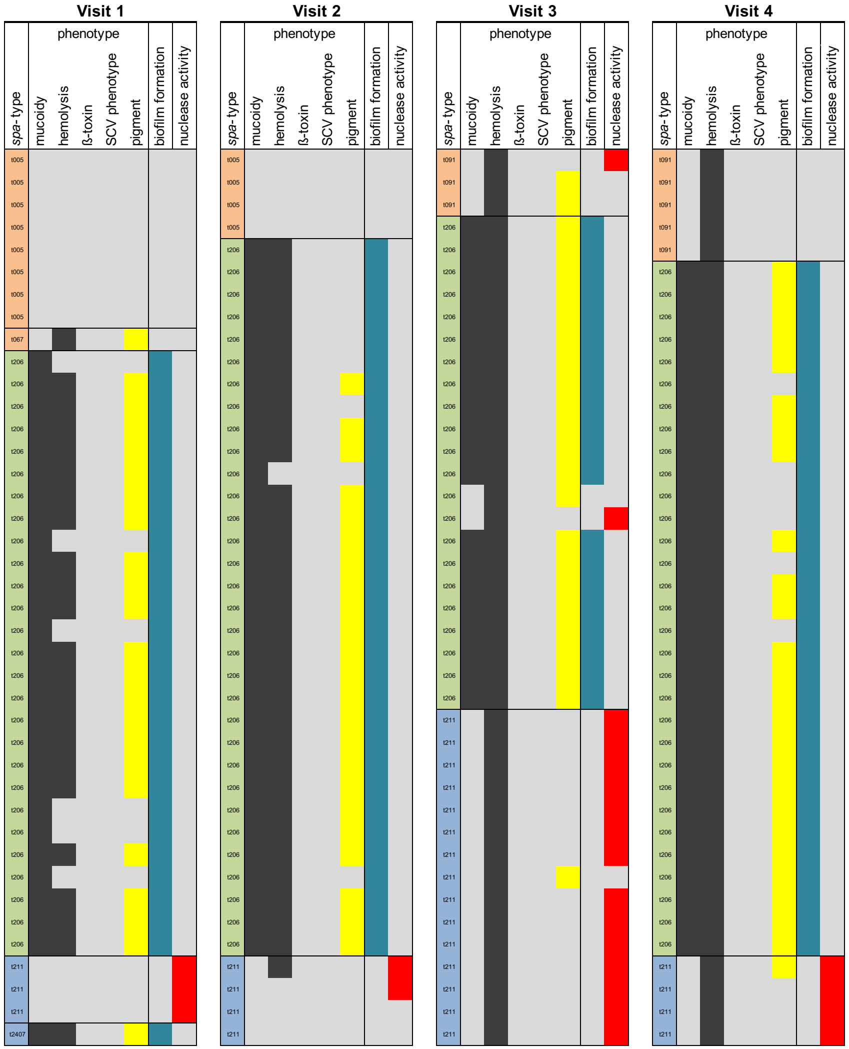

		Visit 1	Visit 2	Visit 3	Visit 4
**months from first visit**		0	2	6	10
**exacerbation**		-	-	-	+
***P. aeruginosa* coinfection**		negative	negative	negative	negative
**CFU**	***S. aureus***	1.0×10^3	4.0×10^5	2.6×10^5	1.1×10^8
	P. aeruginosa	0	0	0	0

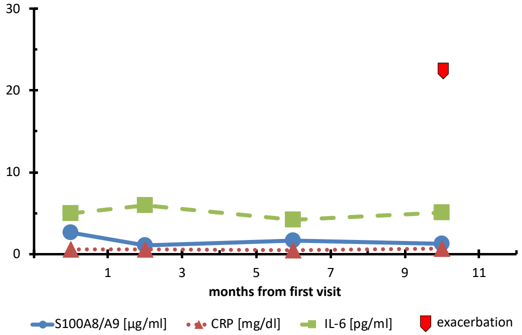

	Inflammation marker	Visit 1	Visit 2	Visit 3	Visit 4
Serum	S100A8/A9 [µg/ml]	2.58	1.08	1.62	1.22
	CRP [mg/dl]	0.51	0.52	0.43	0.68
	IL-6 [pg/ml]	4.97	5.93	4.22	5.01

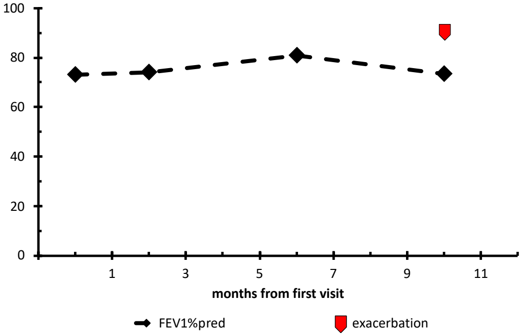

Lung function	Visit 1	Visit 2	Visit 3	Visit 4
FEV1%pred	73.19	74.15	80.92	73.51

Patient 8

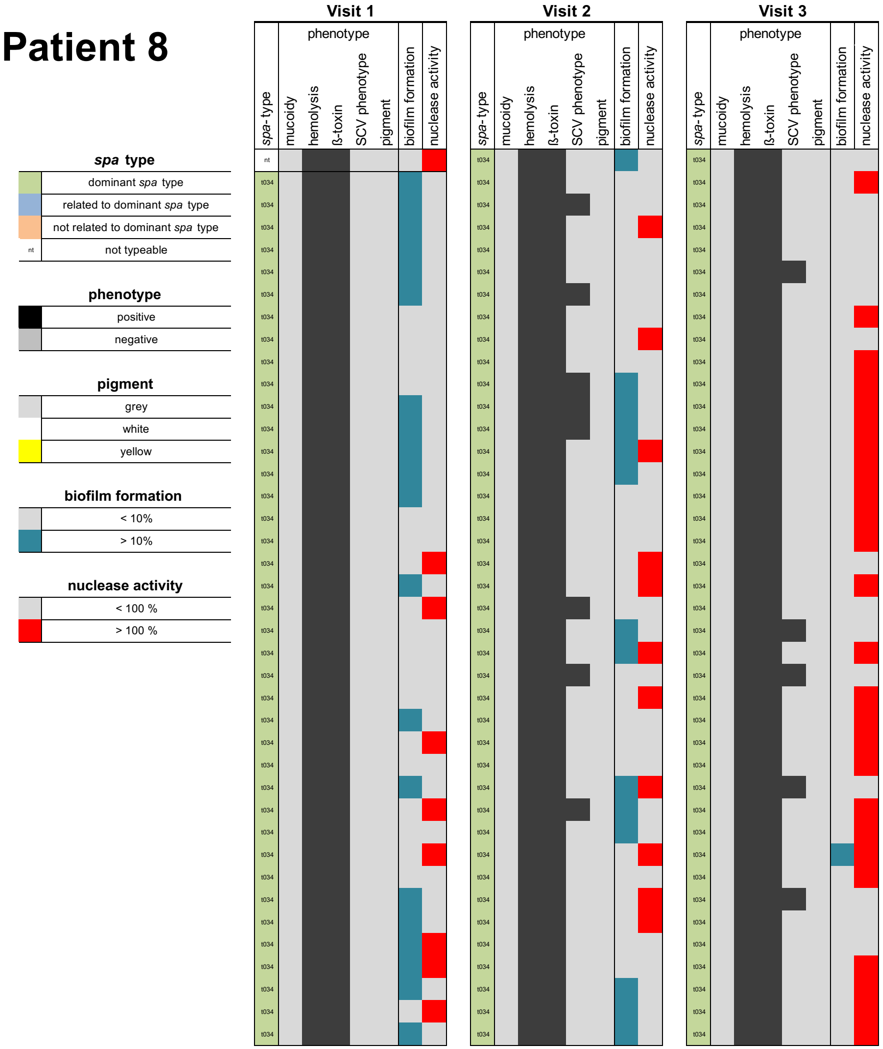

		Visit 1	Visit 2	Visit 3
months from first visit		0	3	9
exacerbation		-	-	-
P. aeruginosa coinfection		negative	negative	negative
CFU	*S. aureus*	4.0×10^6	2.6×10^9	1.0×10^4
	P. aeruginosa	0	0	0

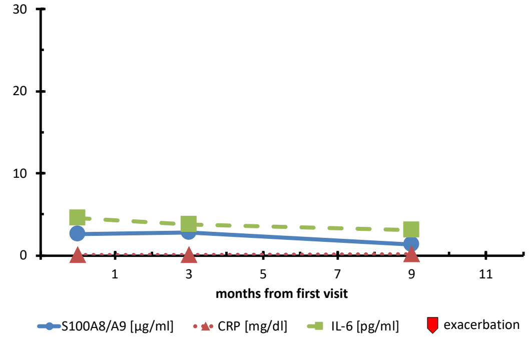

	Inflammation marker	Visit 1	Visit 2	Visit 3
Serum	S100A8/A9 [µg/ml]	2.62	2.77	1.33
	CRP [mg/dl]	0.07	0.03	0.16
	IL-6 [pg/ml]	4.50	3.77	3.10

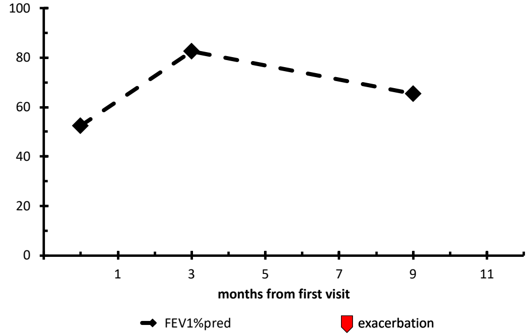

Lung function	Visit 1	Visit 2	Visit 3
FEV1%pred	52.54	82.63	65.48

Patient 10

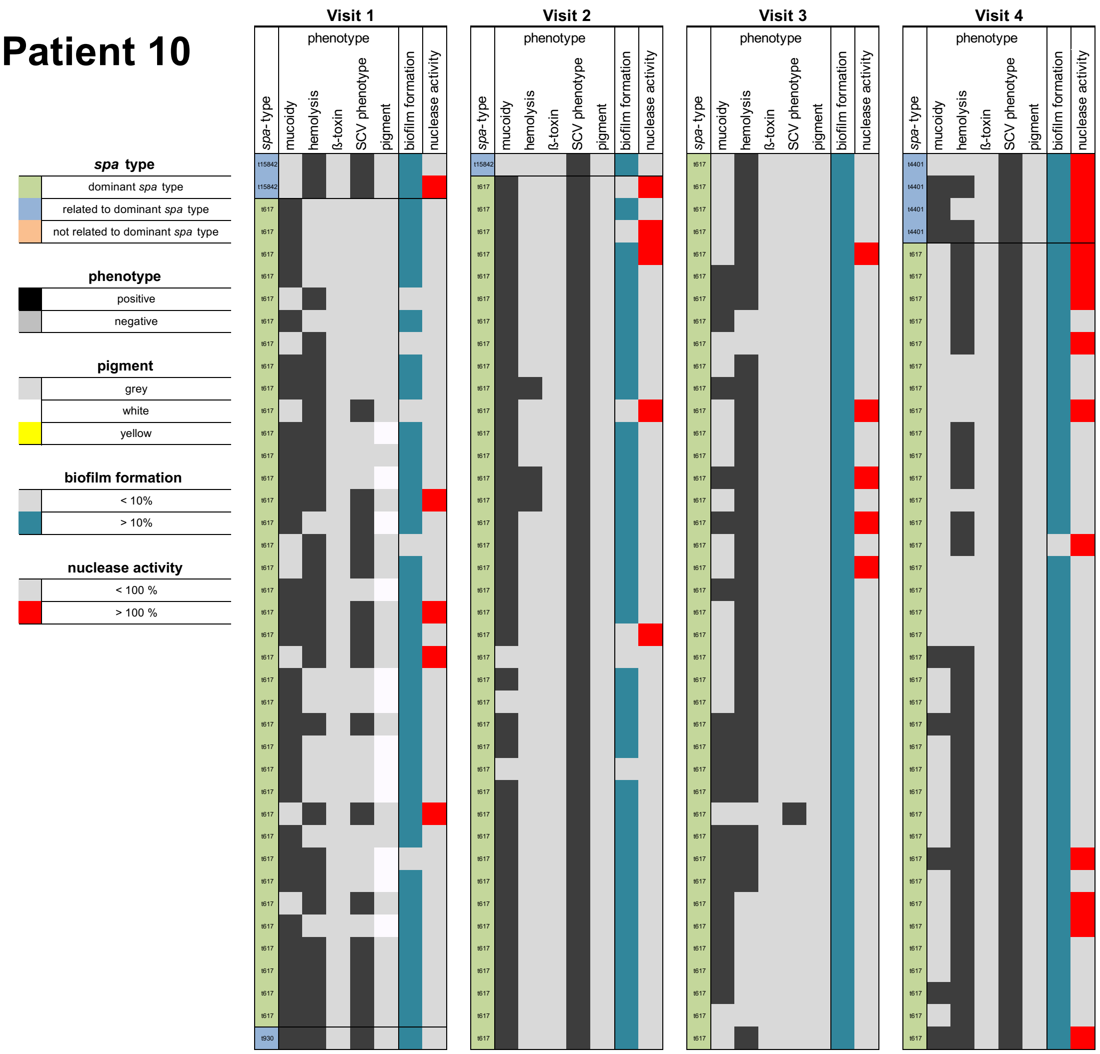

	Visit 1	Visit 2	Visit 3	Visit 4
months from first visit	0	3	6	10
exacerbation	-	+	-	-
P. aeruginosa coinfection	negative	negative	negative	negative
CFU *S. aureus*	3.6×10^5	3.6×10^7	7.0×10^5	1.8×10^6
CFU *P. aeruginosa*	0	0	0	0

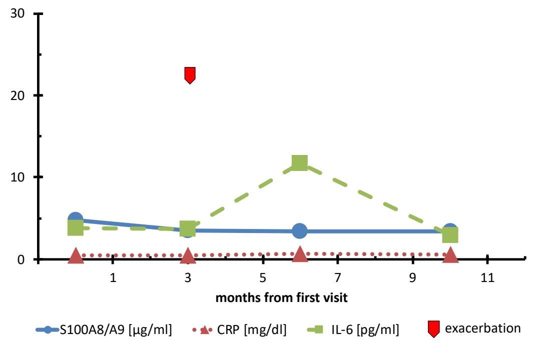

	Inflammation marker	Visit 1	Visit 2	Visit 3	Visit 4
Serum	S100A8/A9 [µg/ml]	4.77	3.54	3.40	3.42
	CRP [mg/dl]	0.46	0.44	0.63	0.55
	IL-6 [pg/ml]	3.82	3.72	11.73	2.93

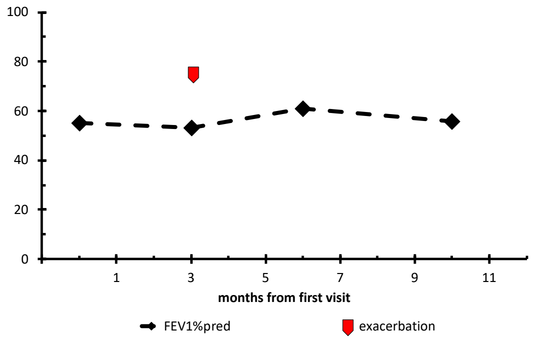

Lung function	Visit 1	Visit 2	Visit 3	Visit 4
FEV1%pred	55.15	53.08	61.12	55.88

Patient 11

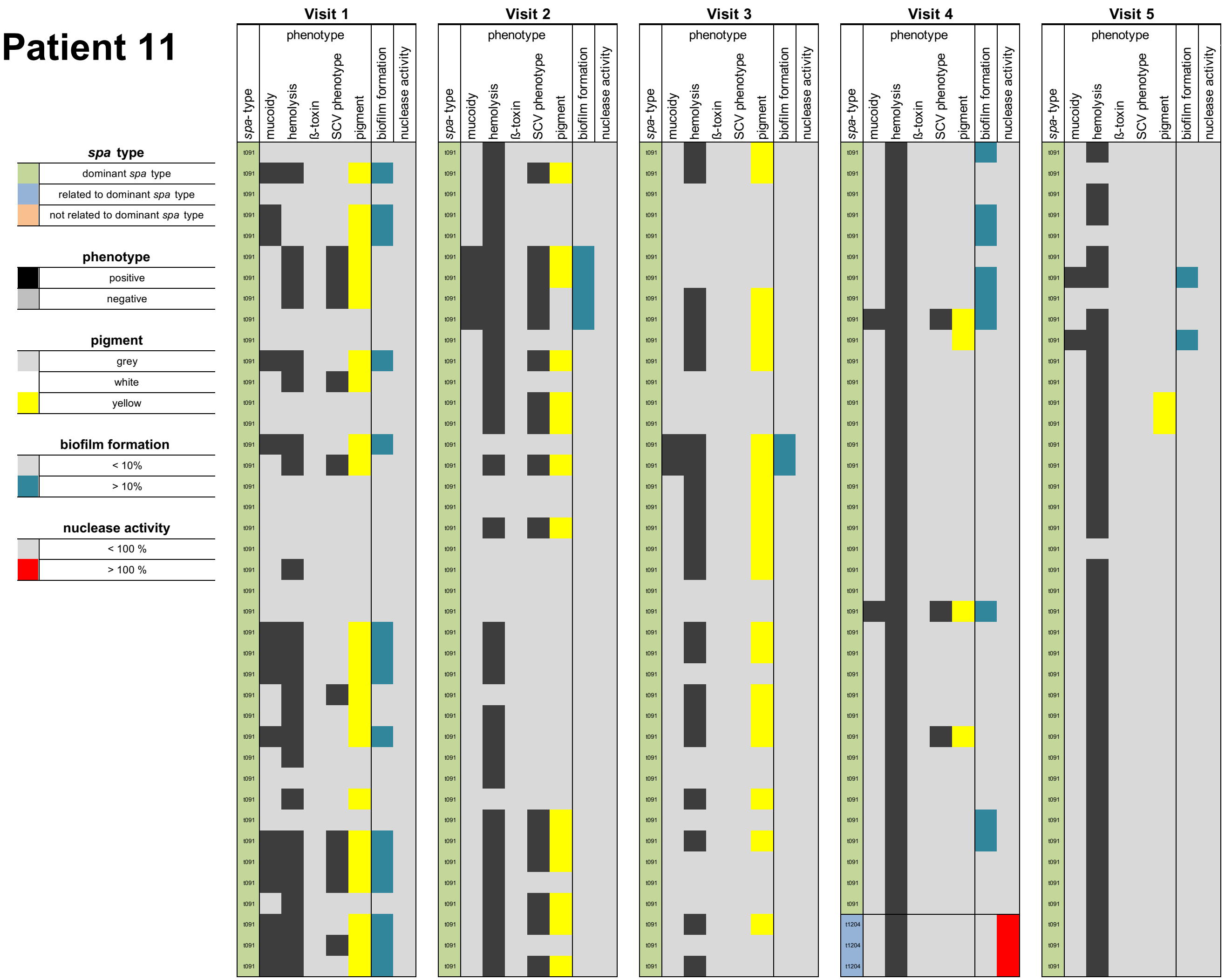

		Visit 1	Visit 2	Visit 3	Visit 4	Visit 5
months from first visit		0	2	4	7	9
exacerbation		-	+	-	-	-
P. aeruginosa coinfection		negative	negative	positive	positive	negative
CFU	*S. aureus*	3.2×10^7	2.0×10^8	1.2×10^7	1.6×10^6	6.4×10^6
	P. aeruginosa	0	0	2.6×10^4	1.0×10^5	0

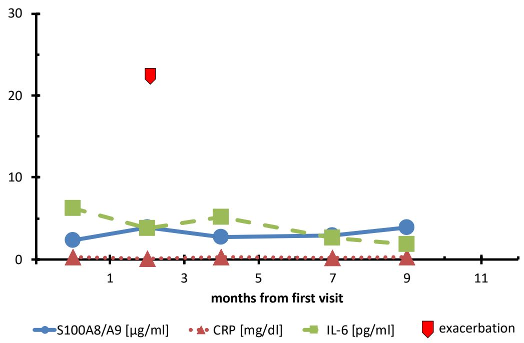

	Inflammation marker	Visit 1	Visit 2	Visit 3	Visit 4	Visit 5
Serum	S100A8/A9 [µg/ml]	2.33	3.88	2.69	2.97	3.86
	CRP [mg/dl]	0.29	0.13	0.30	0.15	0.24
	IL-6 [pg/ml]	6.28	3.78	5.13	2.59	1.82

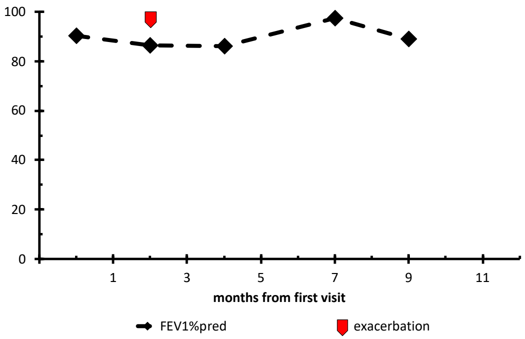

Lung function	Visit 1	Visit 2	Visit 3	Visit 4	Visit 5
FEV1%pred	90.43	86.48	86.24	97.47	89.01

Patient 12

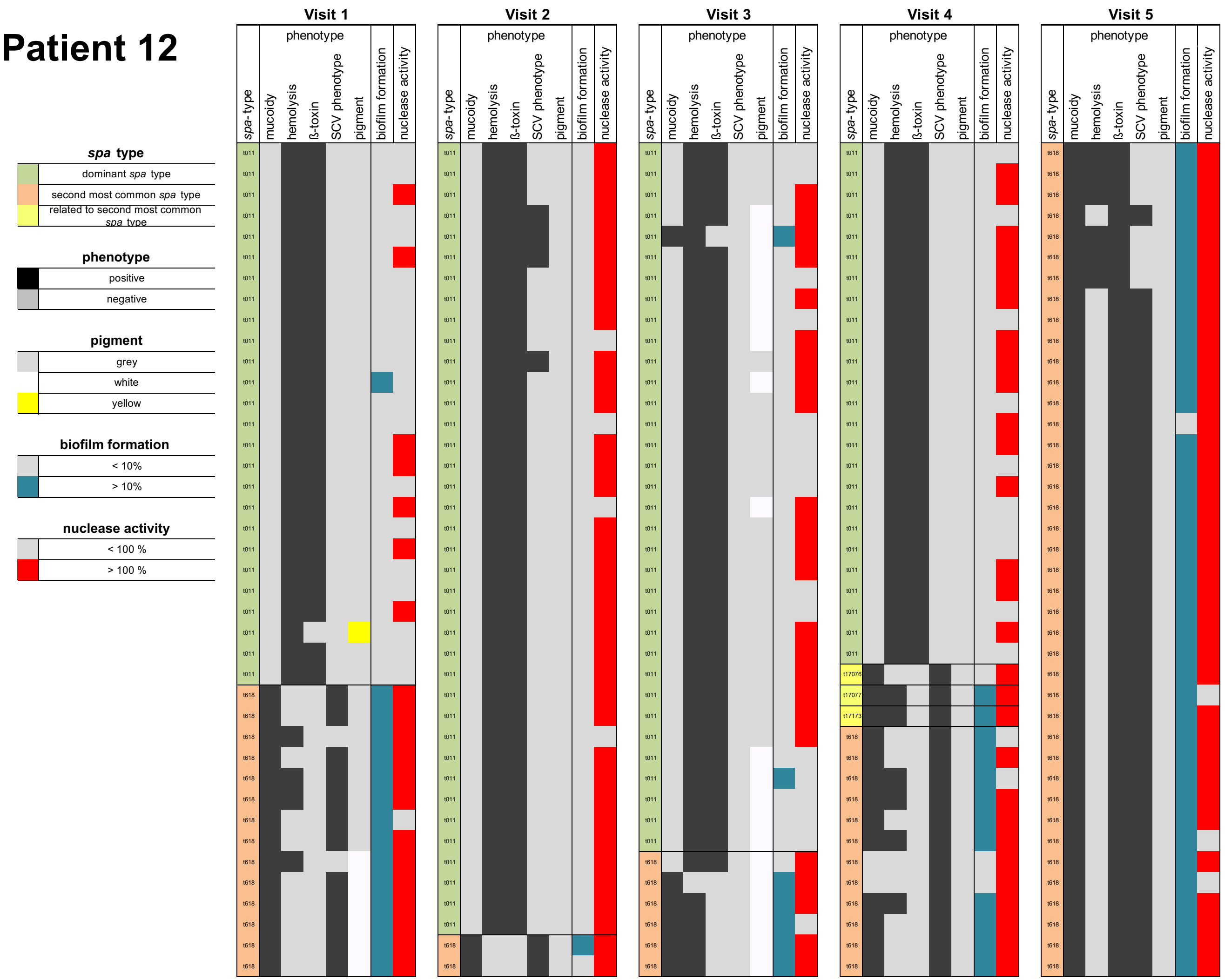

		Visit 1	Visit 2	Visit 3	Visit 4	Visit 5
months from first visit		0	1	2	8	12
exacerbation		+	+	+	+	-
P. aeruginosa coinfection		chronic	chronic	chronic	chronic	chronic
CFU	*S. aureus*	1.0×10^3	3.2×10^7	2.4×10^7	2.0×10^5	1.0×10^4
	P. aeruginosa	9.2×10^7	1.5×10^8	2.6×10^8	1.2×10^7	1.6×10^7

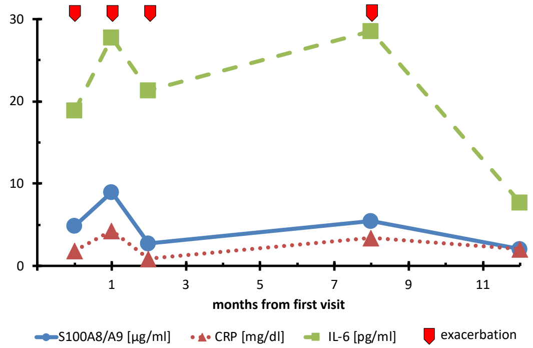

	Inflammation marker	Visit 1	Visit 2	Visit 3	Visit 4	Visit 5
Serum	S100A8/A9 [µg/ml]	4.89	8.96	2.76	5.44	2.08
	CRP [mg/dl]	1.89	4.31	0.91	3.42	2.01
	IL-6 [pg/ml]	18.90	27.80	21.30	28.50	7.73

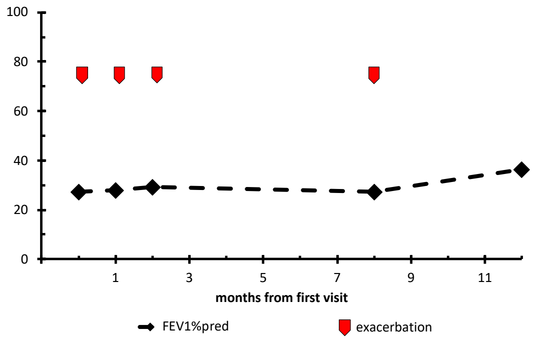

Lung function	Visit 1	Visit 2	Visit 3	Visit 4	Visit 5
FEV1%pred	27.23	27.87	29.29	27.36	36.39

Patient 13

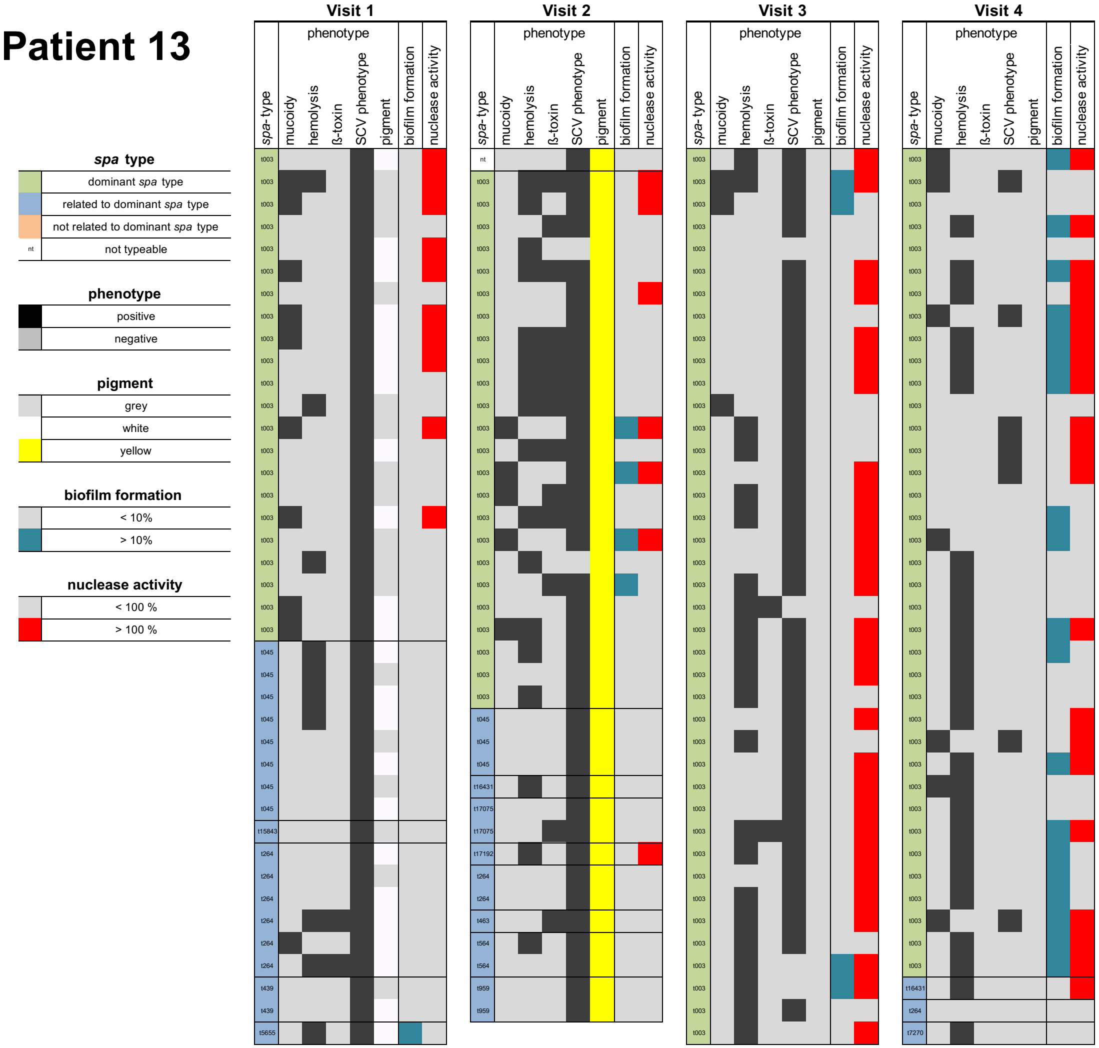

	Visit 1	Visit 2	Visit 3	Visit 4
months from first visit	0	3	6	9
exacerbation	-	-	-	-
P. aeruginosa coinfection	chronic	chronic	chronic	chronic
CFU *S. aureus*	4.4×10^{7}	2.8×10^{6}	4.0×10^{6}	2.7×10^{5}
CFU *P. aeruginosa*	1.6×10^{8}	3.0×10^{7}	2.0×10^{7}	2.4×10^{7}

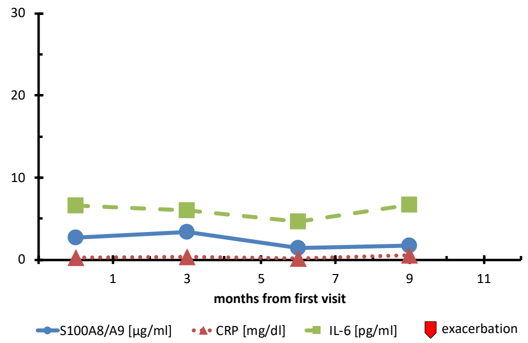

	Inflammation marker	Visit 1	Visit 2	Visit 3	Visit 4
Serum	S100A8/A9 [µg/ml]	2.67	3.36	1.41	1.75
	CRP [mg/dl]	0.25	0.34	0.17	0.53
	IL-6 [pg/ml]	6.60	6.03	4.63	6.68

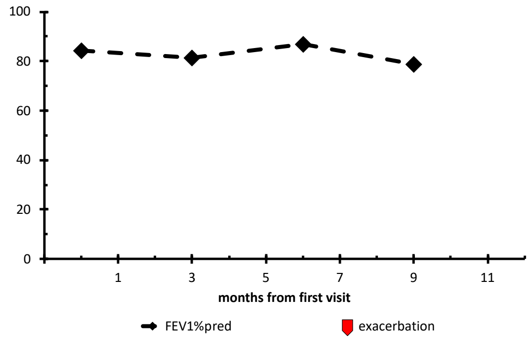

Lung function	Visit 1	Visit 2	Visit 3	Visit 4
FEV1%pred	84.42	81.53	86.83	78.94

Patient 14

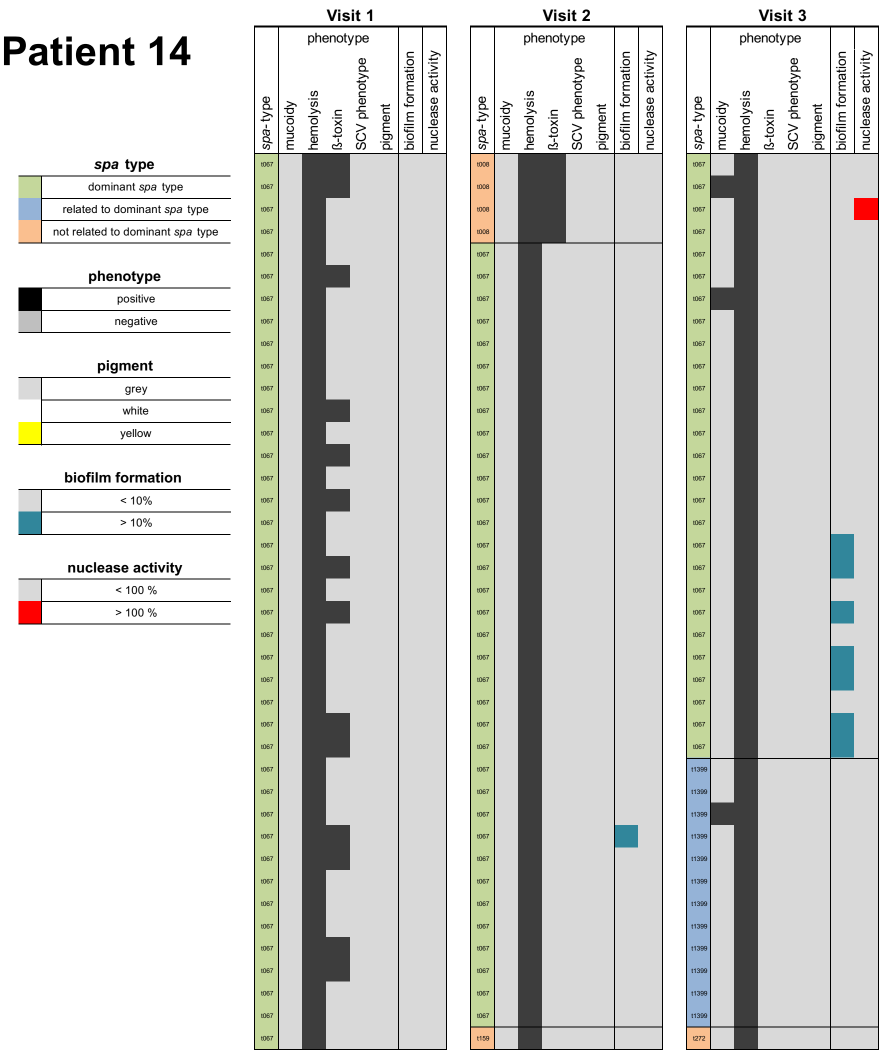

	Visit 1	Visit 2	Visit 3
months from first visit	0	6	11
exacerbation	+	+	-
P. aeruginosa coinfection	chronic	chronic	chronic
CFU *S. aureus*	1.2×10^{6}	2.0×10^{7}	2.0×10^{7}
CFU *P. aeruginosa*	7.6×10^{7}	2.4×10^{7}	1.4×10^{8}

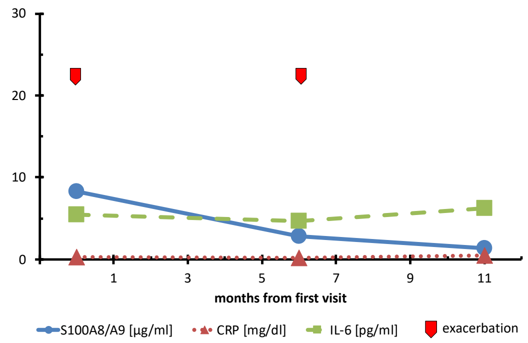

	Inflammation marker	Visit 1	Visit 2	Visit 3
Serum	S100A8/A9 [µg/ml]	8.34	2.82	1.39
	CRP [mg/dl]	0.26	0.23	0.48
	IL-6 [pg/ml]	5.42	4.69	6.24

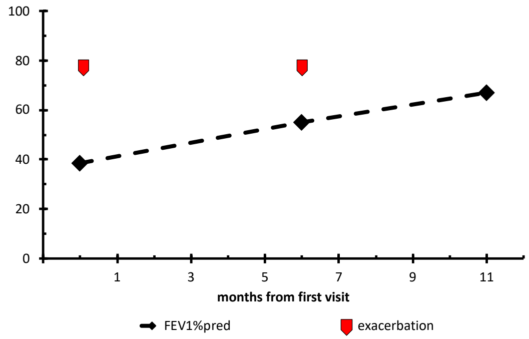

Lung function	Visit 1	Visit 2	Visit 3
FEV1%pred	38.45	55.21	67.01
