# Supplementary figures and images for: Association of Diverse Staphylococcus aureus Populations with Pseudomonas aeruginosa Coinfection and Inflammation in Cystic Fibrosis Airway Infection
Source: mSphere. 2021 Jun 23;6(3):e00358-21. doi: 10.1128/mSphere.00358-21 (PMC8265651; doi:10.1128/mSphere.00358-21)

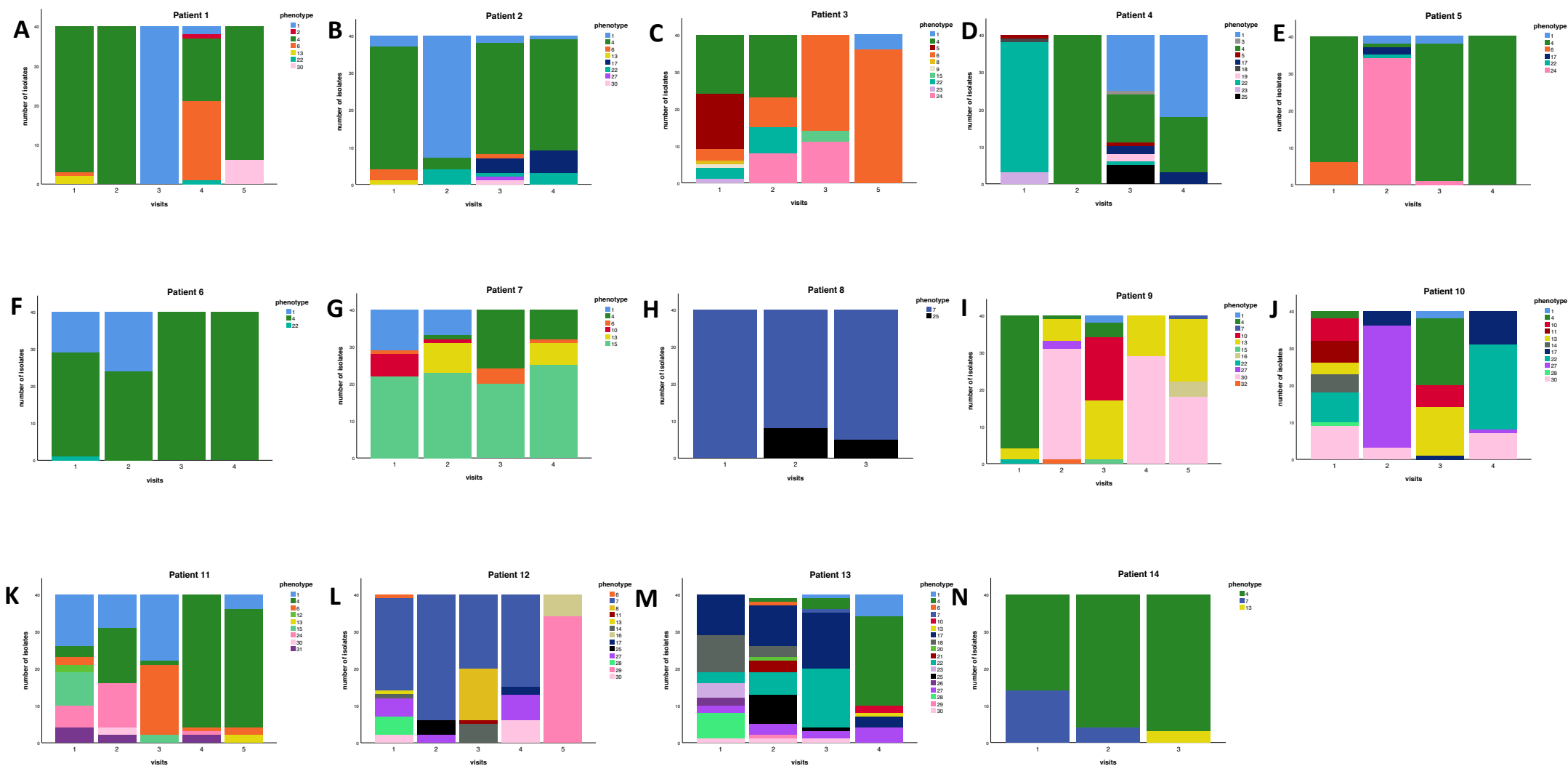

Supplement: FIG S3 [file msphere.00358-21-sf003.pdf]

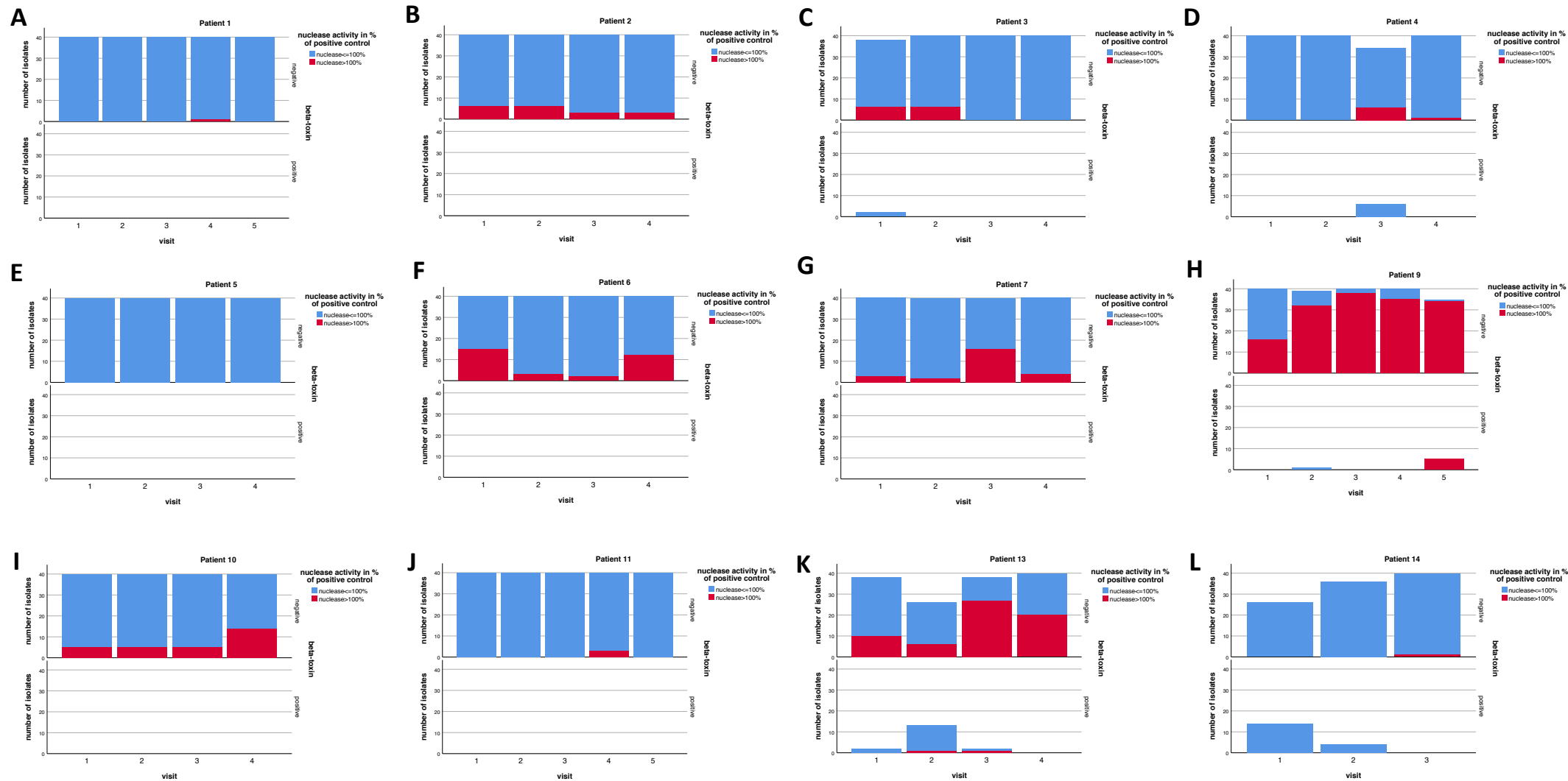

Supplement: FIG S4 [file msphere.00358-21-sf004.pdf]
