# Supplementary material for: Association of Diverse Staphylococcus aureus Populations with Pseudomonas aeruginosa Coinfection and Inflammation in Cystic Fibrosis Airway Infection
Source: mSphere. 2021 Jun 23;6(3):e00358-21. doi: 10.1128/mSphere.00358-21 (PMC8265651; doi:10.1128/mSphere.00358-21)
Supplement: TABLE S2 [file msphere.00358-21-st002.docx]

**Table S2:** ***S. aureus* *spa*-types, respective phenotypes cultured**

| ***spa-*type** | **Pheno-**  **Types**^*^ | **Isolates^†^** | **Patients**^‡^ |
| --- | --- | --- | --- |
| t091 | 11 | 397 | P1, P7, P11 |
| t002 | 11 | 195 | P9 |
| t021 | 6 | 160 | P5 |
| t166 | 3 | 160 | P6 |
| t617 | 11 | 152 | P10 |
| t1577 | 8 | 152 | P2 |
| t008 | 11 | 144 | P3, P14 |
| t003 | 16 | 123 | P13 |
| t5430 | 9 | 123 | P4 |
| t011 | 5 | 123 | P12 |
| t034 | 2 | 119 | P8 |
| t206 | 5 | 112 | P7 |
| t067 | 4 | 103 | P7, P14 |
| t618 | 10 | 74 | P12 |
| t211 | 3 | 26 | P7 |
| t005 | 3 | 20 | P1, P7 |
| t024 | 3 | 18 | P3 |
| t16051 | 4 | 14 | P4 |
| t1399 | 2 | 12 | P14 |
| t045 | 4 | 11 | P13 |
| t17174 | 1 | 10 | P4 |
| t264 | 5 | 9 | P13 |
| t159 | 3 | 8 | P4, P14 |
| t267 | 2 | 8 | P2 |
| t084 | 1 | 5 | P4 |
| t4401 | 3 | 4 | P10 |
| t15842 | 2 | 3 | P10 |
| t062 | 1 | 3 | P9 |
| t1204 | 1 | 3 | P11 |
| nicht typisierbar | 2 | 2 | P8, P13 |
| t16431 | 2 | 2 | P13 |
| t17075 | 2 | 2 | P13 |
| t439 | 2 | 2 | P13 |
| t564 | 2 | 2 | P13 |
| t959 | 2 | 2 | P13 |
| t13342 | 1 | 1 | P3 |
| t15843 | 1 | 1 | P13 |
| t16432 | 1 | 1 | P4 |
| t17076 | 1 | 1 | P12 |
| t17077 | 1 | 1 | P12 |
| t17173 | 1 | 1 | P12 |
| t17192 | 1 | 1 | P13 |
| t2202 | 1 | 1 | P9 |
| t2407 | 1 | 1 | P7 |
| t272 | 1 | 1 | P14 |
| t3127 | 1 | 1 | P9 |
| t463 | 1 | 1 | P13 |
| t4816 | 1 | 1 | P3 |
| t5655 | 1 | 1 | P13 |
| t7270 | 1 | 1 | P13 |
| t930 | 1 | 1 | P10 |

^*^ number of different phenotypes observed in these *spa*-types

^†^ number of *S. aureus* isolates with the respective *spa-*type

^‡^ patients carrying these *spa*-types
